# Complexation of Ln(III) Ions by Gluconate: Joint Investigation Applying TRLFS, CE-ICP-MS, NMR, and DF Calculations

**DOI:** 10.1021/acs.inorgchem.4c05476

**Published:** 2025-04-11

**Authors:** Sophie Zenker, Janik Lohmann, Ion Chiorescu, Sven Krüger, Michael U. Kumke, Tobias Reich, Katja Schmeide, Jerome Kretzschmar

**Affiliations:** † Institute of Chemistry, Universität Potsdam, Potsdam 14476, Germany; ‡ Department of Chemistry, Johannes Gutenberg-Universität Mainz, Mainz 55128, Germany; § Chemistry Department, School of Natural Sciences, Technische Universität München, Garching 85748, Germany; ∥ Institute of Resource Ecology, Helmholtz-Zentrum Dresden–Rossendorf, Dresden 01328, Germany

## Abstract

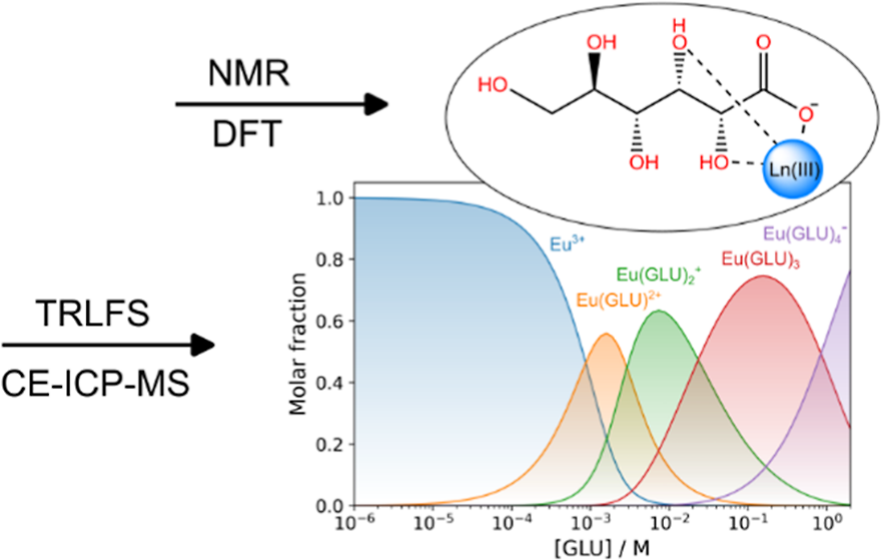

The potential of gluconate, a common cement additive, to mobilize lanthanides (used as analogues of actinides) from cement is investigated. For this purpose, complex formation of trivalent lanthanides, Ln(III), (Ln: La, Sm, Eu, Gd, Lu) with gluconate (GLU) was studied applying time-resolved laser-induced luminescence spectroscopy (TRLFS) in combination with parallel factor analysis (PARAFAC), capillary electrophoresis-inductively coupled plasma mass spectrometry (CE-ICP-MS), nuclear magnetic resonance (NMR) spectroscopy, and density functional (DF) calculations. Up to circumneutral conditions, binary complexes form with Ln(III):GLU stoichiometric ratios of 1:1–1:4 depending only on the Ln:GLU ratio, regardless of the concentration regime (micromolar to millimolar). Coordination facilitates via the carboxyl group (C1) and the adjacent hydroxyl group (at C2) forming a five-membered ring chelation motif, with a probable participation of the C3 hydroxyl group. Beyond circumneutral pH, with the exact onset depending on the specific lanthanide, a fundamental change in speciation takes place. Speciation then becomes more complex upon coexistence and interconversion of several (isomeric) complexes concluded to involve one or more deprotonated GLU hydroxyl groups not necessarily participating in coordination.

## Abstract 2

The methodological approach in this study allowed for extended insights into the complexation behavior of trivalent lanthanides toward gluconate, the resulting speciation, and corresponding structure–property relationships, covering several orders of magnitude in concentration and Ln(III):GLU ratios for aqueous solutions in the pH range 4 through 13.

## Introduction

Cementitious materials are used in repositories for radioactive waste as construction, sealing, and backfill materials as well as for the solidification of low- and intermediate-level radioactive waste and are thus part of the multibarrier system of a repository. Organic compounds are used as admixtures in cement-based materials (e.g., citrate, gluconate, superplasticizers such as polycarboxylate ether) to ensure a good workability of the cementitious materials and good mechanical properties of the final concrete.^1−3^ Moreover, organic materials may also be present in the radioactive waste (e.g., decontamination and cleaning agents).^4−6^ The release of such organic compounds or their radiolytic, hydrolytic, or microbial degradation products due to ingress of water may lead to a mobilization of radionuclides from radioactive waste in case of a container failure and potentially to a deterioration of the function of geotechnical barriers.^6,7^ Therefore, the effect of organic ligands on radionuclide retention by various repository-relevant barrier materials is of great interest. It has been found to be influenced by, e.g., ionic strength and pH.^8−13^

To improve the understanding of such systems and to determine the specific role of organic ligands for radionuclide mobility in cement-based systems, the radionuclide–organic ligand complex formation in solution must be studied over a wide pH range up to the typical hyperalkaline conditions of cement pore waters.

**Figure 1:**
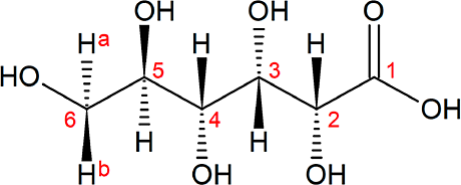
Generic structure of d-gluconic acid along with atom assignment.

Gluconate (GLU, Figure 1), a polyhydroxycarboxylic acid, has been shown to complex and mobilize radionuclides.^14−18^ The potential of the GLU-mediated mobilization will depend on chemical parameters such as ionic strength and pH value. Alkaline conditions are of importance in the repository context since upon incidental water intrusion, alkaline pore waters are present in the cement and will subsequently determine the conditions for complexation of radionuclides with GLU. Consequently, in pore water of cement at degradation stages II and III, GLU will represent a potent complexing agent interfering with the retardation of radionuclides in the cement.

Various data have been reported on metal ion interaction with GLU. For instance, complexation of Pr(III) was studied between pH 2 and 11.5 using potentiometry, UV–vis spectrophotometry, circular dichroism, and NMR,^19^ of Nd(III) in the pH range of 2–8 using spectrophotometry, potentiometry, conductometry, and NMR^18^ as well as in the pH range of 5–13 using NMR, circular dichroism (CD), and Raman spectroscopies,^20^ and of La(III), Eu(III), Dy(III), Er(III), and Lu(III) by means of potentiometry between pH 2 and 11 as well as NMR at pH 4.^21^ As a synopsis from the literature dealing with Ln(III)–GLU systems, when surpassing circumneutral pH conditions, speciation appears to be rather complex upon coexistence of several species some of which being polynuclear.^18,20,21^ All of these studies apply Ln(III) concentrations in the millimolar range (broadly speaking, 1.3–75 mM). It is thus not surprising that polynuclear complexes form. Therefore, our investigations include the micromolar concentration range, which is also more relevant for safety considerations, and apply analytic techniques unprecedented to the Ln(III)–GLU system such as time-resolved laser-induced luminescence spectroscopy (TRLFS) in combination with parallel factor analysis (PARAFAC) as well as capillary electrophoresis-inductively coupled plasma mass spectrometry (CE-ICP-MS).

Under the reducing conditions expected to prevail in a repository, the radionuclides, especially the actinides, are present in the oxidation states +III and +IV. In this study, we investigate the interaction between GLU and Ln(III), as natural analogues for trivalent actinides, by examination of speciation and the complex structure. Therefore, we used a combination of complementary analytical methods (TRLFS, CE-ICP-MS, and NMR) to address metal- as well as ligand-related aspects of the complexation reaction, complemented by density functional (DF) calculations. In order to exploit the specific analytical power of each method, the Ln(III) and GLU concentrations were varied over a large concentration range, allowing tailoring of the molar ratio of Ln(III):GLU over several orders of magnitude for the investigation of stoichiometry and nuclearity. CE-ICP-MS can provide information about the charge of the complexes in solution and thus about their stoichiometry. Using CE-ICP-MS at Ln(III) concentrations as low as 1 μM allows for experiments in the absence of polynuclear complexes simplifying speciation. In this way, a ligand excess of several orders of magnitude can be achieved. The measured electrophoretic mobilities are proportional to the mean charge in solution, which is dependent on the ligand concentration and allows for the determination of complex formation constants. On the other hand, TRLFS (due to the unique spectroscopic properties of Eu(III)) can be carried out for a larger concentration range bridging the CE-ICP-MS and NMR experiments. From the temporal-spectral analysis, different species in the system can be identified. Moreover, TRLFS is applicable to both solutions and solids (or to a dispersion containing species as a solute and precipitate). NMR augments speciation determination upon addressing Ln(III) and GLU concentration- as well as pH-dependent structural changes from the ligand’s perspective. Additionally, NMR spectral features provide clues on dynamics such as ligand exchange reactions. Relative energies of different complex species (isomers) determined by DF calculations enable a decision to be made as to whether and which species are likely to be formed under certain conditions. So, in addition to examining the coordinating sites, the fundamental question of the origin/site of protons abstracted upon complex formation and/or increasing pH—GLU’s hydroxyl groups or hydrolysis of metal coordinating water—can be addressed systematically and separately. This approach considerably exceeds the results of previous studies.

## Experimental Section

No uncommon hazards are noted. To avoid the formation of carbonates, sample preparation was carried out in gloveboxes under a N_2_ or Ar atmosphere using deionized water (18.2 MΩ cm, Millipore GmbH, Schwalbach, Germany), which had been decarbonated by purging with Ar overnight or boiling for about 40 min, prior to use.

For sample preparation as well as for pH and ionic strength adjustments, the following chemicals were used as obtained: sodium gluconate (meets USP testing specifications), EuCl_3_ (anhydrous, 99.99%), TbCl_3_·6H_2_O (99.999%), and Nd(NO_3_)_3_·6H_2_O (99.9%) (all from Sigma-Aldrich, USA); LaCl_3_·7H_2_O (99.999%), SmCl_3_·6H_2_O (>99%), and LuCl_3_·5H_2_O (>99.99%) (all from Sigma-Aldrich, Germany); 2-bromopropane (Merck, Germany), hydrochloric acid, perchloric acid and carbonate-free sodium hydroxide solution (all from VWR Chemicals BDH, USA), and sodium perchlorate (Fluka Analytical, Germany); D_2_O (99.98% D) and DCl and NaOD (both >99% D) (all from Deutero, Germany).

### Time-Resolved Laser-Induced Luminescence Spectroscopy

For the TRLFS experiments, appropriate amounts of sodium gluconate and Ln(III) stock solutions were mixed with deionized water in PMMA cuvettes (Sarstedt AG & Co. KG, Germany) to yield the desired Ln(III) and GLU concentrations. The pH was adjusted to the desired value using HCl or NaOH (pH electrode and pH meter: Orion Star, Thermo Fisher Scientific Inc., USA). The samples were left to equilibrate for about 5 weeks before the TRLFS measurements to ensure that chemical equilibrium was reached. Samples in which a precipitate was visible or suspected were stirred during TRLFS measurements to move the solid particles into the light path (PTFE stirring rods: Carl Roth GmbH & Co. KG, Germany; stirring unit: electronic stirrer model 300, Rank Brothers, United Kingdom). Generally, Eu(III) was used as the spectroscopic probe in the TRLFS experiments, but some additional experiments with the energy transfer pairs Eu(III)/Nd(III) and Tb(III)/Eu(III) were carried out. The Ln(III) concentration was 1 × 10^–3^ M in order to work in a concentration range comparable to NMR measurements and in order to have the flexibility to adjust different molar ratios of Eu(III):GLU over a large concentration range of GLU. No background electrolyte was added to the samples.

Eu(III) TRLFS measurements were carried out using a 20 Hz Nd:YAG laser (Quanta Ray, Spectra Physics, USA) and OPO (GWU-Lasertechnik, Germany) system. The ^5^L_6_ ← ^7^F_0_ transition of Eu(III) was excited at 394 nm. The emission bands of the ^5^D_0_ → ^7^F_0_ to ^5^D_0_ → ^7^F_4_ transitions in the wavelength range of 575–715 nm were measured with a spectrograph (Shamrock 303i, Andor Technology, Oxford Instruments, United Kingdom) equipped with a 300 l/mm grating (blaze: 500 nm) and an ICCD camera (iStar DH734-18H-13, Andor Technology, Oxford Instruments, United Kingdom). For all samples, the boxcar technique was used with an initial delay of 10 μs, a gate width of 1000 μs, a slit width of 25 μm, and a linear increasing gate step. For the Tb(III) → Eu(III) energy transfer experiments, the Tb(III) ions were excited at 351 nm (because Eu(III) does not show much absorption at this wavelength) and the emission was recorded in the wavelength range of 474–615 nm. All other settings were identical to the Eu(III) experiments.

The TRLFS data were corrected for the wavelength-dependent sensitivity of the ICCD camera and the grating and were deconvoluted using a parallel factor analysis algorithm (PARAFAC^22,23^ executed in MATLAB 2023b^24^) to yield the luminescence spectra, luminescence decay kinetics, and luminescence intensities of each individual Eu(III) species. Based on the deconvoluted luminescence spectra, the Judd–Ofelt parameters, the radiative decay time, and the luminescence quantum yield of each species were calculated. Using the quantum yield ϕ_*i*_ and assuming that the extinction coefficients at 394 nm of all present Eu(III) species are similar, the luminescence intensities *I*_*i*_ of each species *i* obtained from PARAFAC analysis were converted into molar fractions *x*_*i*_ using eq 1
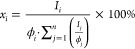
1

### Capillary Electrophoresis-Inductively Coupled Plasma Mass Spectrometry

All capillary electrophoresis (CE) measurements were performed using an Agilent 7100 CE system (Agilent Technologies, Waldbronn, Germany) hyphenated to an Agilent 7900 ICP–MS system (Agilent Technologies, Wiesental, Germany). The coupling was realized via a MiraMist CE Nebulizer (Burgener Research, Mississauga, Canada) and a Scott-type spray chamber (AHS Analysentechnik, Tübingen, Germany). A fused silica capillary (TSP0503753, Polymicro Technologies, Phoenix, Arizona, USA) with 50 μm inner diameter and 50 cm length was used.

Using ICP-MS standards (High-Purity Standards, Inc. (HPS), Charleston, South Carolina, USA) of La, Sm, Eu, Gd, and Lu, a lanthanide cocktail with [Ln] = 1 × 10^–3^ M was prepared. Cs^+^ was added as an internal standard with [Cs] = 1 × 10^–3^ M. For each sample, 5 μL of the cocktail was added to a centrifuge tube and left to evaporate overnight before being transferred into the glovebox.

For each measurement, 10 mL of an appropriate background electrolyte (BGE) with the desired GLU concentration, pH value, and ionic strength was prepared. The ionic strength was fixed at 0.1 M using NaClO_4_ and the pH values were adjusted using HClO_4_ and carbonate-free NaOH (pH meter: inoLab pH 720, Xylem, Weilheim, Germany, equipped with a SI AnalyticsBlueLine 16 pH microelectrode, Mainz, Germany, 3 M NaCl). The Ln(III) cocktail was redissolved in 5 mL of each BGE resulting in [Ln(III)] = 1 × 10^–6^ M. No significant change in pH was observed upon addition of the Ln cocktail. 2-Bromopropane was added to the samples as a neutral marker for the electroosmotic flow and was detected as ^79^Br with ICP-MS. The lanthanides were detected as ^139^La, ^147^Sm, ^153^Eu, ^157^Gd, and ^175^Lu with ICP-MS. All samples were measured immediately after preparation, and selected samples at pH 10 were remeasured after 6 weeks remaining in the glovebox. The determination of complex formation constants by CE is described in detail by Willberger et al.^25^ For the formation of binary Ln(III)–GLU complexes at low pH, the equilibrium in eq 2 was assumed. Corresponding stability constants β_*i*_ were calculated according to eq 3.
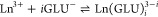
2
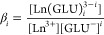
3

Based on the equilibrium in eq 2, eq 4 was drawn up to describe the effective electrophoretic mobility μ_eff_ in relation to the free gluconate concentration [GLU]_free_ as well as the individual mobilities of the species μ_*i*_. To determine [GLU]_free_, the p*K*_a_ value of 3.7 for *I* = 0.1 M NaClO_4_ was used.^26^ The measured electrophoretic mobilities μ_eff_ were plotted against [GLU]_free_, and by fitting eq 4 to the experimental data, complex formation constants log β_*i*_ were obtained.
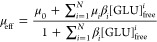
4

All complex formation constants were extrapolated to zero ionic strength using the Davies equation.^27^

### Nuclear Magnetic Resonance Spectroscopy

NMR spectra were recorded at (25 ± 0.2) °C (unless stated otherwise) mainly with an Agilent DD2-600 system and occasionally on an Agilent MR-400 system, operating at 14.1 and 9.4 T, with corresponding ^1^H and ^13^C resonance frequencies of 599.8 and 150.8 or 399.8 and 100.8 MHz, respectively, using 5 mm oneNMR probes.

^1^H NMR spectra were measured by accumulating a varying number of scans depending on concentrations of the individual sample series, using 2 s of acquisition time and relaxation delay, respectively, applying a 2 s presaturation pulse on the water resonance for water signal suppression followed by a π/6 excitation pulse. For ^13^C{^1^H} NMR measurements, 1024 scans were accumulated upon applying 4 s relaxation delay after the π/6 excitation pulse and 1 s acquisition time, with ^1^H broadband decoupling.

In case of potential ambiguity, signal assignment was validated by two-dimensional correlation techniques. Therefore, heteronuclear single-quantum coherence (HSQC) and heteronuclear multiple-bond correlation (HMBC) were accomplished using pulse sequences taking advantage of gradient-selection and adiabatic pulses. HMBC and HSQC spectra were acquired with 2k × 1k complex points in *F*_2_ and *F*_1_, 100 and 64 transitions per *F*_1_ increment, and a relaxation delay of 1 s, respectively. For polarization transfer, (2*J*)^−1^ delays of 100.0 and 3.45 ms were opted, corresponding to 5 Hz ^*n*^*J* in HMBC and 145 Hz ^1^*J* in HSQC, respectively.

La(III), Sm(III), and Lu(III) stock solutions and dilutions thereof were prepared using deionized water and D_2_O (99.95% D, Deutero, Germany). pH was adjusted with HCl (1.0, 0.1, and 0.01 M) and NaOH (1.0, 0.1, and 0.01 M) and in D_2_O solutions, likewise, with DCl and NaOD (99% D, Deutero, Germany), using a pH meter (inoLab pH 730, Xylem, Weilheim, Germany) equipped with a pH electrode (Schott, BlueLine, SI Analytics, Mainz, Germany). The pH meter was calibrated using a three-point calibration procedure. In case of samples at pH 5, buffers at pH 1.679, 4.006, and 6.865 were used, while for samples at pH 10, 11, and 13, standard buffers at pH 6.865, 9.180, and 12.47 were used (standard DIN/NIST buffer solutions, WTW).

pH was corrected for deuterium according to the common pD = pH (read) + 0.4 in pure D_2_O. Since the reading of the pH meter is nearly a linear function of the atom-% deuterium,^28^ the used 10% D_2_O contents needed the addition of 0.04 pH units.

#### pH-Titration Series of GLU Only

^1^H and ^13^C NMR reference spectra were obtained at the NMR-400 from a pH-titration series in the range 1.0 ≤ pH ≤ 13, with increments of 1 pH unit, for aqueous solutions 30 mM in GLU, containing 10% D_2_O.

#### pH-Titration Series of GLU–Ln(III) Systems

Aqueous solutions containing 10% D_2_O were prepared in the range 1.0 ≤ pH ≤ 13 with increments of 1 pH unit, at 1 mM concentrations equimolar in ligand and metal, the latter comprising the trivalent chlorides of La, Lu, and Sm. ^1^H NMR spectra were obtained by accumulating 64–1024 individual spectra, depending on line broadening and/or precipitate formation. In case of the latter, samples were centrifuged and the supernatants were measured. Solutions were measured immediately after preparation and 6 weeks later. Additionally, selected samples were measured at *T* = 0 °C.

In case of pH-titration series (with and without Ln(III)), covering the entire pH range, we used a VWR pHenomenal^(R)^ MU 6100 L pH meter equipped with a WTW SenTix Mic pH electrode, applying a five-point calibration using the five standard buffer solutions mentioned above.

#### Ln(III)-to-GLU Titration Series

Series of aqueous solutions containing 10% D_2_O were prepared at pH 5 (4.90–5.10) and at pH 10 (9.90–10.10). [GLU] was kept constant at 5.0 mM, and the [Ln(III)] (Ln = La, Lu, Sm) was varied between 0.10 and 5.0 mM, respectively. ^1^H NMR spectra were measured (La and Lu at the NMR-600, Sm at the NMR-400) upon averaging 32 (pH 5) or 64 (pH 10) individual spectra. In case of precipitation, i.e., at pH = 10 for GLU:Ln(III) ≥ 5:2, samples were centrifuged and the supernatants were measured.

#### GLU-to-Ln(III) Titration Series

Because of samples containing GLU in the μM range, these solutions were prepared in pure D_2_O, at pD 5 (4.95–5.05), at a constant [Ln(III)] of 1.0 mM. [GLU] was varied between 50 and 900 μM, comprising 7 (La and Sm) or 14 titrations steps (Lu). Depending on ligand concentration, spectra were measured using 32 up to 512 scans.

#### Series Dedicated to Study the Reaction Behavior under (Hyper-)Alkaline Conditions

In D_2_O, solutions 10 mM in either La(III) or Lu(III) were prepared with Ln(III):GLU ratios of 1:1 and 1:3, each at pD 11.0 and 13.0 (±0.10). All samples were prepared in duplicates, one set measured immediately after preparation and the other set after eight months untouched. Prior to measurement, all samples were centrifuged and the clear supernatants were subjected to ^1^H NMR with the number of scans varying between 16 and 256.

### Density Functional Calculations

Density functional (DF) calculations utilizing the PBE functional were carried out for the example of La(III) complexes,^29^ employing the Turbomole program package (version 6_6).^30,31^ Basis sets of triple-ζ quality with polarization functions, def-TZVP, were used for all atoms. For La, the Dolg–Stoll–Savin small core effective core potential (ECP) was employed to account for core electrons and related relativistic effects.^32^ Spin-restricted electronic structure calculations were done due to the closed electronic shell of La^3+^. The resolution of identity (RI) approximation was used to accelerate the self-consistent field procedure during geometry optimizations, thus reducing the computational cost without significantly compromising accuracy.^33^ In the gas phase, all structures were checked to be minima on the potential energy surface by means of a vibrational normal-mode analysis. With the obtained vibrations, thermal free energy corrections were calculated, which were subsequently added to the energies in solution to obtain Gibbs free energies in solution for each species according to a thermodynamic cycle. While explicit water molecules in the complexes account for the short-range solvation effects around the metal center, for the long-range effects of solvation, the COSMO^34^ variant of the polarizable continuum solvation model^35,36^ was used with a dielectric constant of 78.4 and the default water parameterization as implemented in Turbomole. Vibrational frequency calculations were performed also in solution to confirm the structures to be minima on the potential energy surface. Most of them were verified as true minima, while some exhibited one to four low-magnitude imaginary vibrational frequencies corresponding to soft, strongly delocalized modes. Where these could be resolved, the resulting energy improvements remained below 2 kJ/mol.

Initial geometries for all studied complexes were generated from optimized La^3+^ aqua complexes. The coordination number (CN) of these complexes has been optimized by varying the number of aqua ligands. Mono- and dihydroxo La complexes were generated by removal of one and two protons from aqua ligands, respectively, and subsequent optimization. These complexes together with aqua species were used as references when calculating reaction energies. One to three aqua ligands were removed from the La aqua complexes and replaced by gluconate to generate various isomers of the gluconate complexes. For each of these complexes, the CN around the La ion was carefully checked, and for the thermodynamic considerations, only the most stable variant was retained.

## Results

### Ln(III)–GLU Complexation at pH 4

Ln(III)–GLU complexation was investigated in the pH range of 4–13. Based on thermodynamic data,^37−39^ up to pH 6 and in absence of GLU, hydrolysis of Ln(III) can be neglected, and the respective aquo ion is the only metal species present in solution. Concerning GLU, the carboxyl but none of the hydroxyls undergoes deprotonation. Lactonization is relevant only for pH < 4. Only the formation of binary Ln(III)–GLU complexes of 1:*n* (*n* = 1–4) stoichiometry was observed.

### TRLFS Study at pH 4

**Figure 2:**
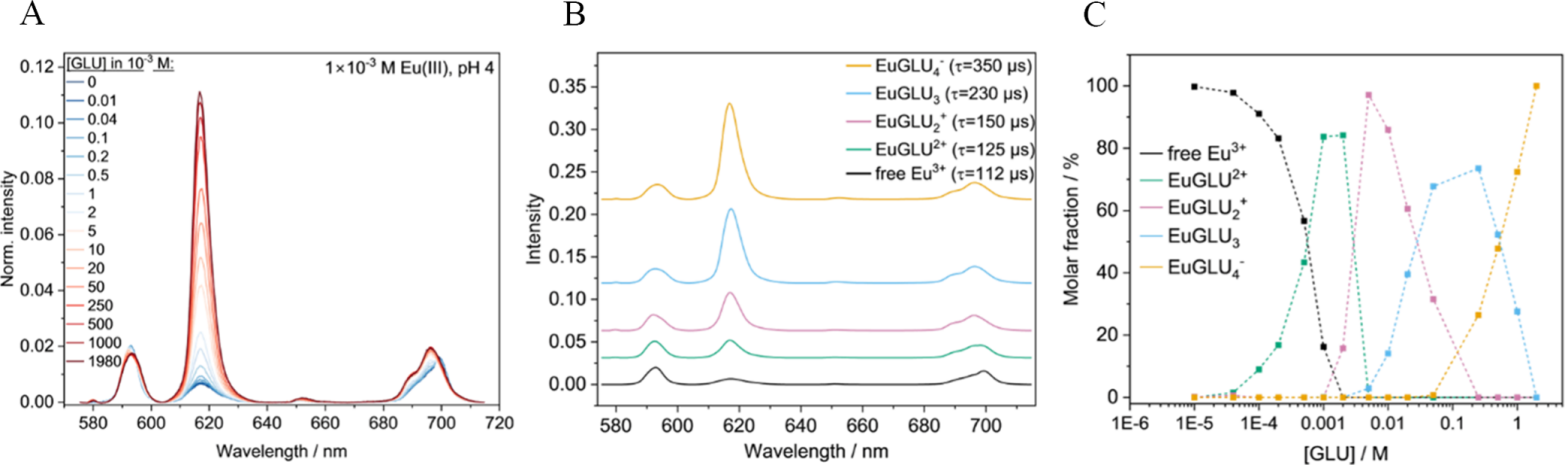
Luminescence spectra, normalized to the ^5^D_0_→^7^F_1_ peak area, of the Eu–GLU samples with 1 × 10^–3^ M Eu(III) at pH 4 as a function of [GLU] after a gate delay of 10 μs (A), deconvoluted luminescence spectra of the individual Eu species that were obtained using PARAFAC (B), and speciation diagram based on PARAFAC analysis (C).

Figure 2A shows the normalized luminescence spectra of the Eu(III) samples (1 × 10^–3^ M Eu(III)) at different GLU concentrations at pH 4. The complexation with GLU is indicated by the spectral differences upon the addition of GLU, mainly in the intensity of the hypersensitive ^5^D_0_ → ^7^F_2_ transition of Eu(III) at 618 nm (Figure 2). Deconvolution of the TRLFS data with PARAFAC gives rise to the Eu(III) aquo ion and four different Eu–GLU complexes. The deconvoluted luminescence spectra, the respective luminescence decay times, and the speciation diagram in dependence on the GLU concentration are presented in Figure 2B,C, respectively. The fourth complex species was observed only at GLU concentrations above 1 × 10^–1^ M.

For a first tentative interpretation in terms of stoichiometry of the Eu–GLU complexes, the luminescence decay times were considered. The Eu(III) luminescence is quenched by the presence of water due to an energy transfer from the ^5^D_0_ level to an overtone of the O–H stretching vibration. Hence, the number of water molecules, n(H_2_O), in the first coordination sphere of the Eu(III) ion can be estimated from the luminescence decay time τ according to an empirical equation, eq 5 by Marmodée et al.^40^
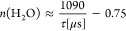
5

The estimated number of coordinating water molecules for each Eu–GLU complex is shown in Table 1. It is tempting to assume that the coordination of one GLU molecule displaces one to two water molecules, which correlates well with previous findings that complexation at low pH mainly occurs via coordination of the carboxyl group (cf. Sections Ln(III)–GLU Structure Study by NMR and Ln(III)–GLU Structure Study by DF Calculations).^18,19,21^ We would like to stress that the calculated number of water molecules in the first coordination sphere is just an estimate as τ might also be influenced by an additional energy transfer to the GLU hydroxo groups and by other factors.

**Table 1:** Luminescence Decay Time τ and Estimated Number of Water Molecules *n*(H_2_O) in the First Coordination Sphere of Free Eu^3+^ and the Eu–GLU Complexes at pH 4

complex	τ/μs	*n*(H_2_O)
Eu^3+^	112 ± 3	8–9^a^
EuGLU^2+^	125 ± 3	8
EuGLU_2_^+^	150 ± 10	6–7
EuGLU_3_	230 ± 10	4
EuGLU_4_^–^	350 ± 10	2

a It was taken into account that in an aqueous solution there is an equilibrium between 8 and 9 coordinating water molecules. Based on the decay time and eq 5, a value of 8.9 is calculated.^40^

According to the first tentative analysis, Eu–GLU complexes with one to four GLU molecules form, depending on the Eu:GLU ratio given in solution. Figure S1, Supporting Information, shows the alteration of the calculated number of water molecules in the first coordination sphere with increasing the number of GLU ligands. From the fit, a decent correlation with a slope of −2 was obtained indicating that on average, two water molecules are displaced from the first coordination sphere per GLU ligand molecule. The correlation further indicates no quenching due to GLU being present at pH 4.

### CE-ICP-MS Study at pH 4

**Figure 3:**
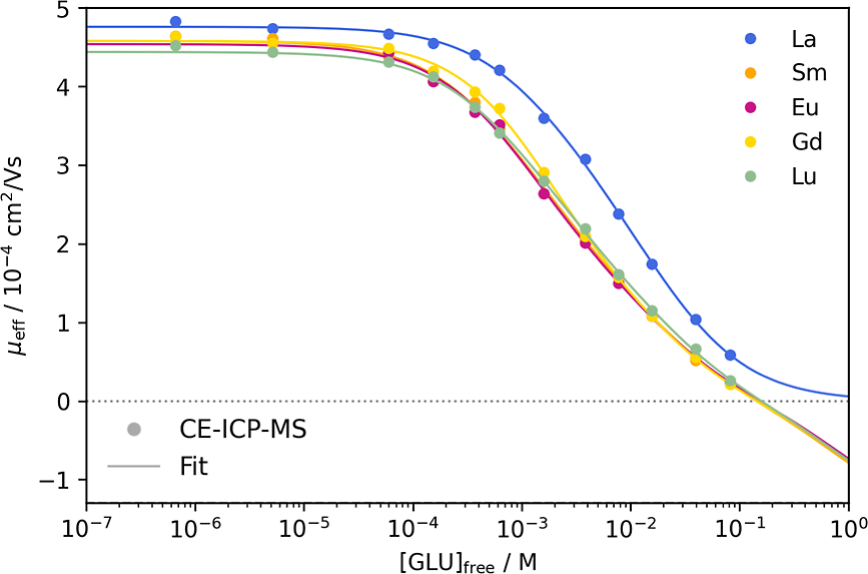
Plot of the measured electrophoretic mobilities μ_eff_ of La, Sm, Eu, Gd, and Lu (1 × 10^–6^ M each) against free gluconate concentration [GLU]_free_ at pH 4 and *I* = 0.1 M (NaClO_4_). Fits include 1:1, 1:2, 1:3, and 1:4 Ln–GLU complexes using eq 4.

Complementary to the TRLFS experiments, the complexation of Ln(III) ions such as La(III), Sm(III), Eu(III), Gd(III), and Lu(III) by GLU was investigated. The electropherograms as well as the electrophoretic mobilities are summarized in Figure S9 and Table S2, Supporting Information. The measured electrophoretic mobilities of 1 μM Ln(III) at pH 4 and for various GLU concentrations are shown in Figure 3.

Figure 3 shows a decrease in electrophoretic mobility with an increase in free gluconate concentration. Since hydrolysis can be neglected at pH 4, the decrease in mobility indicates a successive association of GLU ligands to the Ln(III) ions, decreasing the mean charge in solution. The fits shown in Figure 3 were obtained using eq 4 for the formation of four Ln(III)–GLU complexes. All coefficients of determination were *R*^2^ ≥ 0.998. The electrophoretic mobility μ_*i*_ for each species can be found in Table S1, Supporting Information. The therefrom calculated complex formation constants log β_*i*_ are summarized in Table 2.

**Table 2:** Complex Formation Constants log β_*i*_, of LnGLU_*i*_^3–*i*^ Obtained from the Fitting Procedure as well as log β_*i*_ Determined by Giroux et al.,^21^ Both at *I* = 0.1 M (NaClO_4_) and *T* = 25 °C

	La(III)	Sm(III)	Eu(III)	Gd(III)	Lu(III)	refs
log β_1_	2.90 ± 0.04	3.36 ± 0.05	3.33 ± 0.06	3.14 ± 0.04	3.27 ± 0.04	p.w.^a^
	2.91 ± 0.02		2.82 ± 0.05		3.58 ± 0.03	21
log β_2_	4.97 ± 0.04	5.97 ± 0.04	5.97 ± 0.05	5.72 ± 0.04	5.77 ± 0.04	p.w.
	4.85 ± 0.08		5.74 ± 0.04		6.41 ± 0.03	(21)
log β_3_	6.41 ± 0.04	7.63 ± 0.06	7.62 ± 0.07	7.42 ± 0.05	7.35 ± 0.05	p.w.
log β_4_		7.68 ± 0.27	7.63 ± 0.35	7.43 ± 0.24	7.40 ± 0.23	p.w.
log *K*_4_		0.05 ± 0.28	0.01 ± 0.36	0.02 ± 0.25	0.05 ± 0.24	p.w.

a p.w. = present work.

Sm(III), Eu(III), Gd(III), and Lu(III) show similar log β values, whereas those of La(III) are smaller. La(III), having the lowest charge density, shows the lowest log β values compared to the other lanthanides. The Ln(GLU)_4_^–^ complex is not present to a significant extent in the concentration range used in the CE-ICP-MS experiments. Nevertheless, the 1:4 complex was included in the fitting model according to eq 4 of the measured mobilities, allowing estimation of the complex formation constant log β_4_. For La(III), the log β_4_ value obtained by the fit was nearly 0, making the formation of La(GLU)_4_^–^ seem unlikely. Should a potential LaGLU_4_^–^ complex form, then only at significantly higher [GLU]. For Sm(III), Eu(III), Gd(III), and Lu(III) in contrast, the fit resulted in nonzero complex formation constants for the Ln(GLU)_4_^–^ complexes. The differences between the log β_4_ and log β_3_ values, i.e., log *K*_4_, are very small indicating the association of a fourth GLU ligand to the lanthanide ion only taking place at very high gluconate excess.

The log β values for the 1:1 and 1:2 complexes of La(III) obtained in this work are in good agreement with the ones obtained by Giroux et al.^21^ For Eu(III), log β_1_ obtained in this work is higher compared to Giroux et al.,^21^ while log β_2_ is again in agreement. Deviations might result from the different experimental setups. Our ligand to metal ratio (L:M) was varied over several orders of magnitude while their ligand concentration was about 5–8 mM and the L/M was varied only from 1:1 to 5:1.^21^ At [Ln(III)] = 1 × 10^–6^ M, no stoichiometric limiting of the Ln(III) ions is expected at low GLU concentrations. The log β values of Lu in this work are somewhat lower compared to those reported.^21^

### Ln(III)–GLU Complexation at pH 10 and 12

The hydrolysis of Ln(III) has a great influence on the speciation at higher pH values. In contrast to pH 4, the samples at pH ≥ 10 also included precipitated species, which influenced the applicability of the analytical methods to a different extent.

### TRLFS Study at pH 10

**Figure 4:**
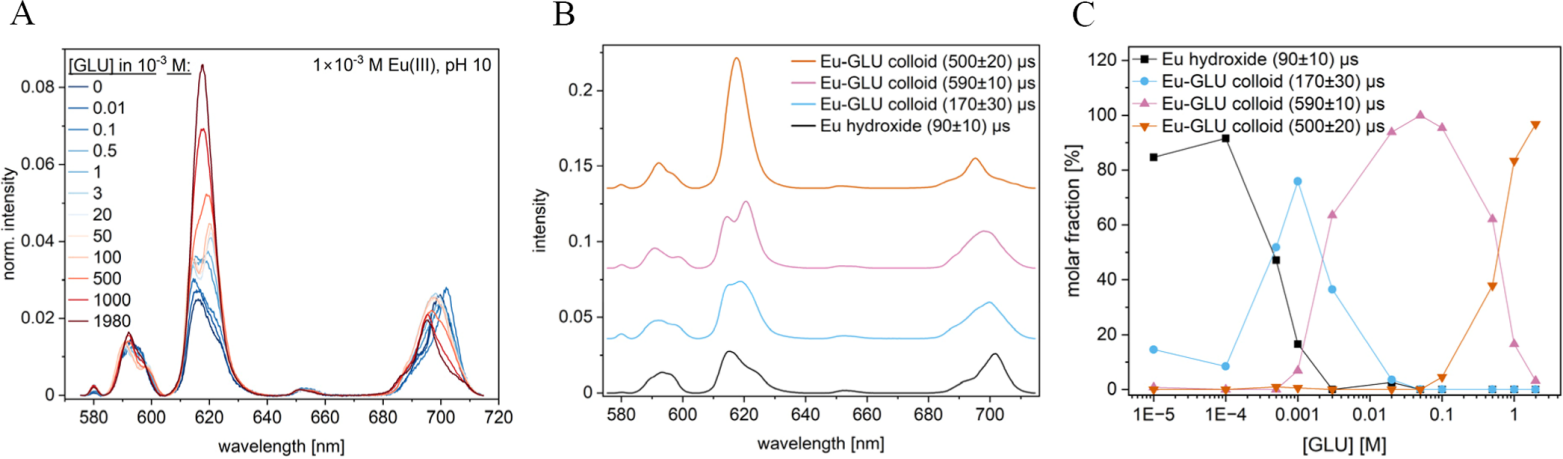
Luminescence spectra, normalized to the ^5^D_0_→^7^F_1_ peak area, of the Eu–GLU samples with 1 × 10^–3^ M Eu(III) at pH 10 as a function of [GLU] (A), deconvoluted luminescence spectra of the individual species (B), and speciation diagram based on PARAFAC analysis (C).

Using the TRLFS setup, emission of solids as well as of solutions can be collected. At pH 10, a white precipitate was observed in the samples with 1 × 10^–3^ M Eu(III) and ≤1 × 10^–3^ M GLU. Above 1 × 10^–3^ M GLU, no precipitate was visible by the naked eye. To measure the luminescence of Eu(III) (precipitated as well as solvated species), the samples were stirred during the TRLFS measurements. Deconvolution of the luminescence data using PARAFAC yielded four species, which correspond to precipitated Eu(OH)_3_(am) with a τ of (90 ± 10) μs^41^ and three Eu–GLU species with a τ of (170 ± 30) μs, (590 ± 10) μs, and (500 ± 20) μs, corresponding to approximately 6, 1, and 1–2 water molecules, respectively (cf. Figure 4). The spectroscopic parameters did not match with the Eu–GLU complexes observed at pH 4 (vide supra).

The spectroscopic differences (spectral intensity distribution, τ values) indicate that the ligand field of Eu(III) at pH 10 is different from that at pH 4, which could be due to, e.g., deprotonation of the hydroxyl groups or the formation of ternary Eu(OH)_*x*_GLU_*y*_ complexes.

A combination of solution and precipitation species contribute to the overall TRLFS signals. In order to further discriminate between the two fractions, additional experiments were carried out: (i) mixed Ln(III) samples for resonance energy transfer (RET) investigations and (ii) ultracentrifugation to separate soluble species from the precipitate.

### Resonance Energy Transfer (RET) Experiments for Characterization of the Solid Phase

RET is frequently used in analytical and environmental sciences, e.g., for the determination of binding as well as distances between molecules/ions on a molecular level, or in so-called upconversion nanoparticles to shift the excitation wavelength into the biological window.^42−46^ RET pairs of Ln(III) were selected with an energy donor (D) and an acceptor (A), viz., D1 = Eu(III), A1 = Nd(III) and D2 = Tb(III), A2 = Eu(III), varying the D:A ratio at a total Ln(III) concentration of 1 × 10^–3^ M for a GLU concentration of 1 × 10^–3^ M at pH 10. In the Eu(III)/Nd(III) pair, the Nd(III) completely quenched the Eu(III) luminescence of the species with a τ = (170 ± 30) μs, which is the dominant species under these conditions (cf. Figure S2A, Supporting Information). For the Tb(III)/Eu(III) pair, the sample was excited at λ = 351 nm. Under the experimental conditions, at this wavelength, Eu(III) is only weakly excited directly while Tb(III) is sufficiently excited. Due to RET in the mixed sample, a strong Eu(III) luminescence and only a weak (quenched) Tb(III) signal were detected as compared to the pure Tb(III) reference sample, as shown in Figure S2B, Supporting Information.

These findings are in line with the expected energy transfer of Eu(III) → Nd(III) and Tb(III) → Eu(III). Since RET is only possible for short D–A distances (≤10 Å for Ln(III) ions), the dominant Eu–GLU species must be either a solvated binuclear complex or an aggregate, which precipitates. In the case of a binuclear complex, both the formation of homonuclear (Eu(III) + Eu(III)) and heteronuclear (Eu(III) + Nd(III) or Eu(III) + Tb(III)) complexes is anticipated, expecting to observe an energy transfer only for the heteronuclear complex but not for the homonuclear Eu(III) + Eu(III) complex. However, in the Eu(III)/Nd(III) samples, complete quenching of the luminescence was observed and no luminescence from a potential homonuclear Eu(III) + Eu(III) complex was detectable, ruling out a binuclear complex (unless the formation of the heteronuclear Eu(III) + Nd(III) complex would be strongly favored over the homonuclear Eu(III) + Eu(III) complex, which is unlikely considering the similar chemical behavior of both lanthanides).^47^ Similar energy transfer experiments at 1 × 10^–1^ M GLU where the Eu–GLU species with τ = (590 ± 10) μs is the dominant species, and at 1.98 M GLU where the Eu–GLU species with τ = (500 ± 20) μs is dominant, showed the same trends as at 1 × 10^–3^ M GLU (cf. Figures S3 and S4, Supporting Information), suggesting that these species as well are aggregates, which precipitate over time. It indicates that over the entire investigated GLU concentration range up to 1.98 M, mainly Eu–GLU aggregates are present in the sample at pH 10.

### Supernatant after Ultracentrifugation

To further investigate the presence of aggregated Eu–GLU species, a sample with 1 × 10^–3^ M Eu(III) and 2 × 10^–2^ M GLU at pH 10 was split in half, one of which being ultracentrifuged and the luminescence signal was compared to the native reference sample, which was not centrifuged. As shown in Figure S8, Supporting Information, 12 days after ultracentrifugation, the spectral signature and luminescence decay kinetics of the ultracentrifuged sample differed significantly from the reference sample. The overall luminescence intensity was lower in the ultracentrifuged sample, suggesting the presence of aggregates in the reference sample, which were removed by ultracentrifugation. In the reference sample, the dominant species appeared to be the species with τ = (590 ± 10) μs (as described above) at all measurement days. In contrast, in the ultracentrifuged sample, the species with τ = (170 ± 30) μs was predominant 12 days after ultracentrifugation and a combination of both species was present 36 days after ultracentrifugation. The RET experiments indicated that both species can be attributed to aggregates, suggesting that either not all aggregates were removed by the ultracentrifugation or that after ultracentrifugation, aggregates were formed again. This appears to be a rather slow process as changes in the speciation of the ultracentrifuged sample still occurred several weeks after ultracentrifugation.

### TRLFS Study at pH 12

**Figure 5:**
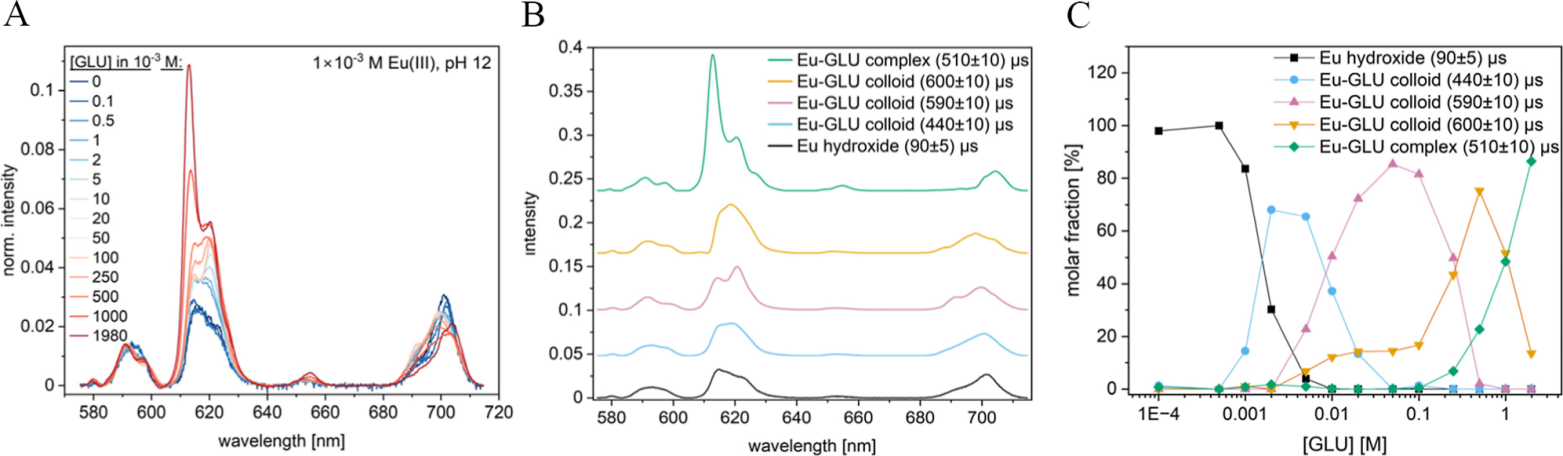
Luminescence spectra, normalized to the ^5^D_0_→^7^F_1_ peak area, of the Eu–GLU samples with 1 × 10^–3^ M Eu(III) at pH 12 as a function of [GLU] (A), deconvoluted luminescence spectra of the individual species (B), and speciation diagram based on PARAFAC analysis (C).

At pH 12 and 1 × 10^–3^ M Eu(III), a white precipitate was visible at [GLU] ≤ 2 × 10^–3^ M. Deconvolution with PARAFAC revealed that this precipitate is likely Eu(OH)_3_(am) as inferred from the characteristic emission spectrum and corresponding τ of (90 ± 5) μs. Additionally, four different Eu–GLU species were observed at higher [GLU] with τ values of (440 ± 10), (590 ± 10), (600 ± 10), and (510 ± 10) μs, respectively. The corresponding luminescence spectra are shown in Figure 5. Energy transfer experiments with the transfer pairs Eu(III) → Nd(III) and Tb(III) → Eu(III) (as described in Section TRLFS Study at pH 10) revealed that the three species with decay times of (440 ± 10) μs, (590 ± 10) μs, and (600 ± 10) μs are most likely aggregates. In contrast, for the species of τ = (510 ± 10) μs, that appeared at GLU concentrations above 2 × 10^–1^ M, no Eu(III) → Nd(III) or Tb(III) → Eu(III) energy transfer was observed (see Figures S5–S7, Supporting Information), suggesting that this species might be a solvated complex or an aggregate with Ln(III)–Ln(III) distances that are too large for RET to occur.

The species of τ = (590 ± 10) μs was also present at pH 10 in the same GLU concentration range (cf. Section TRLFS Study at pH 10). Corresponding to the pH 12 species of τ = (440 ± 10) μs, a species with an identical luminescence spectrum but a significantly shorter τ of (170 ± 30) μs was observed at pH 10. Since the Eu(III) luminescence is quenched by nearby O–H oscillators, this might indicate a similar coordination environment around the Eu(III) ion at both pH values in this GLU concentration range, but with a lower number of O–H oscillators at pH 12, e.g., due to deprotonation of a GLU hydroxyl group or due to exchange of a coordinating water molecule by an OH^–^ ligand. However, in the aggregates, multiple Eu(III) ions are in close proximity to each other and potentially an energy transfer between Eu(III) ions might also occur and hence reduce the luminescence decay time. Therefore, an alternative explanation for the higher decay time at pH 12 might be a larger Eu(III)–Eu(III) distance compared to pH 10. The two species that appeared at [GLU] > 1 × 10^–1^ M were not observed at pH 10. They might have a higher degree of deprotonation of the GLU hydroxyl groups or a larger number of OH^–^ ligands than the species at pH 10 found at equimolar GLU concentration.

### CE-ICP-MS Study at pH 10 and 12

Complementary to TRLFS, but at significantly lower Ln(III) concentrations, CE-ICP-MS was applied as an analytical method. The calculation of electrophoretic mobilities at pH 10 and pH 12 proved to be difficult. In fresh samples at low [GLU] (≤1 × 10^–4^ M), no clear peaks were detected. Even if no precipitation could be observed by the naked eye, the loss of signal can be attributed to precipitation, sorption on the sample vials, or aggregate formation. All investigated Ln(III) showed similar behavior, and the addition of gluconate increased the solubility of the lanthanides. For fresh samples at [GLU] exceeding 1 × 10^–3^ M, clear signals were observed for all investigated Ln(III).

The electropherograms as well as the electrophoretic mobilities are summarized in Figures S10, S11, Tables S3 and S4, Supporting Information. At [Glu] > 1 × 10^–3^ M, all investigated Ln(III) showed a negative electrophoretic mobility around −1 × 10^–4^ cm^2^/(V s) for pH 10 and −2 × 10^–4^ cm^2^/(V s) for pH 12 corresponding to one or more negatively charged complexes. Negatively charged Ln(III)–GLU complexes at elevated pH suggest deprotonation of GLU’s hydroxyl groups (denoted as GLUH_–*j*_) or the formation of ternary Ln(III)(OH)_*x*_GLU_*y*_ complexes at higher pH values. Both possibilities are taken into account by the nomenclature Ln(GLU)_*i*_H_–*j*_^3–*i*–*j*^ according to the equilibrium in eq 6.
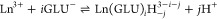
6
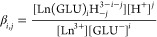
7

Between 1 × 10^–3^ and 0.1 M GLU, no significant change in electrophoretic mobility was observed, indicating no changes in speciation.

To calculate complex formation constants β_*i*,*j*_ of the potential Ln(GLU)_*i*_H_–*j*_^3–*i*–*j*^ complexes (eq 7), a change in electrophoretic mobility is needed. Only La(III), Eu(III), and Lu(III) showed a trend in mobility at pH 10, which can be used for the calculations. At pH 12, none of the Ln(III) showed a useful trend; however, at least the existence of negatively charged (dianionic) complex species is evident.

**Figure 6:**
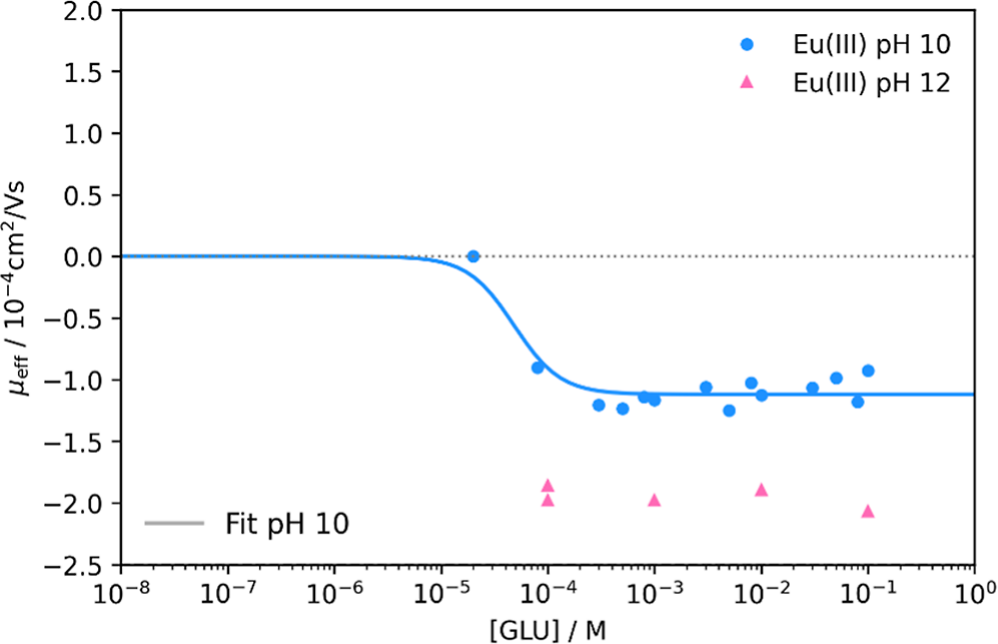
Plot of the measured electrophoretic mobilities μ_eff_ of Eu (1 × 10^–6^ M) against [GLU] at pH 10 and pH 12 and *I* = 0.1 M (NaClO_4_). Fitted for the [Eu(GLU)_2_H_–2_]^−^ complex using eq 10.

Figure 6 shows the electrophoretic mobility of 1 × 10^–6^ M Eu(III) as a function of the gluconate concentration at pH 10 and pH 12. At higher pH values, the hydrolysis of Eu(III) has a significant influence on the speciation, with Eu(OH)_3_ being predominant at both pH values.^37^ For the GLU concentration range where no signals were obtained, a nonsignificant complexation with GLU and therefore the dominance of the hydroxo complex are assumed. At higher [GLU], due to the different measured electrophoretic mobilities of Eu(III) at pH 10 and pH 12, it can be assumed that complexes with different degrees of deprotonation are formed. At pH 10, the electrophoretic mobility indicates a mean charge of about −1 for the complexes, and the assumption of a predominant EuGLU_2_H_–2_^–^ complex best reflects the measured values. The preceding EuGLUH_–2_ complex is charge-neutral and cannot be differentiated from Eu(OH)_3_ using CE-ICP-MS. EuGLUH_–3_^–^ would also match the charge, but the sharp decrease in mobility between 10^–5^ and 10^–4^ M GLU indicates a dependency on multiple GLU ligands. Stoichiometries corresponding to more than two GLU also seem unlikely compared to the results at pH 4. So, only EuGLU_2_H_–2_^–^ was considered for the fit based on the equilibrium in eq 8 with a corresponding formation constant *K*_2,2_ according to eq 9
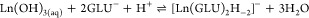
8
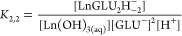
9

Based on this equilibrium, eq 4 was adapted to yield eq 10
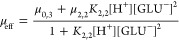
10

Formation of Ln(OH)_3(aq)_ upon Ln(III) hydrolysis and its corresponding formation constant β_0,3_ can be described by eqs 11 and 12, respectively.
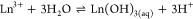
11
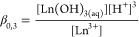
12

The estimated electrophoretic mobility for the Eu(OH)_3_ complex, μ_0,3_, is zero and the limiting electrophoretic mobility of the plateau at high [GLU] was selected for μ_2,2_ of the Eu(GLU)_2_H_–2_^–^ complex. The complex formation constants of La(III) and Lu(III) were calculated analogously (Figure S13, Supporting Information).

As summarized in Table 3, all three Ln(III) show similar complex formation constants within the experimental uncertainties with a slight increase from La(III) to Eu(III). The cumulative complex formation constants log β_2,2_ of the LnGLU_2_H_–2_^–^ complexes, based on the equilibrium in eq 8, were calculated as the sum of our determined log *K*_2,2_ and the log β_0,3_ reported for Ln(OH)_3(aq)_. For the latter, no general agreement on the value of the complex formation constants can be found in the literature. For Eu(OH)_3(aq)_, the value proposed in the most recent review by Jordan et al. was chosen and extrapolated to *I* = 0.1 M (Table 3).^37^ For La(III) and Lu(III), the values estimated by Lee and Byrne based on the linear free-energy relationships of the lanthanides were selected.^38^ Lee and Byrne also estimated a value for Eu(III) which is also included for the calculations in Table 3.^38^ The trend in log β_2,2_ values originates mainly from the trend in hydrolysis constants yet showing an increase in complexation strength from La(III) to Lu(III) as expected from the increase in charge density toward Lu(III).

**Table 3:** Calculated Complex Formation Constants log *K*_2,2_ and log β_2,2_ of Ln(GLU)_2_H_–2_^–^ at *I* = 0.1 M (NaClO_4_) and *T* = 25 °C, Obtained from the Fitting Procedure as well as of Ln(OH)_3_ log β_0,3_ Literature Data Recalculated for *I* = 0.1 M Using the Davies Equation^27,37,38^

	La(III)	Eu(III)	Lu(III)	refs
log *K*_2,2_	18.47 ± 0.27	18.63 ± 0.19	18.64 ± 0.24	p.w.^a^
log β_0,3_	–28.54 ± 1.03	–26.05 ± 0.39	–24.49 ± 1.18	(38)
		–24.81 ± 0.10		(37)
log β_2,2_ = log *K*_2,2_ + log β_0,3_	–10.07 ± 1.06	–7.41 ± 0.43	–5.85 ± 1.20	p.w.,^38^
		–6.18 ± 0.21		p.w.,^37^

a p.w. = present work.

The electrophoretic mobilities at pH 12 and high GLU excess indicate the presence of a complex with a charge of −2. Under the previous considerations at pH 10, with the only difference between the two series being the increased pH, this complex can be best described as EuGLU_2_H_–3_^2–^.

Selected samples at pH 10 were remeasured after 6 weeks and did not show any signals typically observed for solvated species in CE-ICP-MS except for Lu(III) in some samples. Instead, very sharp signals corresponding to aggregates entering the ICP-MS were observed (see Figure S11, Supporting Information). This indicates that the kinetics of the aggregate formation are slow and that also at [Ln(III)] as low as 1 × 10^–6^ M, eventually precipitation occurs.

### Ln(III)–GLU Structure Study by NMR

#### pH-Titration Series of GLU Only

In the acidic pH range, GLU’s carboxylic group is deprotonated causing signal shifts being upfield in ^1^H and downfield in ^13^C NMR (cf. Figure S14, Supporting Information); reported p*K*_a_ values are between 3.2 and 3.9 depending on the background electrolyte and concentration.^48^ Additionally, acid-catalyzed formation of the corresponding glucono γ- (pH 1.1) and δ-lactones (pH 1.1 through 3.1) is observed in the spectra obtained in acid media (cf. Figure S15, Supporting Information).^49^ In the pH range of 6–12, ^1^H and ^13^C NMR spectra of aqueous GLU solutions are virtually identical as there is no alteration in the molecular structure (speciation). At pH 13, (commencing) proton abstraction is observed at C4–OH as concluded from the largest (positive) ^13^C signal shift observed for C4 and supported by the rather strong shift for the C1 signal (γ-effect). All ^1^H signals show negative shifts, not only attributed to the additional negative charge but also caused by altering intramolecular hydrogen bonds along with the molecule’s conformation. The smallest shift observed for H2 rules out deprotonation at C2–OH (cf. Figure S16, Supporting Information). Considering that up to pH 12, the spectra reveal no speciation change and that (de)protonation-induced shifts are detectable at least ± 1.5 pH units around the corresponding p*K*_a_, the latter value for GLU’s second H^+^ abstraction must be close to 14, in line with Kutus et al.^50^ and in fair agreement with Zhang et al.^49^ suggesting a value of 13 ± 1. However, as will be of importance in the complexation studies, H^+^ abstraction at any site, not necessarily being C4–OH, can be facilitated at much lower pH (corresponding to much lower p*K*_a_) upon metal ion coordination via metal-ion-promoted ligand deprotonation.^48,51,52^

#### Ln(III)-to-GLU Titration Series (pH 5)

**Figure 7:**
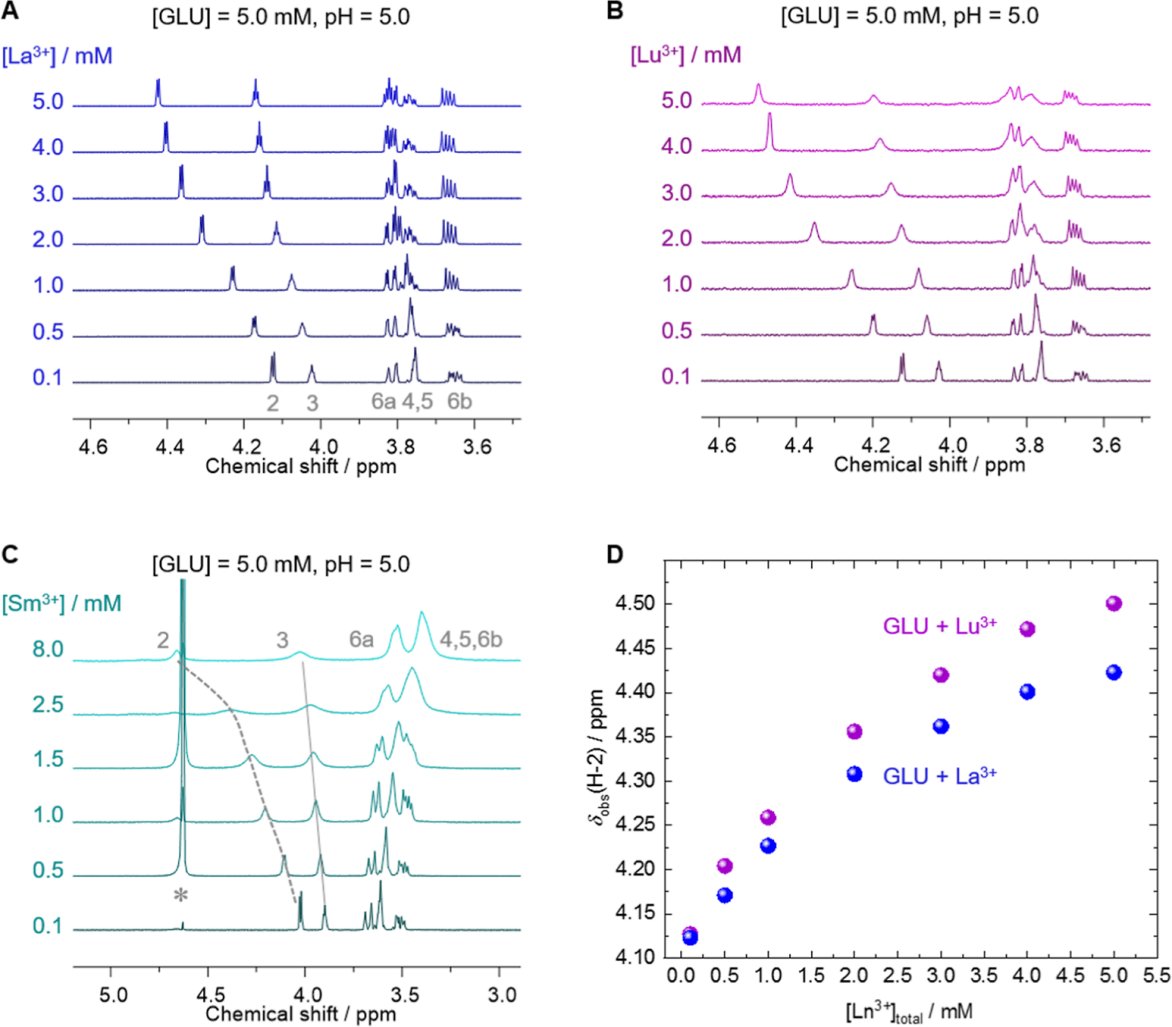
^1^H NMR spectra obtained from 5 mM gluconic acid in aqueous solution containing 10% (v/v) D_2_O at pH 5 in the presence of varying concentrations of La^3+^ (A), Lu^3+^ (B), or Sm^3+^ (C) as stated with the spectra. The plot (D) displays the observed ^1^H NMR chemical shift of H2 in dependence on the Ln^3+^ concentration (Ln = La, Lu). For better visualization, the spectra only show regions of interest. The asterisk in (C) indicates the residual water signal at 4.65 ppm.

Owing to ligand exchange reactions between free and bound ligands (and/or different complex species) being fast on the NMR time scale, the observed signal position is a mole fraction-weighted average. That is, upon increasing Ln(III) concentration, the signals increasingly represent ligands bound in complexes (Figure 7). According to speciation calculation (Figure S18, Supporting Information), at the given pH, overall concentrations, and Ln(III) to GLU ratios, the samples comprise besides the free ligand also Ln(III)–GLU complexes of 1:2 and 1:1 stoichiometry, respectively, predominating at the beginning and at the end of the titration series. Diamagnetic La(III) and Lu(III) cause exclusively positive shifts because of Coulomb interaction, paramagnetic Sm(III) reveals additional effects (cf. Figure 7C). Angular and distance-dependent pseudocontact shift and enhanced relaxation, for instance, render a quantitative comparison to the diamagnetic Ln(III) systems difficult. However, qualitatively, the same binding motif is evinced.

Without doubt, the carboxyl group is part of the binding motif. Since Ln(III)-induced signal shifts are the strongest for GLU H2 and attenuate with increasing distance from the binding site, it is concluded that binding via chelation by carboxylate (C1) and the adjacent OH group (at C2) is facilitated, with the hydroxyl group likely being protonated. Ceteris paribus, shifts induced by Lu(III) are stronger as for La(III), in agreement with stronger complexation of Lu(III) due to the higher Lewis acidity (smaller ionic radius hence larger charge density). Analogous series studied at pH 10 (^1^H NMR spectra given in Figure S17, Supporting Information) and 5 mM GLU are characterized by precipitate formation (vide infra) as of [Ln(III)] exceeding 2 mM. Spectral changes are subtle but visible: even in case of precipitation and thus Ln(III) removal from the solution, all three Ln systems reveal increasing signal broadening upon increasing initial [Ln(III)]. In case of Lu(III), also some slight signal shifts of 17 and 6 ppb are detectable for H2 and H3, respectively.

#### GLU-to-Ln(III) Titration Series

Comparable to the setup of TRLFS titration, series of Ln(III) (Ln = La, Sm, Lu) concentrations fixed at 1 × 10^–3^ M and constant pH 5 were prepared for [GLU] varying between 5 × 10^–5^ and 9 × 10^–4^ M. Since these systems contain excess of the free metal ion, among the possible Ln(III)-GLU complexes, the 1:1 complex is by far the favored one while the 1:2 complex reaches only low percentage upon approaching equimolar sample composition. Correspondingly, upon titrating GLU, the concentration of the free ligand increases and the apparent signals are progressively weighted by unbound GLU. Spectra and corresponding chemical shift plots juxtaposed to distribution of free and bound GLU species obtained from CE-ICP-MS analyses are provided in Figure S20, Supporting Information.

#### pH-Titration Series of GLU–Ln(III) Systems

Solutions 1 mM in GLU and 1 mM in Ln(III) were measured for pH 1 through 13 (spectra and chemical shift plots for La(III), Sm(III), and Lu(III) are provided in Figures S22–S25, Supporting Information). To evaluate the complexation-induced effects, spectra of a Ln(III) containing sample were compared to the corresponding blank at given pH. Up to a near-neutral medium, the Ln(III)-induced shifts increase, observing strongest effects always at H2. Above a certain pH, viz., pH ∼ 7 for La and Sm, and pH ∼ 6 for Lu, likely coinciding with the individual Ln(III)’s associated Lewis acidity, NMR spectral behavior changes drastically, comprising both significant broadening due to an increase of coexisting and interconverting species and a remarkable drop in induced shifts associated with fundamental changes in the nature of the complexes. For the solutions containing La(III) at pH 8 as well as Sm(III) at pH 7, owing to extreme broadening caused by ligand exchange between several coexisting species, ^1^H signals of GLU H2 (and H3) are not detectable. This multitude of species is best seen for Lu(III)’s pH 6 sample. These findings mirror the increase of kinetic stability of GLU complexes upon increasing Lewis acidity along the Ln(III) series. Considering the large spectral effect around pH 7, we assume a structural change to occur within the ligand, i.e., a coordinating GLU hydroxyl group (most likely C2–OH) to be deprotonating rather than abstracting a proton from a coordinating water ligand. Although the solutions beyond neutral pH showed turbidity and precipitation, for all Ln(III), the resulting spectra are distinct from those of the Ln(III)-free blanks. Two main observations are noteworthy. (i) In contrast to the spectra obtained up to circumneutral pH, now all signals show displacements of comparable magnitude. (ii) For a given Ln(III), the sample spectra are virtually identical between pH 10 (La) as well as pH 9 (Sm and Lu) and pH 12, indicating a more or less invariable speciation in that pH range. At pH > 7, the magnitude of the downfield shifts is remarkably smaller but now more uniform among all GLU ^1^H signals. We therefore conclude that (i) the net charge of the complexes becomes less positive/more negative, corresponding to less deshielding effects since the metal ions’ positive charge is progressively compensated by more negative sites within the ligand; (ii) further GLU hydroxyl groups deprotonate, likely yielding coexisting isomeric complexes with hydroxyl deprotonation in variable positions as the spectra appear to be averages among interconverting species.

#### Series Dedicated to Study the Reaction Behavior under (Hyper-)Alkaline Conditions

Solutions of 10 mM of either La(III) or Lu(III) were prepared with Ln(III):GLU ratios of 10:10 and 10:30. Those samples being initially adjusted to pD 13, regardless of composition, revealed no alterations in pD after eight months, although all La(III) and Lu(III) samples showed precipitate formation except the 10:30 Lu-containing sample. In contrast, in samples initially being pD 11, pD remarkably shifted to lower values, depending on composition. That is, for solutions of Ln(III):GLU ratios of 10:10 and 10:30, pD values measured after eight months were 7.9 and 8.2 in case of La but 10.5 and 9.6 in case of Lu, respectively. Of these, the La(III) samples showed precipitation, whereas the Lu(III) samples remained clear. These observations imply that for sufficiently high pH (around 13), any deprotonation reactions (be it deprotonation of GLU hydroxyl or coordinating water ligand) are definitive, likely because of a low number of (co)existing species. By contrast, in the pH range of 5–9 (Sm, Lu) as well as 7–10 (La), speciation is rather complex in terms of coexistence of several species (vide supra). We suppose that deprotonation of the complexes’ coordinating and especially noncoordinating GLU hydroxyl groups is subject to slow kinetics and interrelated dynamic equilibria. These involve exchange between isomers close in energy (vide infra, LaGLUH*x*–2(OH)*x*0) with concomitant alterations in the intra- and intermolecular hydrogen bonding network accompanied by ligand conformation changes. Apparently, these deprotonation reactions, i.e., decrease in pH over time, are significantly slower and thus less progressed for the somewhat weaker Lewis acid La(III) than for the slightly stronger Lewis acid Lu(III) when pH is adjusted to 11. This is likely due to the Ln(III) coordination-induced increase of GLU’s hydroxyl groups’ acidity.

Representative spectra of these alkaline solutions are compiled in Figures S26–S30, Supporting Information. Despite the remarkable pD shift (11.0 → 8.2), the spectrum of the La:GLU 10:30 sample exhibits only very small changes after eight months (Figure S26, Supporting Information). Observed signal displacements are the largest for H-2 but mere 8 ppb. The spectrum of the aged sample becomes better resolved, possibly upon agglomeration of colloids and tiny particles present in the initial sample being removed from the solution by settling. By contrast, the La/GLU ratio appears to have a significant influence on speciation at comparable pD (7.9/8.2). Whereas signals associated with H-4/5/6 reveal (almost) no displacement, those of H-3 and especially H-2 are shifted (and broadened) significantly by 20 and 50 ppb, respectively. Taking into account that the spectra are mole fraction weighted averages, the 10:10 solution contains a larger fraction of dissolved complex species contributing to the spectrum. Again, the spectral features indicate the carboxyl and the C2–O(H) group chelate as the primary binding motif. However, although small but visible, some further signals imply the coexistence of additional species. Unfortunately, the poor intensity hinders assignment.

In case of Lu(III), spectra of the 10:30 samples (pD 11.0 → 9.6) are virtually identical. Of the 10:10 samples (pD 11.0 → 10.5), the signals of the aged solution show some small additional broadening but no signal displacements (Figure S27, Supporting Information). As for the corresponding La(III) sample, weak signals of further species are just discernible. At pD 13, spectra of the freshly prepared samples are similar, showing only small, unspecific differences. The spectrum of the 10:10 solution is somewhat broadened. Upon comparison of the 10:10 and 10:30 solutions with respect to time (freshly prepared vs. after eight months, in various superpositions depicted in Figures S28 and S29, Supporting Information), three main observations are made. Spectra of the 10:30 solutions are almost identical, with that of the aged solution exhibiting an additional set of small signals. For the 10:10 solutions, the spectrum of the aged sample again shows sharper signals and, furthermore, the same additional set of distinct signals as the aged 10:30 sample but of increased intensity.

### Ln(III)–GLU Structure Study by DF Calculations

To complement the experiments on Ln(III)–GLU complexes, quantum chemical calculations for the example of La(III) monogluconate complexes have been carried out for complex charges between +2 and 0*e*. The results can be compared with experimental ones for GLU concentrations not exceeding the La concentration. The computations focus on the determination of coordination modes and numbers of the ligands for various deprotonation states including ternary hydroxo–gluconate complexes.

#### Second Deprotonation of Gluconic Acid

**Figure 8:**
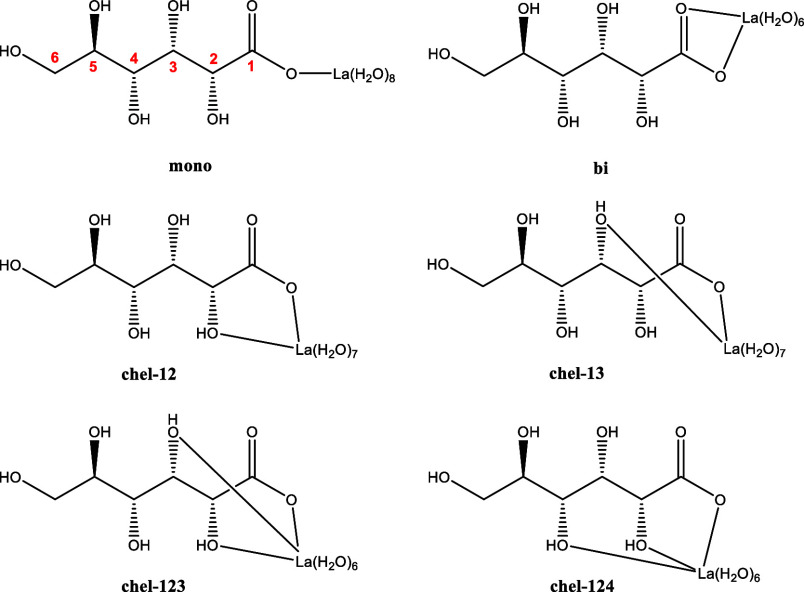
Coordination isomers of LaGLU^2+^: coordination modes of gluconate to La(III) as monodentate (mono), bidentate (bi), or chelate (chel) with two- or threefold coordination. Numbers refer to the positions of functional groups of gluconate involved in the chelate coordination.

The first deprotonation of gluconic acid takes place at the carboxylic group at a pH above 3 (Section Ln(III)–GLU Structure Study by NMR). To determine the site of the second deprotonation, we compared deprotonation Gibbs free energies in aqueous solution at room temperature for the five hydroxyl groups of GLU^–^ at the carbons C2 to C6 (see labeling of C atoms in Figure 8). Deprotonation reaction energies are collected in Table S5, Supporting Information. The lowest energy is obtained for C4, in agreement with our NMR results. Marginally higher energies of 3 and 5 kJ/mol are obtained for C5 and C3, respectively. Hydroxyl groups at C6 and C2 can surely be excluded as candidates for the second deprotonation, as the corresponding energies amount to 25 and 36 kJ/mol, respectively.

#### LaGLU^2+^

The singly deprotonated gluconate ligand, GLU^–^, appears in aqueous solution at a pH of about 3 (p*K*_a_^0^ = 3.92^26^). Thus, monogluconate complexes LaGLU^2+^ are expected at the experimental condition of pH = 4 for GLU concentrations comparable to the La(III) concentration. For this complex, six potential coordination modes have been inspected: monodentate and bidentate via the carboxyl group, bidentate chelate complexes involving the carboxyl group and the hydroxyl groups of C2 (chel-12) or C3 (chel-13), and tridentate chelate coordination with the carboxyl group and either the hydroxyl groups of C2 and C3 (chel-123) or of C2 or C4 (chel-124) (Figure 8).

The La(III) coordination number (CN) of each complex has been varied (Table S7, Supporting Information). For orientation, we determined also the CN = *n* of the La(H_2_O)_*n*_^3+^ aqua complex as well as of the monohydroxo complex La(OH)(H_2_O)_*n*_^2+^ (Table S6, Supporting Information). For La(III), we obtain a preferred CN of 9, in agreement with experimental evidence for early lanthanides,^53^ as well as with the CN for Eu determined by TRLFS in this study (cf. Section TRLFS Study at pH 4). A complex with CN = 8 is calculated by only 9 kJ/mol less stable. For La(OH)(H_2_O)_*n*_^2+^, nearly degenerate energies result for species with CN = 7–9 due to a decreasing number of stabilizing hydrogen bonds in the ligand shell. Taking into account the larger bite angles of some of the coordination modes, CNs of 8–9 are plausible for the La(III) monogluconate complex. For Eu, a CN = 8 was estimated from luminescence decay times in TRLFS (Table 1). For the complexes with monodentate, chel-123, and chel-124 coordination, a CN of 9 was found to be favorable by 6–13 kJ/mol compared to neighboring CNs. For the bidentate coordination, CN = 8 is marginally preferred (4 kJ/mol) and for chel-12 species, CN = 9 and CN = 8 are degenerate. Only for the complex with a chelate structure chel-123, we were able to optimize a species with CN = 10. Attempts for some other structures resulted in geometries showing effectively CN = 9 with an aqua ligand in a second-shell position. Thus, the plausible CN of 9 has been obtained in most cases (Table 4).

**Table 4:** Comparison of Structural Parameters^a^ (in pm) and Relative Energies^b^ (in kJ/mol) of Various Coordination Variants of Gluconate in the Complexes [LaGLU(H_2_O)_*n*_]^2+^

coordination	CN^c^	*n*	La–O_aq_	La–O_car_	La–O_hyd_	La–O_av_	NHB	Δ*G*_rel_
chel-123	9	6	260	250	263	260	3 (2)	0
chel-12	9/8	7	262	248	257	259	4 (3)	7
mono	9	8	262	247		260	6 (3)	9
bi	8/9	6	257	256		257	3 (2)	29
chel-124	9	6	260	245	264	259	1 (1)	37
chel-13	9/8	7	261	248	267	261	5 (3)	41

a Average bond lengths of La to aqua ligands, La–O_aq_; bond lengths of La to carboxyl oxygens of gluconate, La–O_car_; bond lengths of La to coordinated hydroxyl groups of gluconate, La–O_hyd_; average La–O bond lengths, La–O_av_; number of intramolecular hydrogen bonds, NHB, between gluconate and coordinated aquo ligands as well as in parentheses noted the number of hydrogen bonds within the gluconate ligand.

b Relative Gibbs free energies.

c Preferred coordination numbers (CNs) of La. For details, see Table S7, Supporting Information. When species with neighboring CNs are nearly degenerate (Δ*G* < 6 kJ/mol), the CN of the less stable complex is also given, separated by a slash.

Despite a varying coordination mode, most of the complexes listed in Table 4 exhibit rather similar geometry parameters. An exception is the bidentate complex, as it is the only one showing the CN of 8 for La(III) instead of 9. The La–O bond to the carboxyl group of gluconate has a length between 245 and 250 pm. The longer value of 256 pm is calculated for the bidentate complex. Contacts to the protonated hydroxyl groups of gluconate are longer, viz., 257–267 pm. As expected, also the bonds to aqua ligands are longer than to the carboxyl group, measuring 260–262 pm. The lower value of 257 pm calculated for the bidentate complex is because of its lower CN. These results indicate that the bond to the carboxyl is the strongest one in the complexes and that bonds to hydroxyl in gluconate are more comparable with the bonds to aqua ligands. The average La–O distance to all ligands amounts to 259–261 pm for all complexes with CN = 9 and the bidentate complex yields the lower value of 257 pm, again due to its lower coordination number. Calculated results for other CNs confirm the general trend that averaged metal–oxygen bond lengths are proportional to the CN of the metal ion.^54,55^

A comparison of the energies of the various coordination modes for the most stable CN values yields the chel-123 coordination as the most stable geometry (Table 4). Structures with chel-12 and monodentate gluconate coordination are only 7–9 kJ/mol less stable and thus are also plausible to exist. This result is compatible with the findings of NMR spectroscopy showing that besides the carboxyl group, also the OH group at C2 is involved in complexation (cf. Section Ln(III)–GLU Structure Study by NMR). An earlier NMR study of Eu(III) complexation by d-galacturonate suggested also a 3-fold chelate coordination.^56^ The 3-fold coordination via the equatorial carboxyl group, the axial hydroxy group on C4, and the ring oxygen is likely the result of α-d-galacturonate’s prestructuring arising from its cyclic aldopyranose form while gluconate is an open chain sugar acid. The other coordination modes inspected are less stable by at least about 30 kJ/mol (Table 4) and are not expected to contribute to the speciation of La(III) monogluconate. From the most stable structures, we infer that the LaGLU^2+^ complex, which is the dominant species at a pH = 4 for concentrations [La]/[GLU] = 1 (Figure 3), carries 5–8 aqua ligands, with a number of 6 aqua ligands being the most probable one (Table S7, Supporting Information). The estimate of aqua ligands from TRLFS decay times of 8 (Table 1) qualitatively agrees with this result, as an overestimation of the number of aqua ligands might occur due to contributions to the quenching by the hydroxyl groups of gluconate.

#### LaGLUH_*x*–1_(OH)_*x*_^+^–Ln(III) Hydrolysis vs GLU Deprotonation

With increasing pH, La(III) gluconate complexes of lower charge appear due to the increasing degree of deprotonation of gluconate in the field of the La(III) ion. Alternatively, the charge of a La(III) monogluconate complex can be lowered by metal hydrolysis, leading to ternary complexes. We compared relative energies of La(III) monogluconate complexes with a charge of +1*e* in order to determine which of these two processes is favorable. We restricted the comparison to the two most favorable coordination modes determined for [LaGLU(H_2_O)_*n*_]^2+^ and generated [LaGLUH_–1_(H_2_O)_*n*_]^+^ and [LaGLU(OH)(H_2_O)_*n*_]^+^ complexes by deprotonation of various hydroxyl groups of gluconate or of aqua ligands attached to La(III) (see Table 5).

**Table 5:** Comparison of Relative Energies (in kJ/mol) for Various Variants of the Complexes [LaGLUH_–1_(H_2_O)_*n*_]^+^ and [LaGLU(OH)(H_2_O)_*n*_]^+^

complex	coordination^a^	CN^b^	*n*	Δ*G*_rel_
[LaGLUH_–1_(H_2_O)_*n*_]^+^	chel-123*	9/8	6	0
	chel-12*3	8	5	1
	chel-12*	8	6	12
[LaGLU(OH)(H_2_O)_*n*_]^+^	chel-123-OH trans	8	4	14
	chel-123-OH cis	8	4	20
	chel-12-OH trans	8/9	5	26

a For the abbreviations of various coordination modes, see Figure 8 and Table 4; cis and trans denote the position of OH relative to gluconate in the ligand shell of La(III). An asterisk marks the position of the deprotonated hydroxyl group in GLUH_–1_^2+^. The label OH indicates a ternary species with a deprotonated aqua ligand, leading to a hydroxo ligand.

b Preferred CNs of La(III). For details, see Table S8, Supporting Information. When species with neighboring CNs are nearly degenerate (Δ*G* < 6 kJ/mol), the CN of the less stable complex is also given, separated by a slash.

For almost all complexes [LaGLUH_–1_(H_2_O)_*n*_]^+^ and [LaGLU(OH)(H_2_O)_*n*_]^+^ inspected, a preference for a CN of 8 around the La(III) ion is obtained. Only for the chel-123* configuration of [LaGLUH_–1_(H_2_O)_*n*_]^+^ , the species with CN = 9 is by 3 kJ/mol more stable than the one with CN = 8 (Table S8, Supporting Information). This lowering of the CN for the complexes of charge +1*e* compared to the ones with a charge of +2*e* (Table 4) is a result of increased competition between strongly binding anionic ligands (gluconate, hydroxide) and the aqua ligands.

Comparison of the two gluconate coordination modes inspected, chel-123 and chel-12 (Table 5), shows that due to deprotonation of gluconate, their energy difference slightly increases from 7 kJ/mol in [LaGLU(H_2_O)_*n*_]^2+^ (Table 4) to 12 kJ/mol. Thus, also for complexes of charge +1*e*, a 3-fold chelate coordination of gluconate is calculated to be probably preferred. The degeneracy of the coordination modes 123* and 12*3 shows that there is no preference for the deprotonated hydroxyl group involved in the coordination to La(III) and these isomers are likely to exist in a dynamic equilibrium. Relative energies of [LaGLUH_–1_(H_2_O)_*n*_]^+^ and [LaGLU(OH)(H_2_O)_*n*_]^+^ complexes, which differ by a proton shift from an aqua ligand in [LaGLUH_–1_(H_2_O)_*n*_]^+^ to gluconate in [LaGLU(OH)(H_2_O)_*n*_]^+^, show that the binary La–GLU species are slightly more stable than the ternary La–GLU–OH species for both coordination modes of gluconate inspected.

#### LaGLUH_*x*–2_(OH)_*x*_^0^

Charge neutral binary La(III) gluconate and ternary La(III) gluconate hydroxo complexes have been inspected also for the two gluconate coordination modes chel-123 and chel-12 which have been shown to be most favorable for [LaGLU(H_2_O)_*n*_]^2+^ (Table 4). Such complexes have been generated by further deprotonation of the complexes with a charge of +1*e*, either at hydroxyl groups of gluconate or at aqua ligands of La (Table 6).

**Table 6:** Comparison of Relative Energies (in kJ/mol) for Various Variants of the Complexes [LaGLUH_*x*–2_(OH)_*x*_(H_2_O)_*n*_]^0^ for *x* = 0, 1, and 2

complex	coordination^a^	CN^b^	*n*	Δ*G*_rel_
[LaGLUH_–2_(H_2_O)_*n*_]^0^	chel-12*3*	9/8	6	25
	chel-123*-4*^c^	8	5	2
	chel-123*-6*^c^	8	5	100
[LaGLUH_–1_(OH)(H_2_O)_*n*_]^0^	chel-123*-OH cis	8	4	1
	chel-123*-OH trans	8	4	0
	chel-12*3-OH cis	8	4	13
	chel-12*3-OH trans	8	4	20
	chel-12*-OH cis	8	5	13
	chel-12*-OH trans	8	5	38
[LaGLU(OH)_2_(H_2_O)_*n*_]^0^	chel-123-(OH)_2_ cis trans	8	3	13

a For the labeling of coordination modes, see Figure 8 and Table 4; cis and trans denote the position of OH relative to gluconate in the ligand shell of La(III). An asterisk marks the position of deprotonated hydroxyl groups in GLUH_–1_^2+^. The label OH indicates a ternary species with a deprotonated aqua ligand, leading to a hydroxo ligand.

b Preferred CNs of La. For details, see Table S9, Supporting Information. When species with neighboring CNs are nearly degenerate (Δ*G* < 6 kJ/mol), the CN of the less stable species is also given, separated by a slash.

c Besides the coordinated hydroxyl group at C3, also the noncoordinated ones at C4 or C6 are deprotonated, respectively.

In contrast to the complexes of charge +1*e*, a further deprotonation yielding neutral species does not lead to a lowering of the preferred CNs. The charge-neutral complexes show preferentially a CN of 8 (Table 6) as the ones of charge +1*e* (Table 5), with the exception of [LaGLUH_–2_(H_2_O)_*n*_]^0^ with the deprotonation scheme 12*3* (Table 6). Seemingly, deprotonation of gluconate represents a weaker bonding competition to aqua ligand bonding than deprotonation of a coordinating aqua ligand yielding a hydroxo ligand.

For the complex with 3-fold deprotonated gluconate, only two variants are of interest: the one where all functional groups coordinated to La(III) are deprotonated (chel-12*3*) and examples where one of the distant hydroxide groups of gluconate is deprotonated. These latter isomers show a lower energy for deprotonation at C4 and a much higher energy (+75 kJ/mol) for the deprotonation at C6 compared to the first one (Table 6). This demonstrates that also deprotonation of noncoordinating groups leads to favorable stable complexes.

The most stable form of neutral complexes is calculated for ternary ones with a single hydroxo ligand (Table 6) with the coordination mode chel-123*. Cis and trans isomers of this species with respect to the hydroxo ligand are degenerate and only marginally more stable than the binary complex with the deprotonation scheme chel-123*-4*. The alternative deprotonation form chel-12*3 of the ternary complex and the second coordination type chel-12* are less stable by more than 10 kJ/mol (Table 6). Also the complex variant with two hydroxo ligands, chel-123-(OH)_2_, has been found to be somewhat less stable than the most favorable La-GLU-OH ternary complex of charge 0*e*. Overall, the exemplary energy results compared in Table 6 show that for neutral La(III) monogluconate complexes, ternary species with a single hydroxo ligand are essentially energetically degenerate with a binary complex. A previous study employed a different variant of the DF approach (PBE0 functional, smaller basis set, program ORCA) treating three isomers of the neutral Nd(III) monogluconate complex, assuming a fixed coordination number of eight around the metal center.^18^ These calculations support the existence of neutral ternary complexes. However, due to the limited number of structures considered and, most importantly, the fixed coordination numbers, this study missed the thermodynamically most favored isomer and obtained chel-12*-OH cis as the most stable species.^18^ Variants of the gluconate coordination and the protonation scheme of the ternary complexes with one or two hydroxo ligands are relatively close in energy. As some of these species are calculated to be only 13 or 14 kJ/mol less stable than the most stable complex (Table 6), an equilibrium between several neutral complexes can be expected. The results for neutral complexes compared to monocationic ones show that with decreasing positive charge of the complexes, ternary species get competitive to binary ones. Thus, with increasing pH, at the latest for monoanionic species, ternary complexes have to be taken into account in the speciation.

## Discussion

### Binary Ln(III)–GLU Complexes in Solution (pH 4)

**Figure 9:**
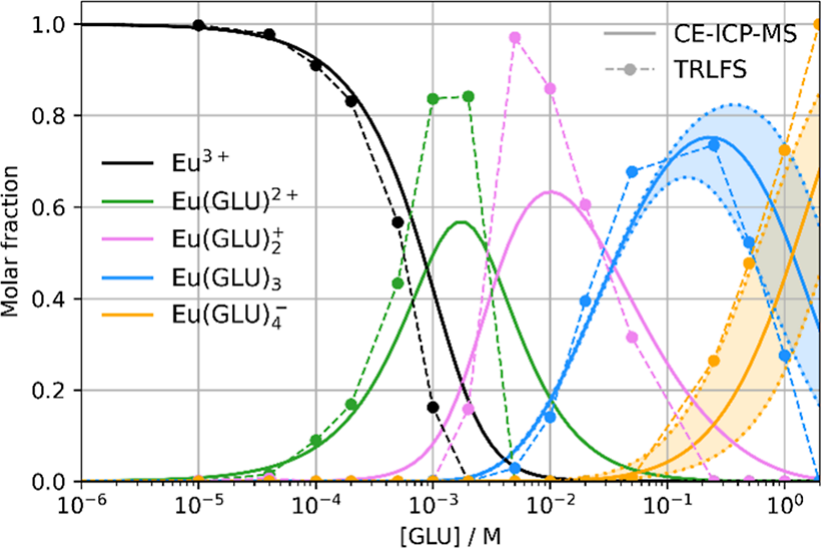
Speciation diagram for 1 mM Eu(III) as a function of [GLU], obtained from TRLFS and PARAFAC (dashed lines). The experimental parameters of the TRLFS experiment were used to calculate the Eu(III) speciation using PHREEQC^57^ (solid lines). For this purpose, the four Eu–GLU complexes determined by CE-ICP-MS (Table 7) were added to the ThermoChimie v12a Davies database.^58^ The pH value was fixed at pH 4 using HCl, and the ionic strength was not fixed and increased according to [NaGLU].

The combination of CE-ICP-MS and TRLFS data was used to determine the number of different Eu–GLU complexes and their respective log β values. To assign a stoichiometry to each of the four complexes observed by TRLFS, the obtained speciation diagram is compared to that calculated from the log β values determined by CE-ICP-MS at pH 4. The calculated complex formation constants were extrapolated to zero ionic strength using the Davies equation (Table 7).^27^ It is interesting to note that both analytical methods were carried out at different Eu(III) concentrations ([Eu(III)] = 1 mM for TRLFS and [Eu(III)] = 1 μM for CE-ICP-MS). Nevertheless, the same complexes were determined in both experiments (see Figure 9), and the Eu(III) speciation obtained by TRLFS and CE-ICP-MS is in very good agreement with each other. That means that the Ln concentration has no influence on the principal speciation and only mononuclear species exist at pH 4.

**Table 7:** Calculated Complex Formation Constants Log β_*i*_^0^ Extrapolated to Zero Ionic Strength

	La(III)	Sm(III)	Eu(III)	Gd(III)	Lu(III)
log β_1_^0^	3.55 ± 0.04	4.01 ± 0.05	3.98 ± 0.06	3.79 ± 0.04	3.92 ± 0.04
log β_2_^0^	6.04 ± 0.04	7.04 ± 0.05	7.04 ± 0.05	6.79 ± 0.04	6.84 ± 0.04
log β_3_^0^	7.70 ± 0.04	8.92 ± 0.06	8.91 ± 0.07	8.70 ± 0.05	8.64 ± 0.05
log β_4_^0^		8.97 ± 0.27	8.92 ± 0.35	8.72 ± 0.24	8.69 ± 0.23

The four Eu–GLU species can be assigned to EuGLU^2+^, EuGLU_2_^+^, EuGLU_3_^0^_(aq)_, and EuGLU_4_^–^, respectively. The TRLFS results support the existence of the Eu(GLU)_4_^–^ complex, which carries a negative charge and forms only at very high GLU excess. Nevertheless, when analyzing the CE-ICP-MS data, it was possible to extrapolate the measured mobilities by adding the 1:4 complex to the fitting model. For a more accurate determination of log β(EuGLU_4_^–^) by CE-ICP-MS, the gluconate concentration and in turn the ionic strength need to be increased, which poses some challenges for CE-ICP-MS and the extrapolation to zero ionic strength. Here, the TRLFS had no limitations and the Eu(GLU)_4_^–^ could be detected.

Complementary to the quantitative evaluation of the Eu–GLU system, also the binding motifs are of interest. For this, the combination of NMR and DF calculations provided qualitative information required for the assignments. NMR experiments and DF calculations mutually support their individual results. The perpetual observation of the strongest Ln(III) complexation-induced ^1^H chemical shift for the signal associated with H2 along with the very small difference in energy calculated for the chel-12 and chel-123 complexes evidences the binding motif to be (primarily) the chelate involving the carboxyl group and the C2–OH group with likely participation of the C3–OH group.

Whether the full binding motif chel-123 is retained also for the binary complexes of higher stoichiometry is debatable. However, at least the principal chel-12 motif appears to constitute the coordination in the 1:2 and 1:3 complexes as seen from the overall increase of log β values (cf. Table 7) upon formation of higher stoichiometric complexes. The decrease in the stepwise complex formation constants (log *K*_*i*_ = log β_*i*+1_ – log β_*i*_) is not surprising as the driving force to add more ligand molecules to the complex reduces due to both a decrease in the net positive charge centered at the metal ion and an increase in steric hindrance lowering the affinity for further ligand binding. The near-zero value of log *K*_4_ indicates the rather loose binding of the fourth ligand owing to the reasons mentioned above, in line with its observation only at very high ligand excess.

The somewhat larger than expected number of about 8, 6, and 4 water molecules remaining in the first coordination sphere of the Eu(III) ion associated with 1:1, 1:2, and 1:3 complexes, inferred from corresponding τ values of 125, 150, and 230 μs, respectively, does not contradict chelate binding motifs (chel-123 and/or chel-12) as they can be explained by the involvement of GLU’s OH group(s). Since luminescence quenching is facilitated by O–H oscillators, those of water being used for the aforementioned calculation of coordinating water ligands, GLU’s coordinating (protonated) O–H groups likely contribute to the quenching resulting in somewhat shorter decay time values than would be expected without the GLU O–H oscillators, causing the calculated *n*(H_2_O) to be overestimated.

### Ln(III)–GLU Complexation at High pH Values

In contrast to samples at pH 4, the formation of polynuclear complexes seems to be favored at pH 10–12. The different speciation below and above pH 7 is reflected in both TRLFS and NMR spectroscopies by fundamental changes of the spectral features. In TRLFS, the spectral signatures changed along with significantly longer corresponding luminescence decay times. NMR spectra exhibited a remarkable drop in complexation-induced signal displacements accompanied by fundamental changes in the signals’ appearance (Figures S22–S25, Supporting Information). As these changes in complexation behavior fall in a range of both commencing Ln(III) hydrolysis as well as deprotonation of GLU’s coordinating OH groups upon Lewis acid-induced decrease in associated p*K*_a_ values, DF calculations were employed to examine these processes individually. Both processes cause a decrease in the metal center’s Lewis acidity translating into diminishing downfield shifts in ^1^H NMR spectra. Notably, regardless of which of the coordinating GLU OH groups undergoes deprotonation (chel-123* vs chel-12*3), the degeneracy in energies is in line with the multitude of NMR signals observed for Lu(III) at pH 6 (Figure S23) as well as the (extremely) broadened signals in the spectra of the La(III) for pH 7 and 8 and Sm(III) for pH 6 and 7, respectively. Coexisting species close in energy can easily interconvert and, depending on the time regime of coordinating GLU O(H) de/protonation processes, NMR signals broaden and/or average. Correspondingly, the Ln(III)-induced decrease in GLU–OH p*K*_a_ values can be estimated to be around 8, 7, and 6 in case of La, Sm, and Lu, respectively, mirroring the increase in the metals’ increasing Lewis acidity along the Ln series.

Upon further reducing the charge in the GLU complexes, equivalent to further H^+^ removal, three remarkable results are inferred from DF calculation. That is, first, deprotonation of noncoordinating GLU OH groups is also a valid process to stabilize the yielded complex. Interestingly, this is most likely at C4–OH (in chel-123*-4*) which is the one OH group being most acidic in the free ligand as calculated by DF theory and determined by NMR. Deprotonation of noncoordinating GLU OH groups matches spectra at high pH where the magnitude of the downfield shifts is remarkably smaller but now more uniform among all GLU ^1^H NMR signals. Second, deprotonation of noncoordinating C4–OH is practically isoenergetic to hydrolysis of a coordinating water ligand (chel-123*-OH). Third, species comprising the C2–OH deprotonation and water ligand hydrolysis or hydrolysis of two coordinating water ligands instead of any GLU OH deprotonation are all comparable in energy and only somewhat less stable as the lowest energy configurations above. Species varying in their deprotonation pattern but similar in energy are in full agreement with the Lu(III) solutions investigated in a (hyper)alkaline medium, showing pH drift upon slow deprotonation kinetics at moderate basicity but being fast (and pH stable) in a strongly alkaline medium.

At high pH values, aggregates, which precipitate over time, were observed by TRLFS. With CE-ICP-MS at [Ln(III)] = 1 μM, solvated species were only observed in fresh samples, and aged samples (after 6 weeks equilibration) also showed signs of aggregates. For the fresh samples, CE-ICP-MS results show predominantly negatively charged complexes, suggesting formation of Ln(III)GLU_*i*_H_–*j*_. Formation of colloids could occur via hydroxo-gluconate-complexes and/or hydroxo bridges or cross-linking by further deprotonated GLU ligands. Moreover, CE-ICP-MS results indicate a change in speciation for the solvated species forming at pH 10–12 indicating a strong influence of the pH value in this pH range. In the beginning, first aggregates might be formed which then subsequently undergo a ripening process leading to precipitation. The Eu(III) ions captured in the precipitate are not all located in exactly the same chemical environment but with slightly different locations with respect to distance to the neighboring atoms. As a consequence, in the PARAFAC analysis of the TRLFS data of the precipitate, similar emission spectra are found but with larger variations in the luminescence decay kinetics. This observation is the result of the distribution as described above. Variations in the decay kinetics are due to differences in the self-quenching efficiencies of Eu(III) ions. Therefore, at different GLU concentrations, the observed luminescence decay times are different.

The formation of aggregates was cross-checked with TRLFS by investigating samples with combinations of two Ln(III) ions, for which the occurrence of RET is indicative of aggregation. In addition, ultracentrifugation was applied. Here, for freshly ultracentrifuged samples, a distinct decrease of the TRLFS signal was found, which reoccurred after some weeks. The reformation of aggregates from the remaining Eu–GLU species in solution appeared to be a slow process because the TRLFS data were changing over time even after several weeks after ultracentrifugation. This seems to be in agreement with the findings of the CE-ICP-MS experiments carried out at pH 10 and at low [Eu(III)], where the formation of aggregates also took place over several weeks.

## Conclusions

We investigated the complexation of the common cement additive gluconate with various trivalent lanthanide ions. TRLFS, CE-ICP-MS, NMR, and DF calculations have been applied to achieve a detailed picture of the complexation in a wide concentration and pH range, considerably complementing and expanding the results of previous studies.

The methodological approach in this study allowed for extended insights into the complexation behavior of trivalent lanthanides toward gluconate, the resulting speciation, and corresponding structure–properties relationships, covering several orders of magnitude in concentration and Ln(III):GLU ratios for aqueous solutions in the pH range 4 through 13.

Among the investigated Ln(III) ions, the complexation behavior is similar in general but shows minor differences along the Ln series arising from the decrease of the ionic radius and the resulting increasing Lewis acidity, which influences the thermodynamic and kinetic behavior of the aqueous complexes.

In slightly acidic up to circumneutral solutions, regardless of the Ln(III) concentration regime (micromolar through millimolar), binary complexes of Ln(III)/GLU with stoichiometric ratios of 1:1 through 1:4 form. Increasing the number of coordinating ligands requires progressively increasing ligand excess to compensate for both charge and steric effects. Coordination via the carboxyl group and the protonated OH group of the adjacent carbon (C2) is facilitated by formation of a five-membered ring chelation motif, with a probable participation of the C3–OH. At circumneutral pH, with the exact onset depending on the nature of the Ln(III) ion, a fundamental change in speciation takes place as mirrored in corresponding spectral signatures (luminescence decay time in TRLFS, smaller shifts and increased broadening in NMR) as well as measured (electrophoretic mobility, CE) mean charges, and calculated relative Gibbs energies (from DF calculations) of predominating (low energy) complexes. Speciation for higher pH values becomes more complex upon coexistence and interconversion of several complexes and isomers which involve one or more deprotonated GLU hydroxyl groups not necessarily participating in coordination. For charge-neutral complexes, comparable energies are calculated when protons are exchanged between aqua and the gluconate ligand, showing that binary gluconate and ternary hydroxo gluconate complexes are in equilibrium. However, as these calculations refer to complexes of a Ln(III):GLU ratio of 1:1, in solutions with sufficient ligand excess, ternary complexes are likely outcompeted.

Alkaline solutions ≥1 mM in Ln(III) featured turbidity and formation of multinuclear aggregates. Even for 1 μM Ln(III), eventually (incomplete) precipitation with slow formation kinetics over several weeks was observed. In fresh alkaline solutions, we were able to demonstrate the ability of GLU to bind Eu(III) at [GLU] > 10^–5^ M. At pH 10, the speciation was found to be dominated by the proposed [Eu(GLU)_2_H_–2_]^−^ complex. This species is still able to interact with inorganic cementitious constituents such as calcium and magnesium, forming heterobimetallic (quaternary) Ca/Mg-Ln(III)/An(III)-(OH)-gluconate complexes, further adding to the complexity of cement pore water systems.^14,16,48,59^ Taking into account the expected maximum concentration of gluconate in a potential LLW/ILW repository of 5.1 × 10^–6^ M^60^ as well as additional (competing) retention phenomena such as adsorption, incorporation, or (co)precipitation, the influence of gluconate on the total actinide speciation is insignificant.^8,10,13,61^

## Supporting Information Available

Additional TRLFS, CE-ICP-MS, and NMR analytical data as well as coordination data from DF calculations (PDF)
