# Supplementary material for: Complexation of Ln(III) Ions by Gluconate: Joint Investigation Applying TRLFS, CE-ICP-MS, NMR, and DF Calculations
Source: Inorg Chem. 2025 Apr 11;64(16):7970–87. doi: 10.1021/acs.inorgchem.4c05476 (PMC12042262; doi:10.1021/acs.inorgchem.4c05476)
Supplement: Supplementary file 1 — ic4c05476_si_001.pdf [file ic4c05476_si_001.pdf]

SUPPORTING INFORMATION

Complexation of Ln(III) ions by gluconate: Joint investigation applying TRLFS, CE-ICP-MS, NMR, and DF calculations

Sophie Zenker,[1,†] Janik Lohmann,[2,†] Ion Chiorescu,[3] Sven Krüger,[3]
Michael U. Kumke,[1] Tobias Reich,[2] Katja Schmeide,[4] Jerome Kretzschmar[4,*]

[1] *Universität Potsdam, Institute of Chemistry, 14476 Potsdam, Germany*
[2] *Johannes Gutenberg-Universität Mainz, Department of Chemistry, 55128 Mainz, Germany*
[3] *Technische Universität München, Chemistry Department, School of Natural Sciences, 85748 Garching, Germany*
[4] *Helmholtz-Zentrum Dresden–Rossendorf, Institute of Resource Ecology, 01328 Dresden, Germany*

† These authors contributed equally to this work.
* Corresponding author: j.kretzschmar@hzdr.de

The Supporting Information comprises 32 pages,
including 30 figures, 9 tables, and 1 reference.

Contents

1. **Time-resolved laser-induced luminescence spectroscopy (TRLFS)**
2. **Capillary electrophoresis-inductively coupled plasma mass spectrometry (CE-ICP-MS)**
3. **Nuclear magnetic resonance spectroscopy (NMR)**
4. **Density functional (DF) calculations**

1. *Time-resolved laser-induced luminescence spectroscopy (TRLFS)*

TRLFS study at pH 4

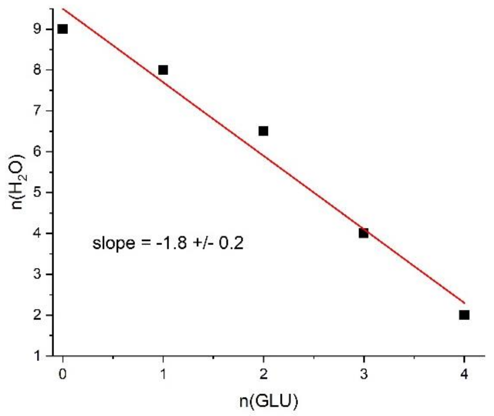

Figure S1. Change in the apparent number of water molecules in the first coordination sphere upon increasing the number of coordinating GLU ligand molecules. The number of water molecules was calculated using Eq. (5) and the decay times shown in Table 1.

Energy transfer experiments

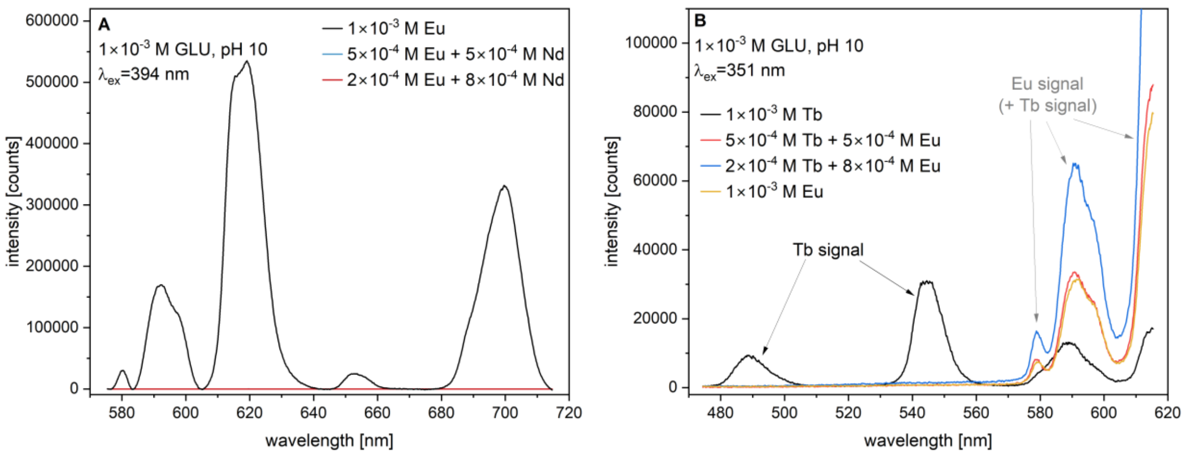

Figure S2. Eu(III) luminescence spectra of the Eu(III)→Nd(III) energy transfer samples with [Eu(III)+Nd(III)]=1×10^{-3} M, [GLU]=1×10^{-3} M and pH 10 upon excitation of the Eu(III) ions at 394 nm (A) and Tb(III) and Eu(III) luminescence spectra of the Tb(III)→Eu(III) energy transfer samples with [Tb(III)+Eu(III)]=1×10^{-3} M, [GLU]=1×10^{-3} M and pH 10 upon excitation at 351 nm, where mainly Tb(III) ions are excited (B). Both figures clearly indicate that an energy transfer takes place as the Eu(III) luminescence is completely quenched by the presence of Nd(III), the Tb(III) luminescence is completely quenched by the presence of Eu(III) and the Eu(III) signal intensity upon excitation at 351 nm increases in the presence of Tb(III). Since the Ln(III) ions must be in close proximity to each other for an energy transfer to take place, and since no solid was visible with the eye, the dominant species under these conditions, which corresponds to the species with a luminescence decay time of (170±30) µs in the pure Eu(III) sample (cf. PARAFAC results in Figure 4), is most likely colloidal.

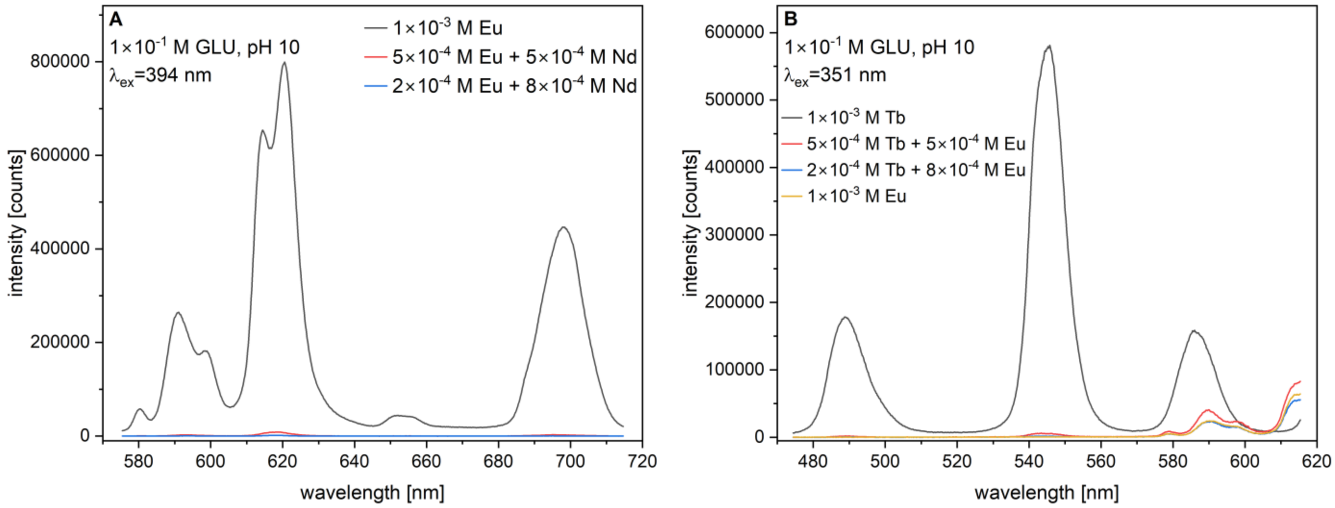

Figure S3. Eu(III) luminescence spectra of the Eu(III)→Nd(III) energy transfer samples with [Eu(III)+Nd(III)]=1×10^{-3} M, [GLU]=1×10^{-1} M and pH 10 upon excitation of the Eu(III) ions at 394 nm (A) and Tb(III) and Eu(III) luminescence spectra of the Tb(III)→Eu(III) energy transfer samples with [Tb(III)+Eu(III)]=1×10^{-3} M, [GLU]=1×10^{-1} M and pH 10 upon excitation at 351 nm (B). Both figures indicate that an energy transfer takes place, suggesting that the species with a luminescence decay time of (590±10) µs in the pure Eu(III) sample (cf. PARAFAC results in Figure 4), which is the dominant species under these conditions, is colloidal as well.

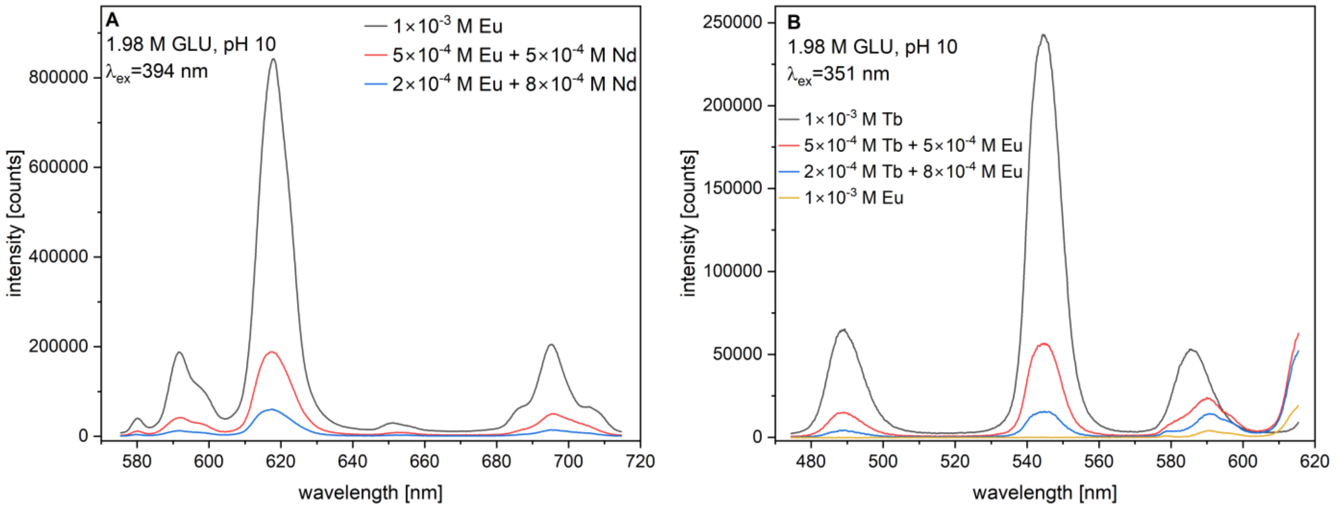

Figure S4. Eu(III) luminescence spectra of the Eu(III)→Nd(III) energy transfer samples with [Eu(III)+Nd(III)]=1×10^{-3} M, [GLU]=1.98 M and pH 10 upon excitation of the Eu(III) ions at 394 nm (A) and Tb(III) and Eu(III) luminescence spectra of the Tb(III)→Eu(III) energy transfer samples with [Tb(III)+Eu(III)]=1×10^{-3} M, [GLU]=1.98 M and pH 10 upon excitation at 351 nm (B). Both figures indicate that a (weak) energy transfer takes place as the Eu(III) luminescence intensity and decay time are reduced by the presence of Nd(III) (the decay time decreased from (534±2) µs in the pure Eu(III) sample to (452±3) µs in the sample with 8×10^{-4} M Nd(III)) and the Tb(III) luminescence intensity and decay time are reduced by the presence of Eu(III) (the decay time decreased from (1400±6) µs in the pure Tb(III) sample to (1200±20) µs in the sample with 8×10^{-4} M Eu(III)). This suggests that the species with a luminescence decay time of (500±20) µs in the pure Eu(III) sample (cf. PARAFAC results in Figure 4), which is the dominant species under these conditions, is colloidal as well. However, the Ln(III)-Ln(III) distance in this species might be larger than in the other species at pH 10, because the energy transfer efficiency is lower than in the other species (as the luminescence intensity and decay time are only slightly reduced, while in the other species the luminescence was quenched completely and no decay time was measurable).

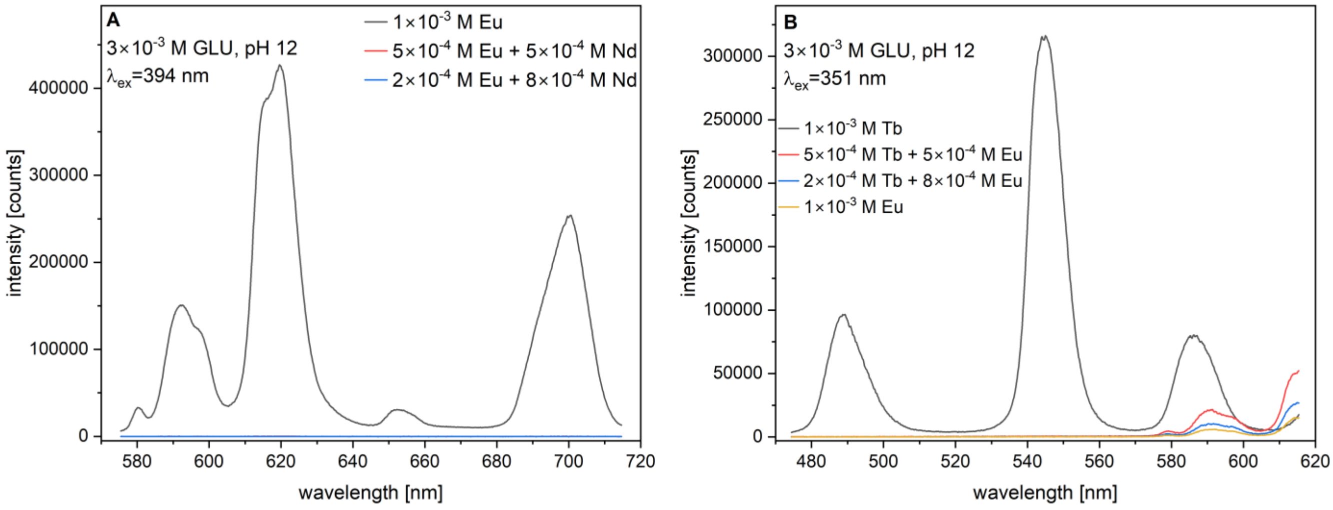

Figure S5. Eu(III) luminescence spectra of the Eu(III)→Nd(III) energy transfer samples with [Eu(III)+Nd(III)]=1×10^{-3} M, [GLU]=3×10^{-3} M and pH 12 upon excitation of the Eu(III) ions at 394 nm (A) and Tb(III) and Eu(III) luminescence spectra of the Tb(III)→Eu(III) energy transfer samples with [Tb(III)+Eu(III)]=1×10^{-3} M, [GLU]=3×10^{-3} M and pH 12 upon excitation at 351 nm (B). Both figures clearly indicate that an energy transfer takes place, suggesting that the species with a luminescence decay time of (440±10) µs in the pure Eu(III) sample (cf. PARAFAC results in Figure 5), which is the dominant species under these conditions, is most likely colloidal.

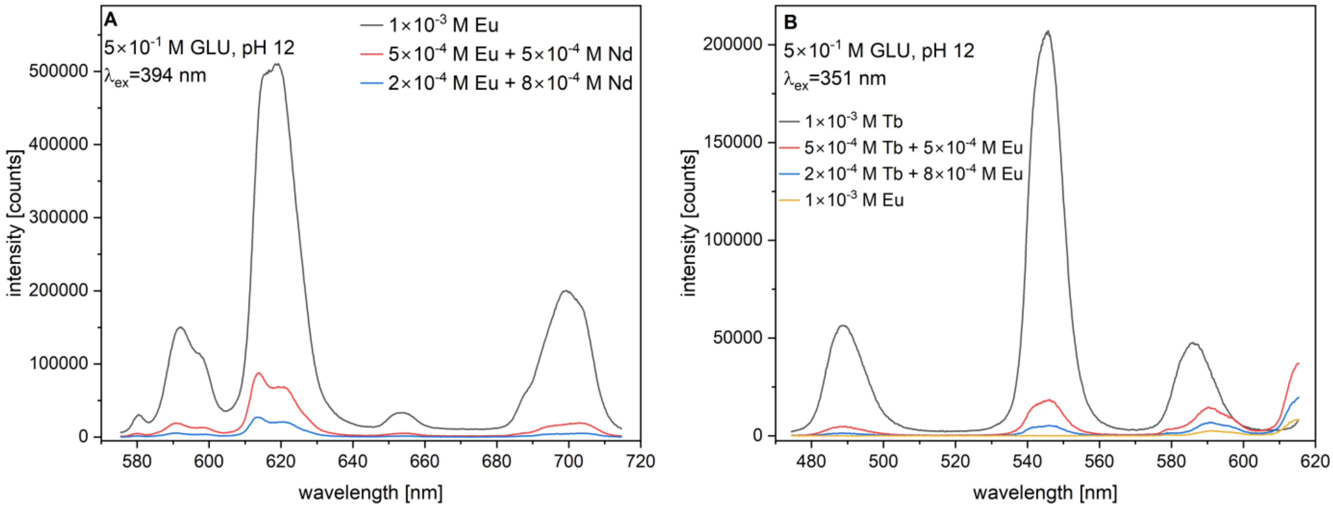

Figure S6. Eu(III) luminescence spectra of the Eu(III)→Nd(III) energy transfer samples with [Eu(III)+Nd(III)]=1×10^{-3} M, [GLU]=5×10^{-1} M and pH 12 upon excitation of the Eu(III) ions at 394 nm (A) and Tb(III) and Eu(III) luminescence spectra of the Tb(III)→Eu(III) energy transfer samples with [Tb(III)+Eu(III)]=1×10^{-3} M, [GLU]=5×10^{-1} M and pH 12 upon excitation at 351 nm (B). Both figures clearly indicate that an energy transfer takes place, suggesting that the species with a luminescence decay time of (600±10) µs in the pure Eu(III) sample (cf. PARAFAC results in Figure 5), which is the dominant species under these conditions, is most likely colloidal. However, some Eu(III) and Tb(III) luminescence with a different spectral shape remains, indicating that a second species is present, which is not quenched by an Eu(III)→Nd(III) or Tb(III)→Eu(III) energy transfer. This is the same species that also appeared at [GLU] > 2×10^{-1} M at pH 12 and pH 13 (vide infra).

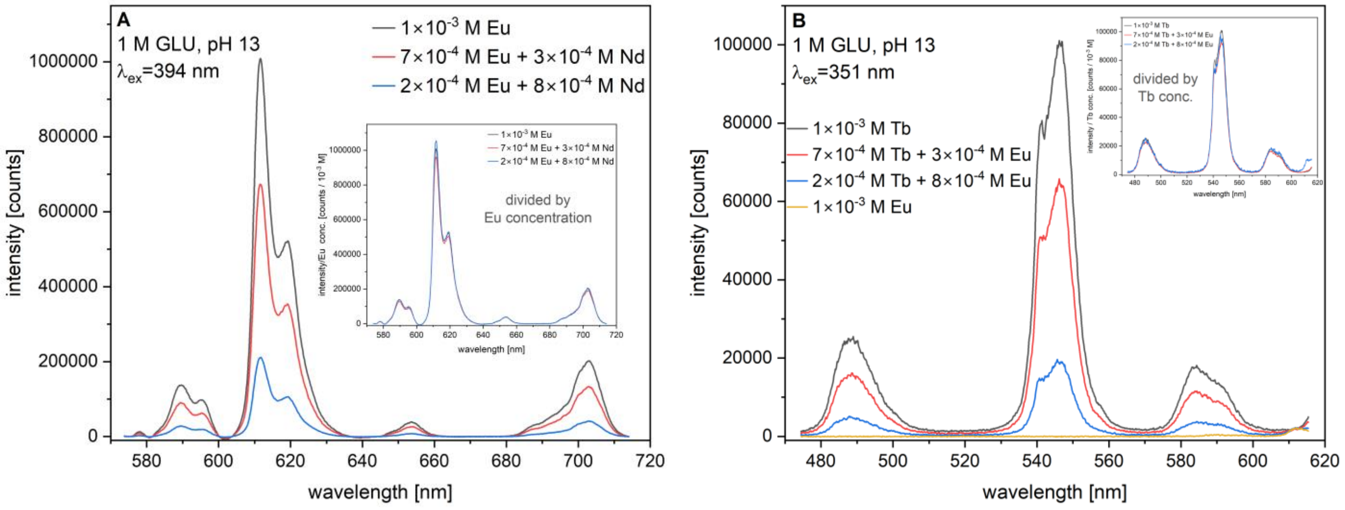

Figure S7. Eu(III) luminescence spectra of the Eu(III)→Nd(III) energy transfer samples with [Eu(III)+Nd(III)]=1×10^{-3} M, [GLU]=1 M and pH 13 upon excitation of the Eu(III) ions at 394 nm (A) and Tb(III) and Eu(III) luminescence spectra of the Tb(III)→Eu(III) energy transfer samples with [Tb(III)+Eu(III)]=1×10^{-3} M, [GLU]=1 M and pH 13 upon excitation at 351 nm (B). In both cases, no energy transfer was observable. The absolute Eu(III) and Tb(III) luminescence intensities (corrected for the different Eu(III) or Tb(III) concentrations in the samples) were unchanged by the presence of Nd(III) for the Eu(III) luminescence and of Eu(III) for the Tb(III) luminescence as shown in the insets. Additionally, the Eu(III) decay time was (540±20) µs in the presence and absence of Nd(III) and the Tb(III) decay time was (1780±20) µs with and without Eu(III). While this experiment was carried out at pH 13, the luminescence spectrum and decay time of the observed species match the spectral features of the species found at pH 12 at [GLU]=1 M (at pH 12 a luminescence decay time of (510±10) µs was found for this species with PARAFAC as shown in Figure 5). The results suggest that this species is either a solvated complex or a colloid with Ln(III)–Ln(III) distances that are too large for an energy transfer to occur.

Ultracentrifuged samples

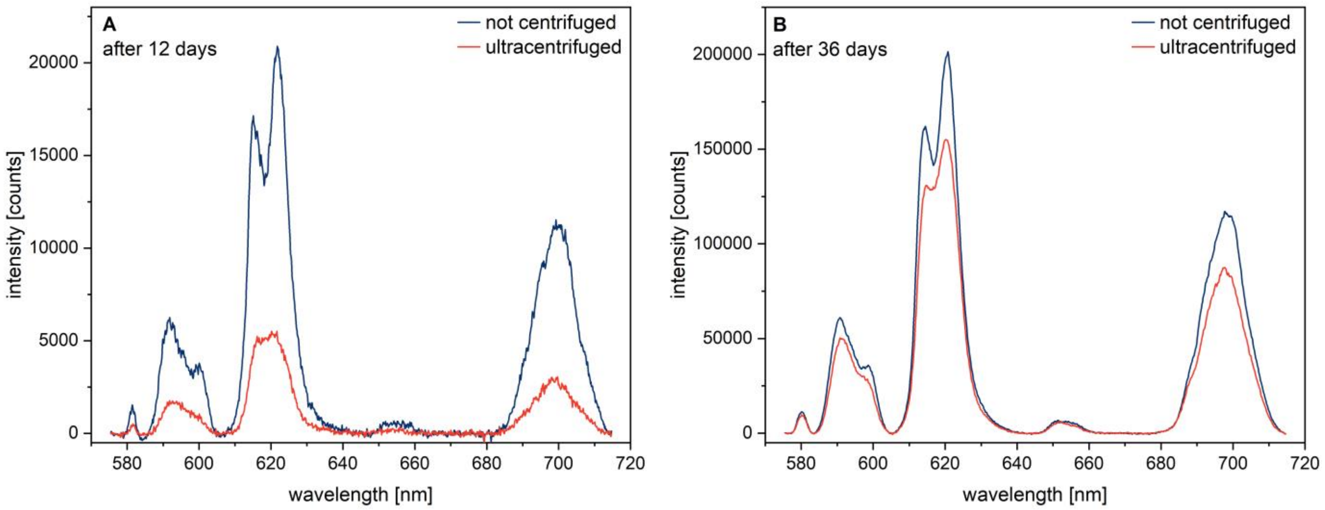

Figure S8. Luminescence spectra of the ultracentrifuged sample 12 days after ultracentrifugation (A) and 36 days after ultracentrifugation (B) in comparison to the reference sample (1×10^{-3} M Eu(III), 2×10^{-2} M GLU, pH 10) that was not centrifuged.

2. *Capillary electrophoresis-inductively coupled plasma mass spectrometry (CE-ICP-MS)*

Table S1. Assumed electrophoretic mobilities μ_i of each Ln(III) species as well as quotients Q for the analyzed lanthanides. The mobility $\mu(Ln^{3+})$ was measured in absence of gluconate. To estimate the other mobilities, the quotient Q of the charge Z_i and the measured mobility μ_i was calculated. Under the assumption that Q does not change for a given system, mobilities were estimated based on the charge of the complex. For neutral complexes, μ_i was set to zero.

	**La(III)**	**Sm(III)**	**Eu(III)**	**Gd(III)**	**Lu(III)**
Q / 10^4 Vs/cm^2	0.63	0.66	0.66	0.66	0.68
$\mu(Ln^{3+})$ / 10^{-4} cm^2/Vs	4.76	4.58	4.54	4.58	4.44
$\mu(LnGLU^{2+})$ / 10^{-4} cm^2/Vs	3.17	3.05	3.03	3.05	2.96
$\mu(LnGLU_2^{+})$ / 10^{-4} cm^2/Vs	1.59	1.53	1.51	1.53	1.48
$\mu(LnGLU_{3(aq)})$ / 10^{-4} cm^2/Vs	0.00	0.00	0.00	0.00	0.00
$\mu(LnGLU_4^{-})$ / 10^{-4} cm^2/Vs	—	−1.53	−1.51	−1.53	−1.48
$\mu(Ln(OH)_{3(aq)})$ / 10^{-4} cm^2/Vs	0.00	—	0.00	—	0.00
$\mu(LnGLU_2H_{-2}^{-})$ / 10^{-4} cm^2/Vs	−1.10	—	−1.12	-	−1.17

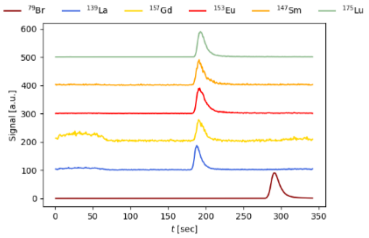

[GLU] = 0 M

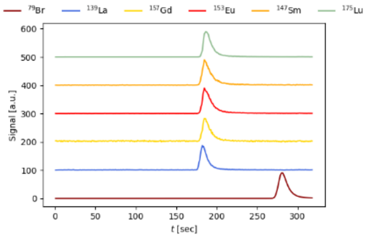

[GLU] = 1×10^{-6} M

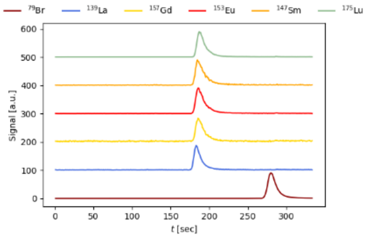

[GLU] = 1×10^{-5} M

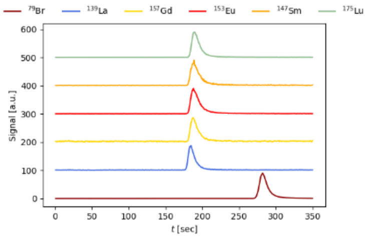

[GLU] = 1×10^{-4} M

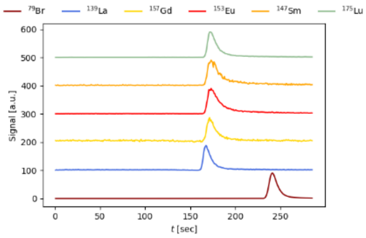

[GLU] = 2×10^{-4} M

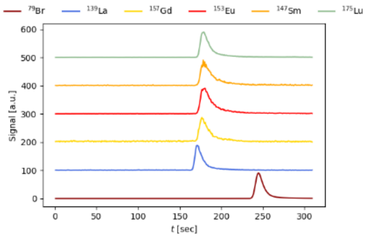

[GLU] = 5×10^{-4} M

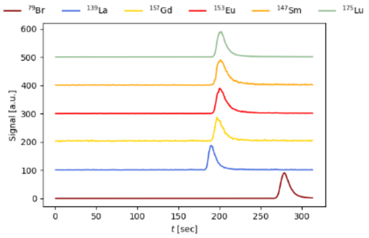

[GLU] = 1×10^{-3} M

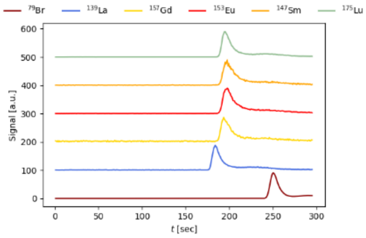

[GLU] = 2×10^{-3} M

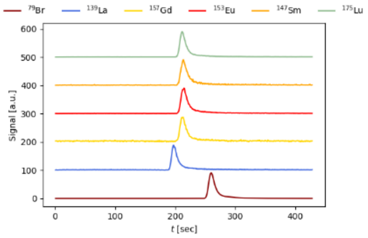

[GLU] = 5×10^{-3} M

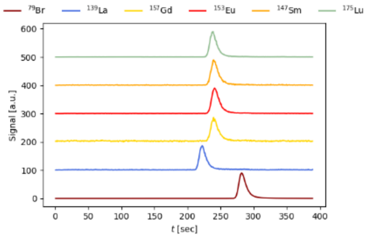

[GLU] = 1×10^{-2} M

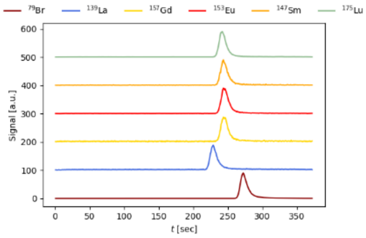

[GLU] = 2×10^{-2} M

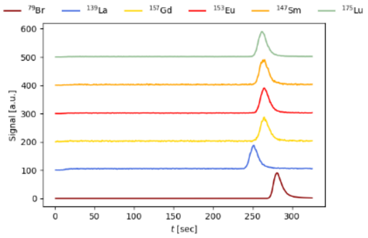

[GLU] = 5×10^{-2} M

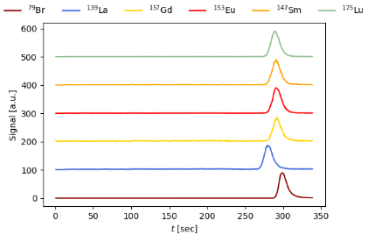

[GLU] = 1×10^{-1} M

Figure S9. Electropherograms of ^{139}La, ^{157}Gd, ^{153}Eu, ^{147}Sm, ^{157}Lu, and ^{79}Br (EOF) at pH 4 and various [GLU], I = 0.1 M ($NaClO_4$), 25 °C, normalized signal, l = 50 cm, measured at 10 kV.

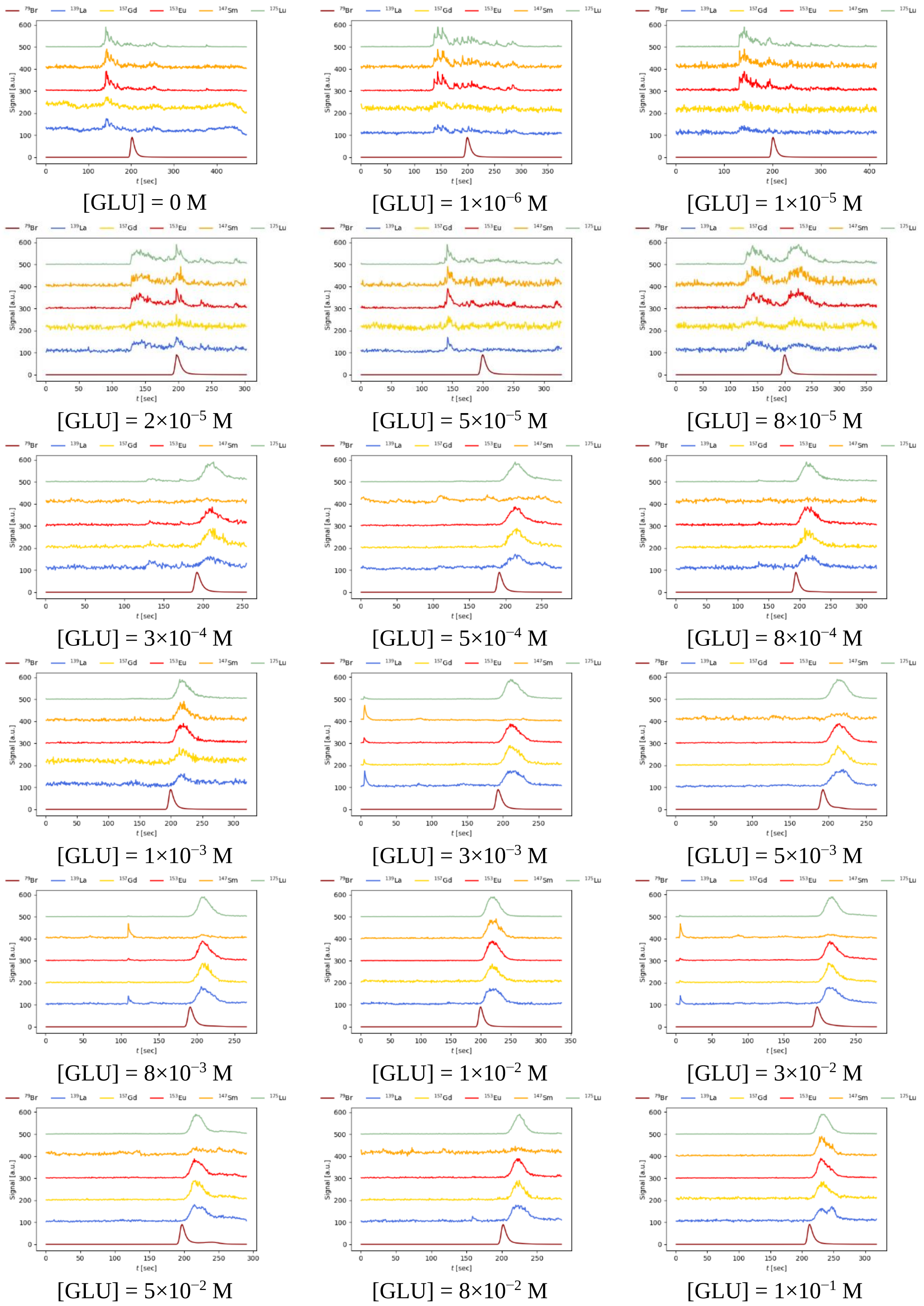

Figure S10. Electropherograms of ^{139}La, ^{157}Gd, ^{153}Eu, ^{147}Sm, ^{157}Lu, and ^{79}Br (EOF) at pH 10 and various [GLU], I = 0.1 M ($NaClO_4$), 25 °C, normalized signal, l = 50 cm, measured at 10 kV, 10 min after preparation.

Table S2. Effective electrophoretic mobilities μ_{eff} [10^{-4} cm^2/Vs] of La, Sm, Eu, Gd, and Lu at pH ~4.

[GLU] / M	pH	$[GLU]_{free}$ / M	La(III)	Sm(III)	Eu(III)	Gd(III)	Lu(III)
1.00×10^{-6}	3.94	6.35×10^{-7}	4.83	4.64	4.64	4.64	4.53
1.00×10^{-5}	3.68	4.88×10^{-6}	4.74	4.62	4.56	4.56	4.44
1.00×10^{-4}	3.82	5.69×10^{-5}	4.67	4.38	4.43	4.49	4.32
2.00×10^{-4}	4.17	1.49×10^{-4}	4.55	4.06	4.06	4.20	4.13
5.00×10^{-4}	4.11	3.60×10^{-4}	4.41	3.80	3.67	3.93	3.74
1.00×10^{-3}	3.87	5.97×10^{-4}	4.21	3.47	3.51	3.72	3.41
2.00×10^{-3}	4.23	1.54×10^{-3}	3.60	2.64	2.64	2.91	2.80
5.00×10^{-3}	4.16	3.71×10^{-3}	3.07	2.06	2.01	2.10	2.19
1.00×10^{-2}	4.20	7.52×10^{-3}	2.38	1.57	1.50	1.57	1.61
2.00×10^{-3}	4.20	1.52×10^{-2}	1.74	1.08	1.08	1.08	1.15
5.00×10^{-3}	4.22	3.84×10^{-2}	1.04	0.52	0.55	0.55	0.67
1.00×10^{-1}	4.32	8.07×10^{-2}	0.59	0.23	0.23	0.21	0.26

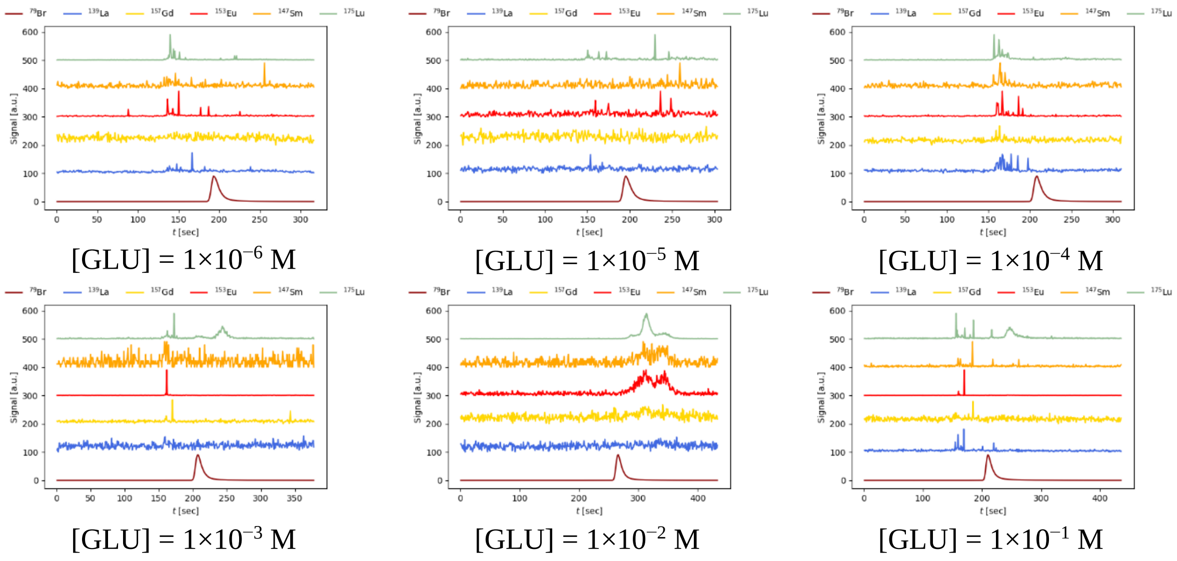

Figure S11. Electropherograms of ^{139}La, ^{157}Gd, ^{153}Eu, ^{147}Sm, ^{157}Lu, and ^{79}Br (EOF) at pH 10 and various [GLU], I = 0.1 M ($NaClO_4$), 25 °C, normalized signal, l = 50 cm, measured at 10 kV after 6 weeks.

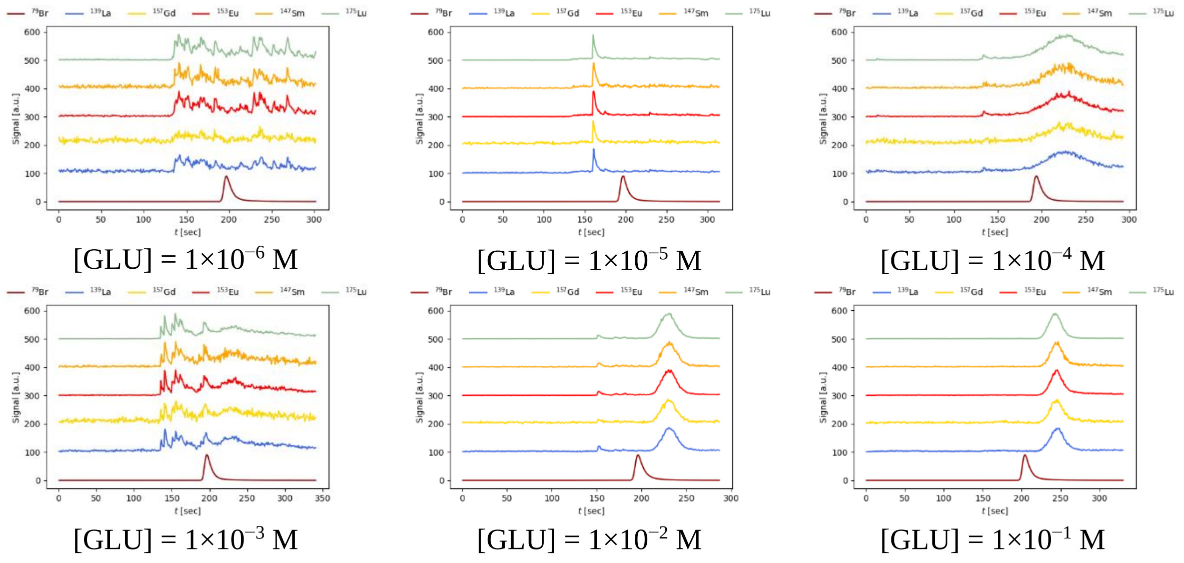

Figure S12. Electropherograms of ^{139}La, ^{157}Gd, ^{153}Eu, ^{147}Sm, ^{157}Lu, and ^{79}Br (EOF) at pH 12 and various [GLU], I = 0.1 M ($NaClO_4$), 25 °C, normalized signal, l = 50 cm, measured at 10 kV.

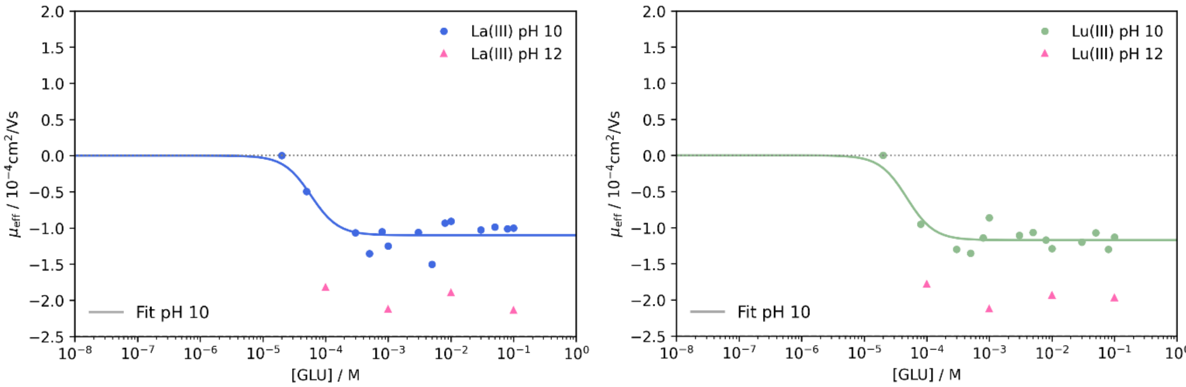

Figure S13. Plots of the measured electrophoretic mobilities μ_{eff} of La(III) and Lu(III) (1×10^{-6} M) against [GLU] at pH 10 and pH 12 and I = 0.1 M ($NaClO_4$). The fit corresponds to the $LnGLU_2H_{-2}^{-}$ complex using equation 10, R^2_{La} = 0.780, R^2_{Lu} = 0.829.

Table S3. Effective electrophoretic mobilities μ_{eff} [10^{-4} cm^2/Vs] of La, Eu, and Lu at pH 10.

**[GLU]$_{free}$ / M**	**La(III)**	**Eu(III)**	**Lu(III)**
2.0×10^{-5}	0.00	0.00	0.00
5.0×10^{-5}	−0.49	—	—
8.0×10^{-5}	—	−0.90	−0.95
3.0×10^{-4}	−1.07	−1.21	−1.30
5.0×10^{-4}	−1.35	−1.24	−1.35
8.0×10^{-4}	−1.05	−1.14	−1.14
1.0×10^{-3}	−1.25	−1.16	−0.86
3.0×10^{-3}	−1.06	−1.06	−1.11
5.0×10^{-3}	−1.50	−1.25	−1.07
8.0×10^{-3}	−0.93	−1.03	−1.17
1.0×10^{-2}	−0.91	−1.12	−1.29
3.0×10^{-2}	−1.03	−1.07	−1.20
5.0×10^{-2}	−0.99	−0.99	−1.07
8.0×10^{-2}	−1.01	−1.18	−1.30
1.0×10^{-1}	−1.00	−0.93	−1.13

Table S4. Effective electrophoretic mobilities μ_{eff} [10^{-4} cm^2/Vs] of La, Eu, and Lu at pH 12.

**[GLU]$_{free}$ / M**	**La(III)**	**Eu(III)**	**Lu(III)**
1.0×10^{-4}	−1.82	−1.98	−1.78
1.0×10^{-3}	−2.12	−1.98	−2.12
1.0×10^{-2}	−1.90	−1.90	−1.93
1.0×10^{-1}	−2.14	−2.07	−1.97

3. *Nuclear magnetic resonance spectroscopy (NMR)*

pH-titration series of GLU only

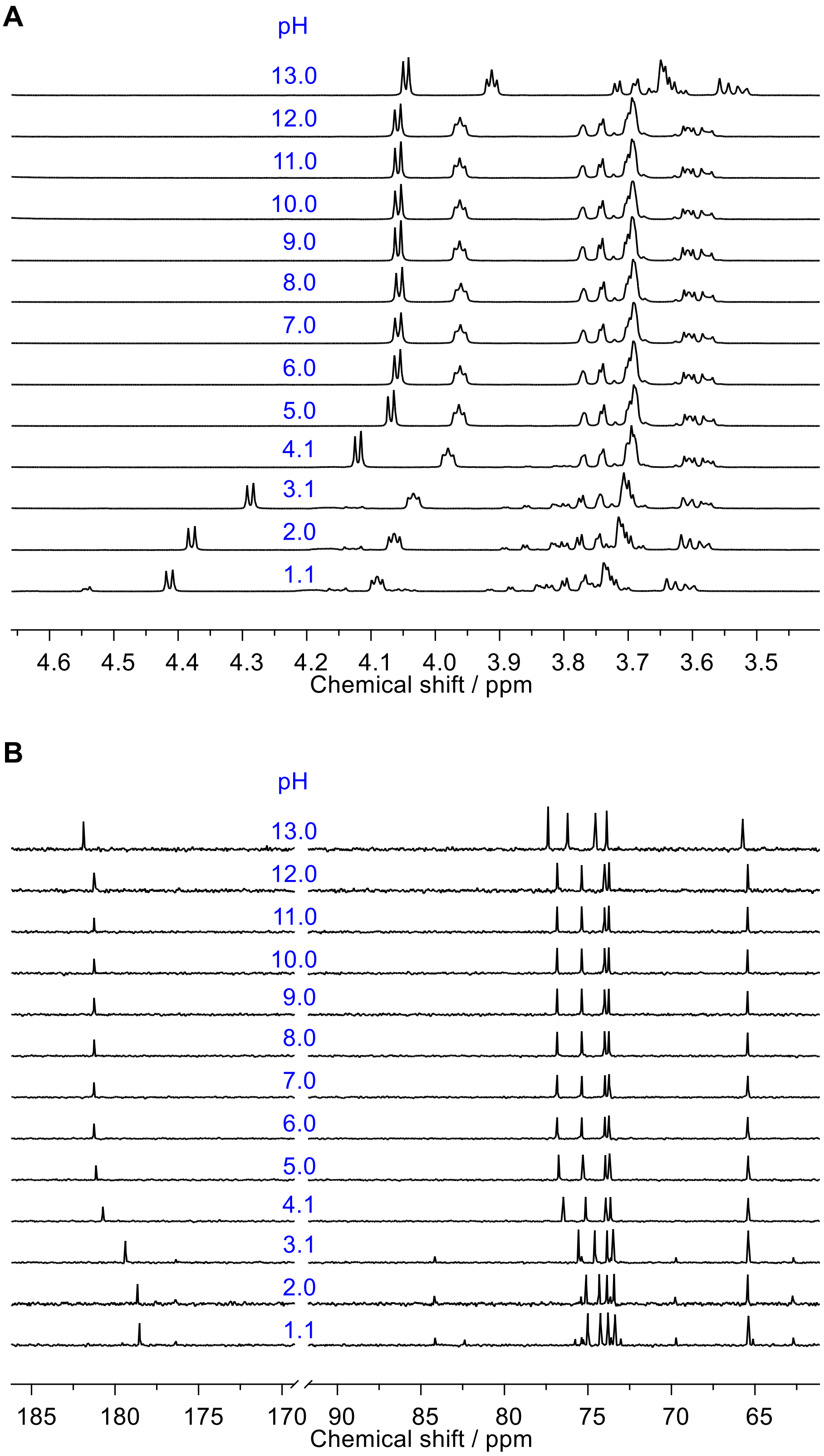

Figure S14. ^{1}H (A) and $^{13}C\{^{1}H\}$ NMR (B) pH-titration spectra of gluconic acid in aqueous solution containing 10% (v/v) D_2O.

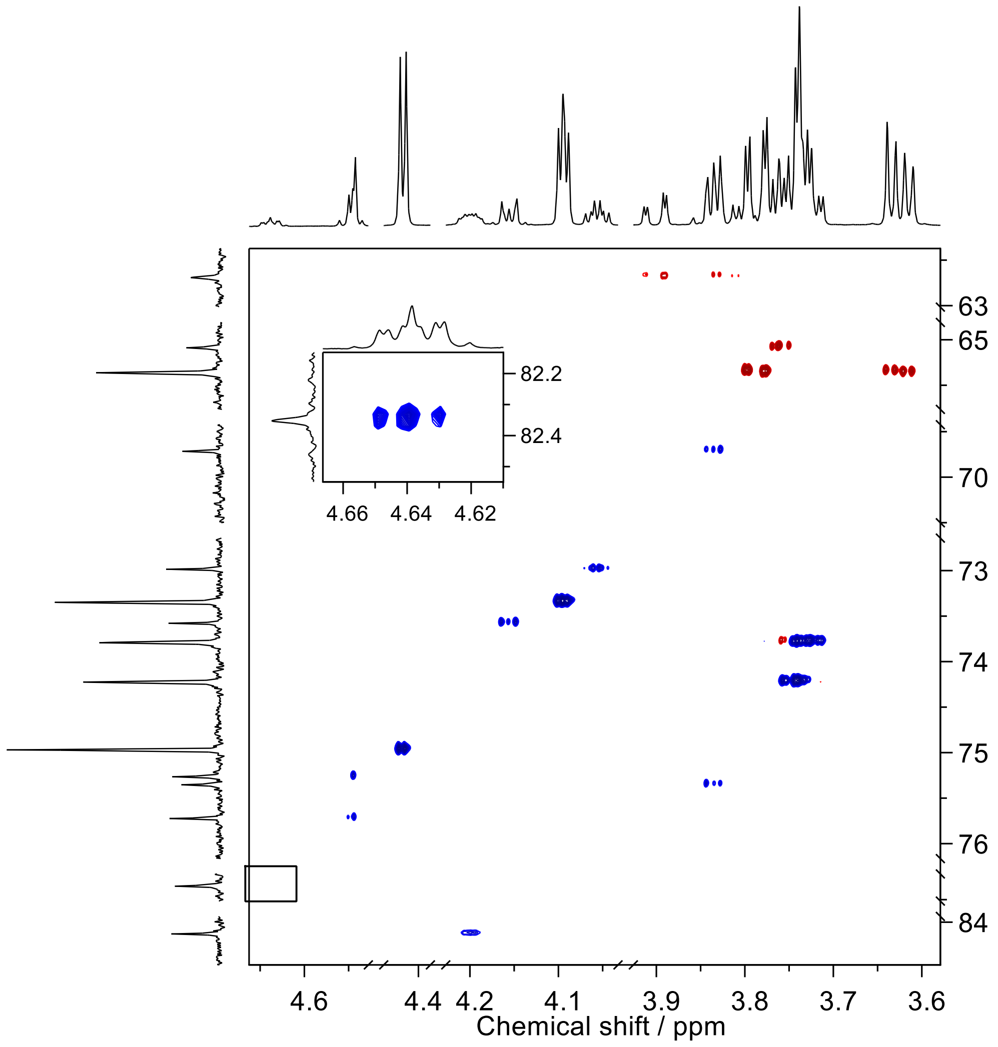

Figure S15. H,C-HSQC NMR spectrum obtained from 30 mM gluconic acid aqueous pH 1.1 solution containing 10% (v/v) D_2O, proving the additional signals arising from lactonization reactions at acid medium. For better visualization, only spectral regions of interest are shown; the insert depicts a magnification of the indicated area with the correlation signal being weak due to proximity to the water signal being suppressed by pre-saturation. Signals corresponding to CH groups are displayed in blue, those of (diastereotopic) CH_2 groups in red.

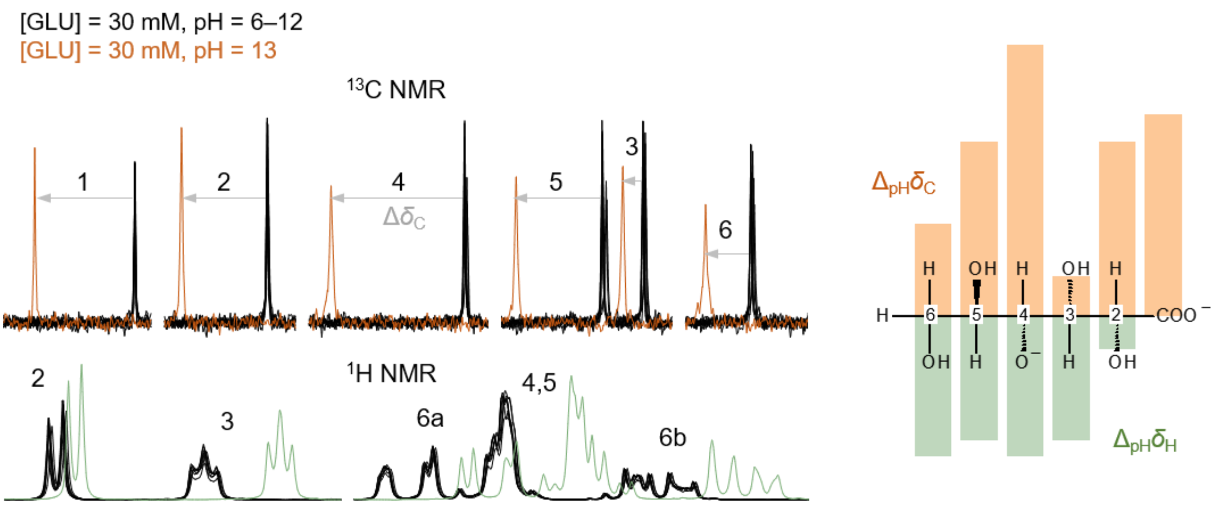

Figure S16. Schematic visualizing GLU's spectral behavior under strongly alkaline conditions. Left: in the range $6 \leq \mathrm{pH} \leq 12$, the black $^{13}C\{^{1}H\}$ (top) and ^{1}H NMR spectra (bottom) are virtually unaffected and only show alterations at pH 13 (spectra in orange and green, respectively). Right: signal shifts (to scale) of carbons and their attached hydrogens upon changing pH from 12 to 13 illustrate the speciation change, i.e., H^{+} abstraction, to occur at C4–O*H*.

Ln(III)-to-GLU titration series

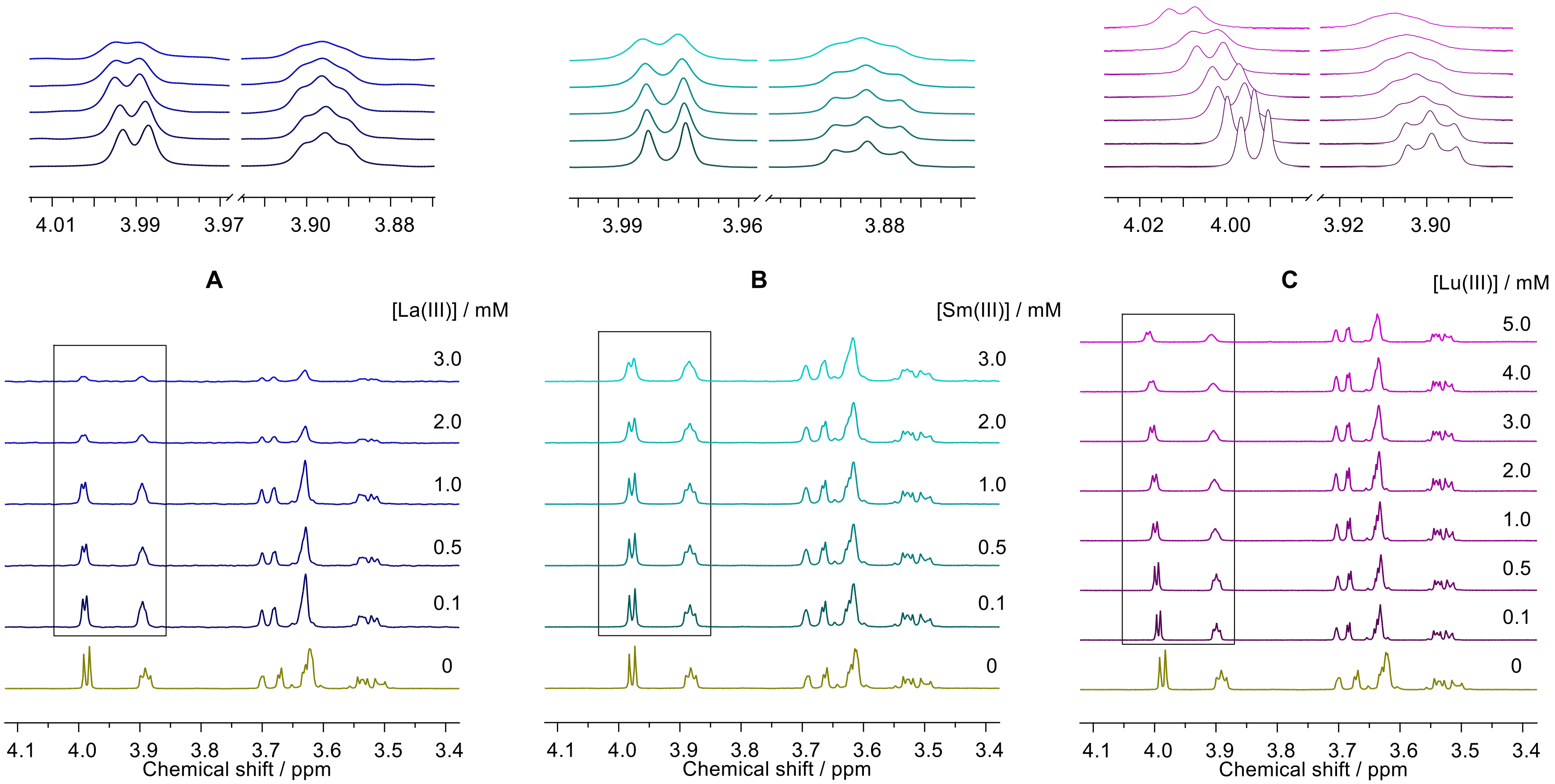

Figure S17. ^{1}H NMR spectra obtained from 5 mM gluconic acid in aqueous solution containing 10% (v/v) D_2O at pH 10 in presence of varying concentrations of La^{3+} (A), Sm^{3+} (B), or Lu^{3+} (C) as stated with the spectra. Dark yellow spectra in the bottom row represent Ln-free blank solutions obtained, along with those of the Sm(III) samples, at 400 MHz, whereas spectra of La/Lu solutions were acquired at 600 MHz. For better visualization, the spectra only show regions of interest; the inserts above depict a magnification of the indicated areas.

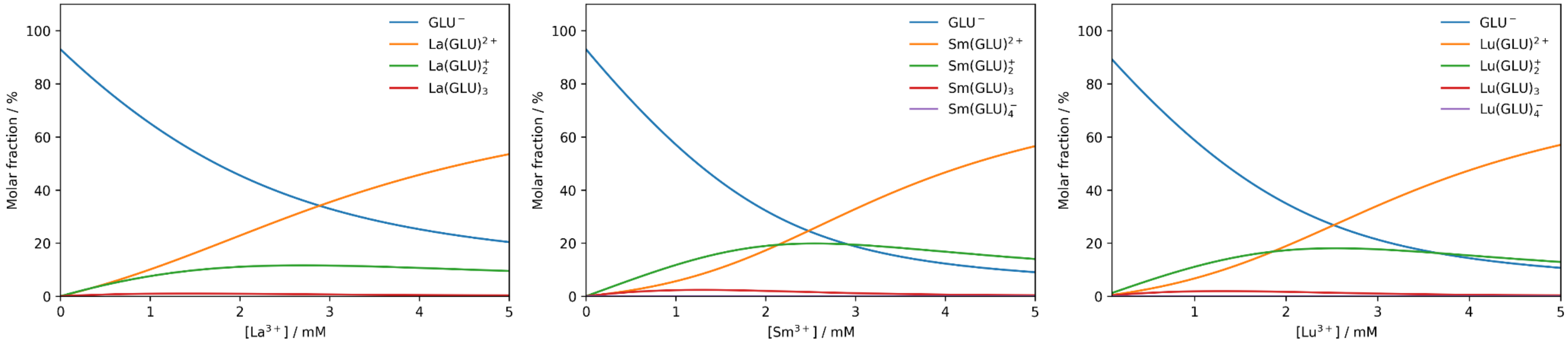

Figure S18. Speciation diagrams calculated for the experimental parameters of the Ln(III)-to-GLU titration series (5 mM GLU, pH 5) using thermodynamic data produced in this work (Table 7) and the ThermoChimie database V12.a Davies.[1]

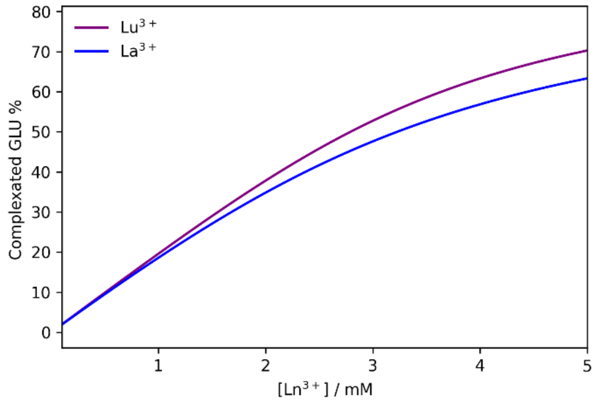

Figure S19. Comparison of percentage of complexed GLU as function of $[Ln^{3+}]$ (5 mM GLU, pH 5) for La and Lu based on speciation in Figure S18, SI.

GLU-to-Ln(III) titration series

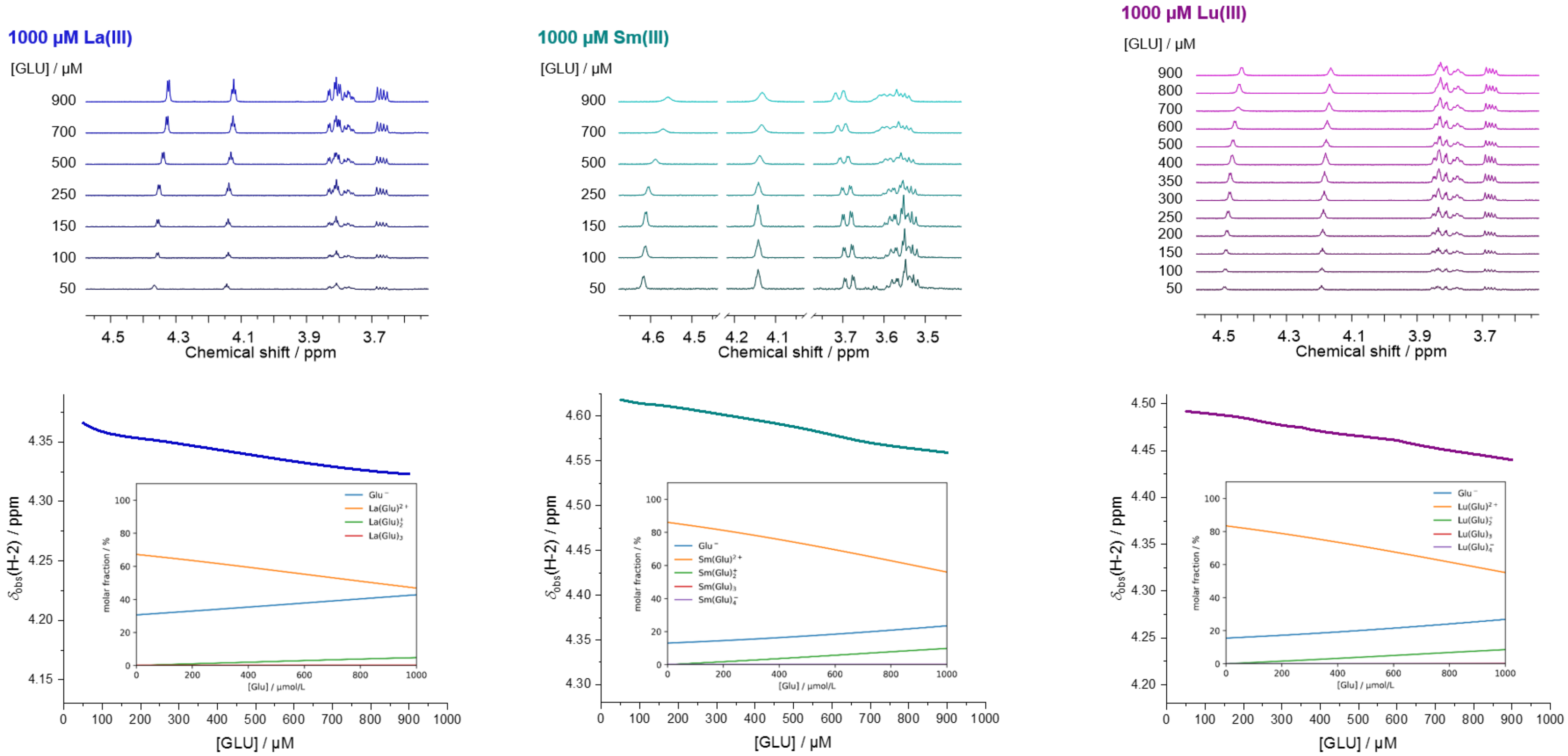

Figure S20. Top: ^{1}H NMR spectra obtained from pD 5 D_2O solutions in presence of varying gluconic acid concentrations as stated with the spectra for samples constant 1 mM in La(III) (left, blue), Sm(III) (middle, cyan), and Lu(III) (right, magenta). For better visualization, the spectra only show regions of interest. Bottom: Corresponding plots displaying the observed ^{1}H NMR chemical shift of H-2 in dependence on GLU concentration along with distribution of free and bound GLU species obtained from CE-ICP-MS analyses (for details, cf. Figure S21, SI).

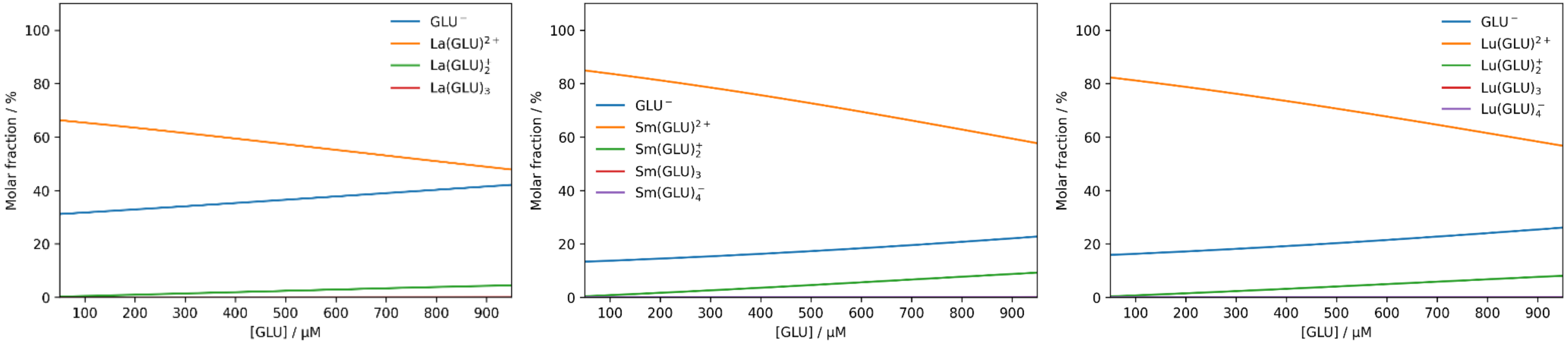

Figure S21. Speciation diagrams calculated for the experimental parameters of the GLU-to-Ln(III) titration series (1 mM Ln(III)) using thermodynamic data produced in this work (Table 7) and the ThermoChimie database V12.a Davies.[1]

pH-titration series of GLU–Ln(III) systems

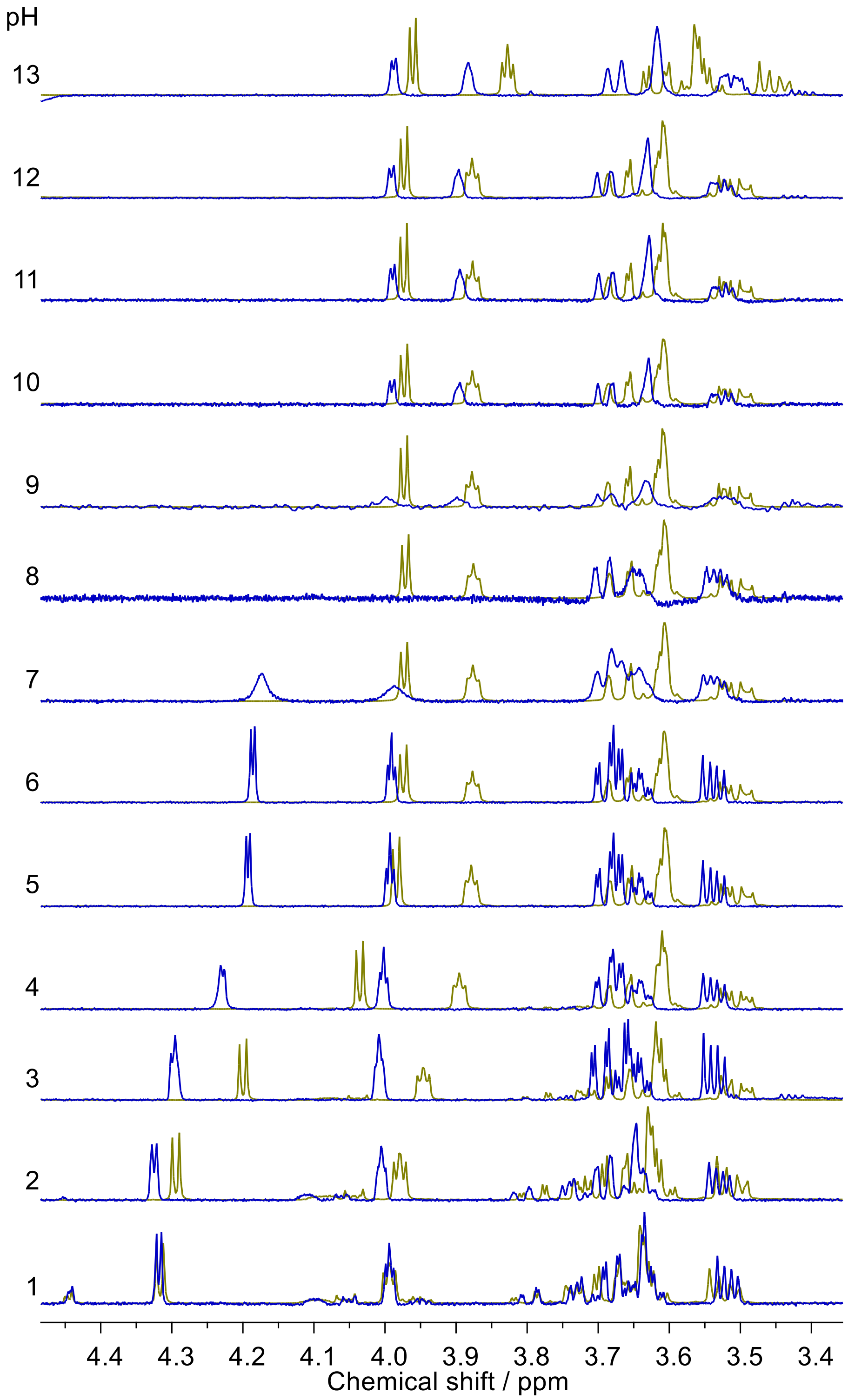

Figure S22. ^{1}H NMR spectra of pH-dependent aqueous solutions 1 mM each in gluconic acid and La(III) (blue spectra, obtained at 600 MHz) superimposed with gluconic acid blank spectra (dark yellow spectra, obtained at 400 MHz) for corresponding pH values.

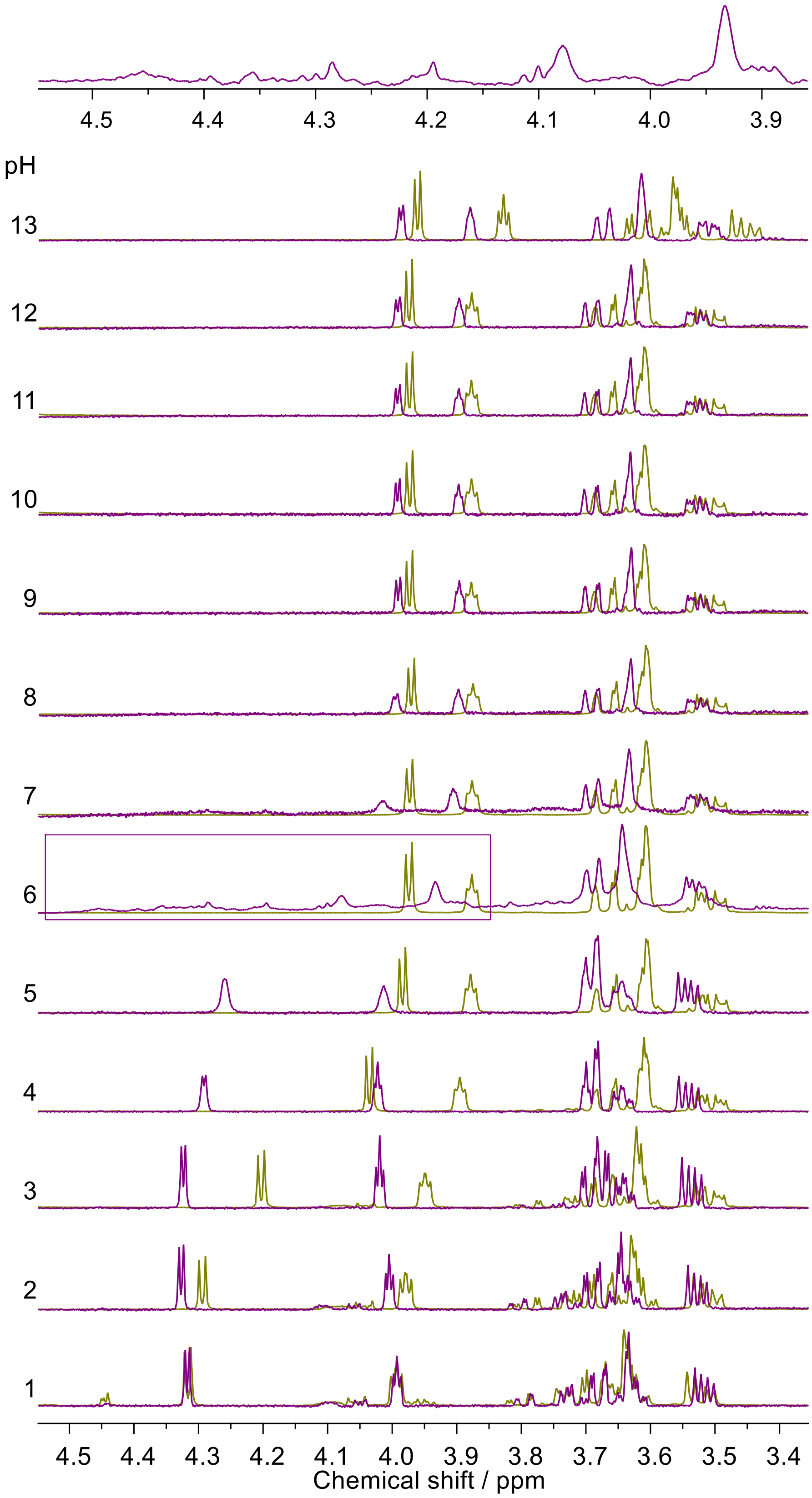

Figure S23. ^{1}H NMR spectra of pH-dependent aqueous solutions 1 mM each in gluconic acid and Lu(III) (magenta spectra, obtained at 600 MHz) superimposed with gluconic acid blank spectra (dark yellow spectra, obtained at 400 MHz) for corresponding pH values. A magnification of the Lu(III) containing pH 6 spectrum (indicated area) is shown at the top.

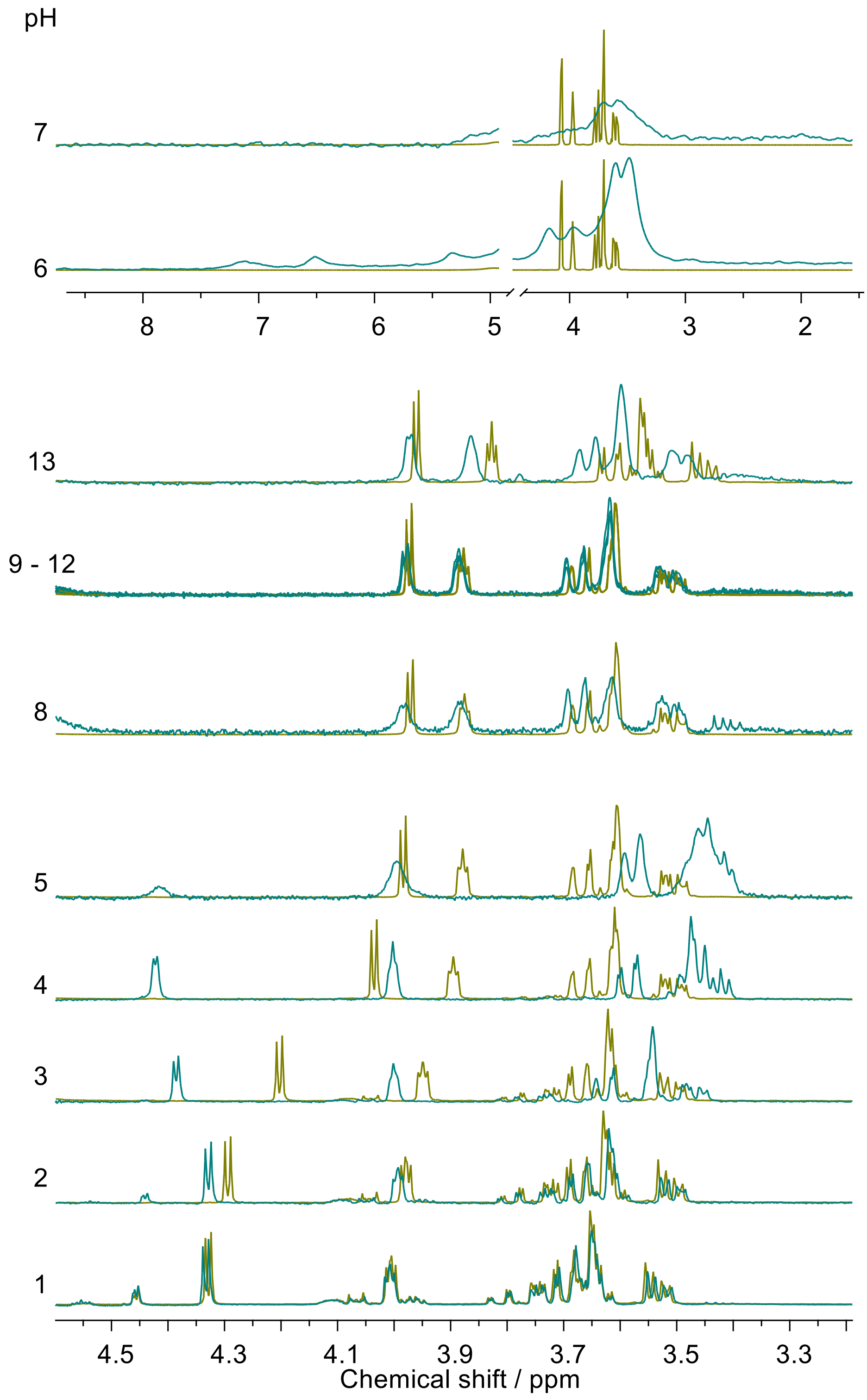

Figure S24. ^{1}H NMR spectra (obtained at 400 MHz) of pH-dependent aqueous solutions 1 mM each in gluconic acid and Sm(III) (cyan spectra) superimposed with gluconic acid blank spectra (dark yellow spectra) for corresponding pH values. For better visualization, the pH 6 and 7 spectra are displayed separately and because of virtually identical spectra for pH 9 through 12 spectra are superimposed all together.

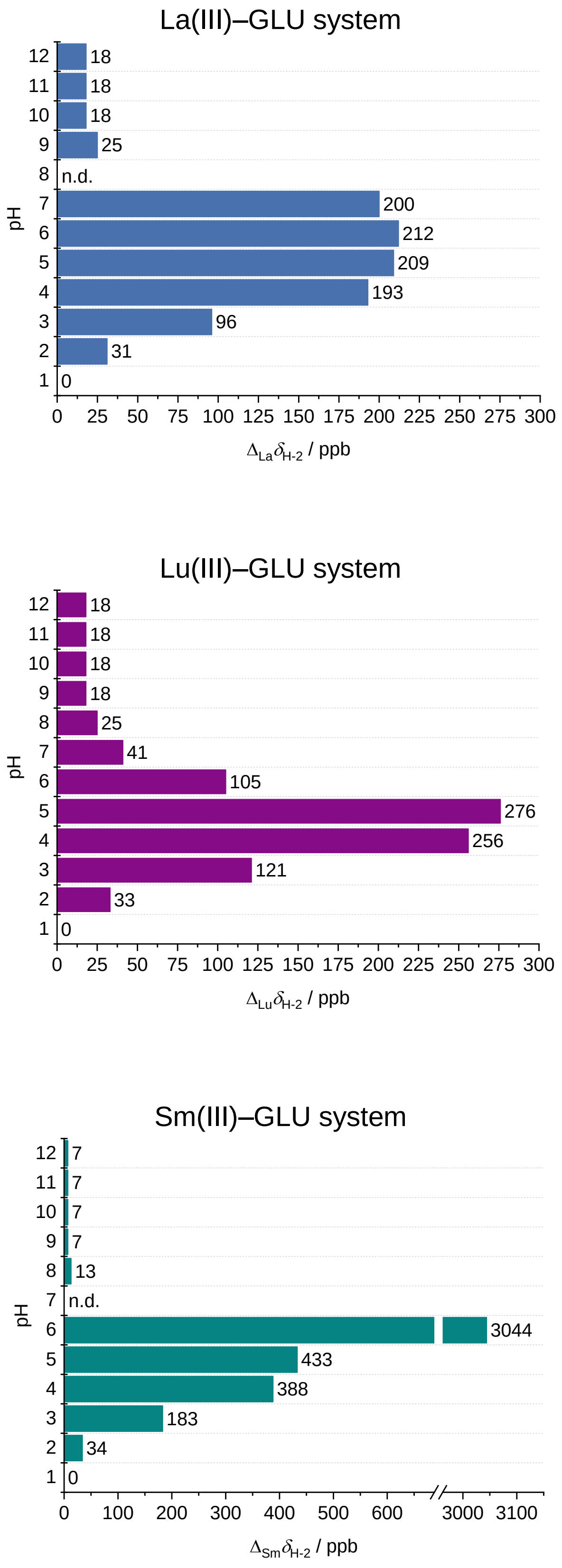

Figure S25. Graphical representation of Ln^{3+} complexation-induced ^{1}H NMR chemical shifts changes observed for H-2, corresponding to the spectral series depicted in Figures S22–S24, SI. In case of extreme line broadening the signal could not be detected (n.d.).

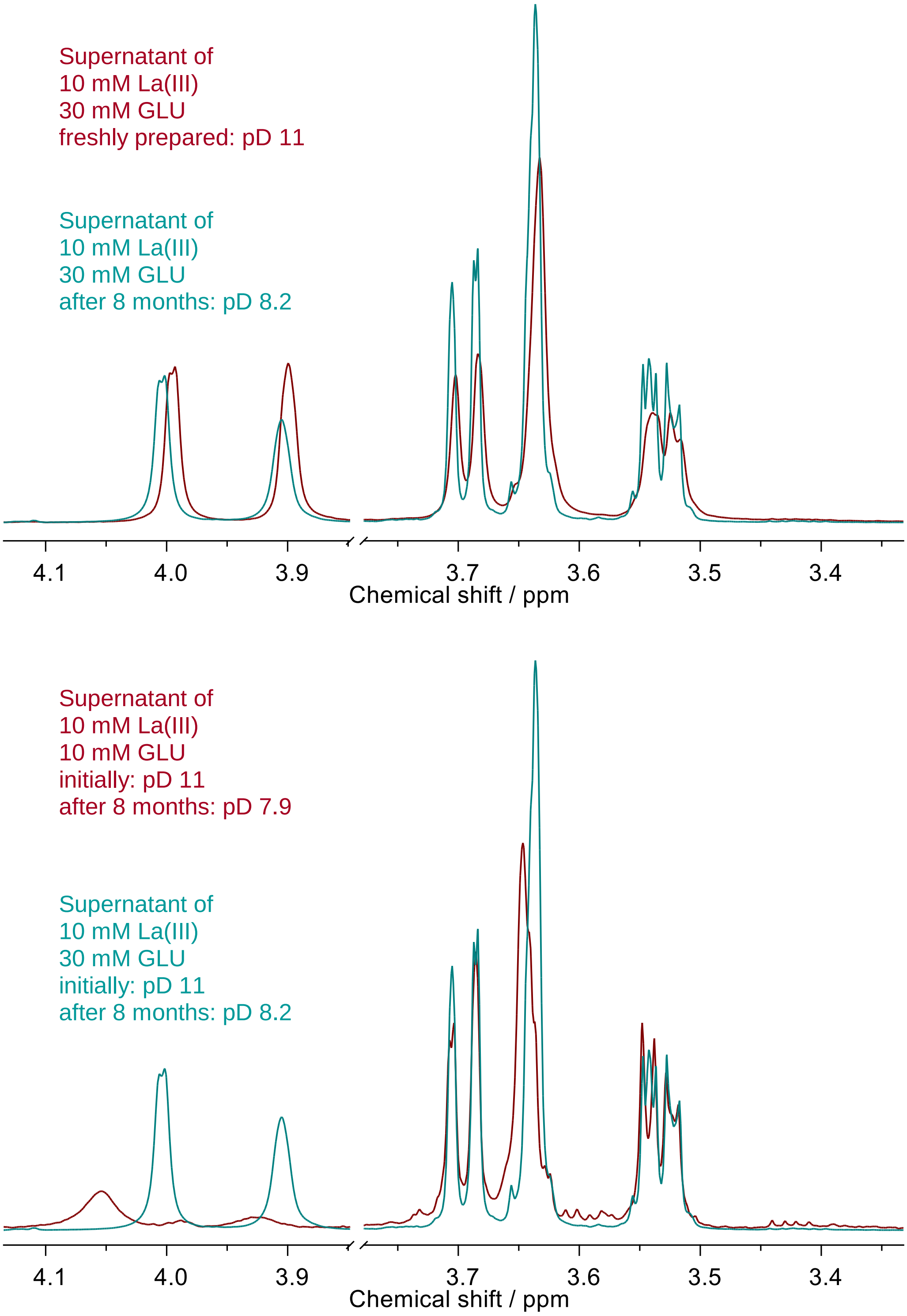

Figure S26. Top: ^{1}H NMR spectra obtained from the supernatants of solutions initially 10 mM in La(III) and 30 mM in GLU, freshly prepared (red, pD 11) and after eight months untouched (blue, pD 8.2). Bottom: ^{1}H NMR spectra obtained from the supernatants of solutions initially 10 mM in La(III) and either 10 mM in GLU (red, pD 7.9) or 30 mM GLU (blue, pD 8.2) untouched for eight months after being initially adjusted to pD 11.

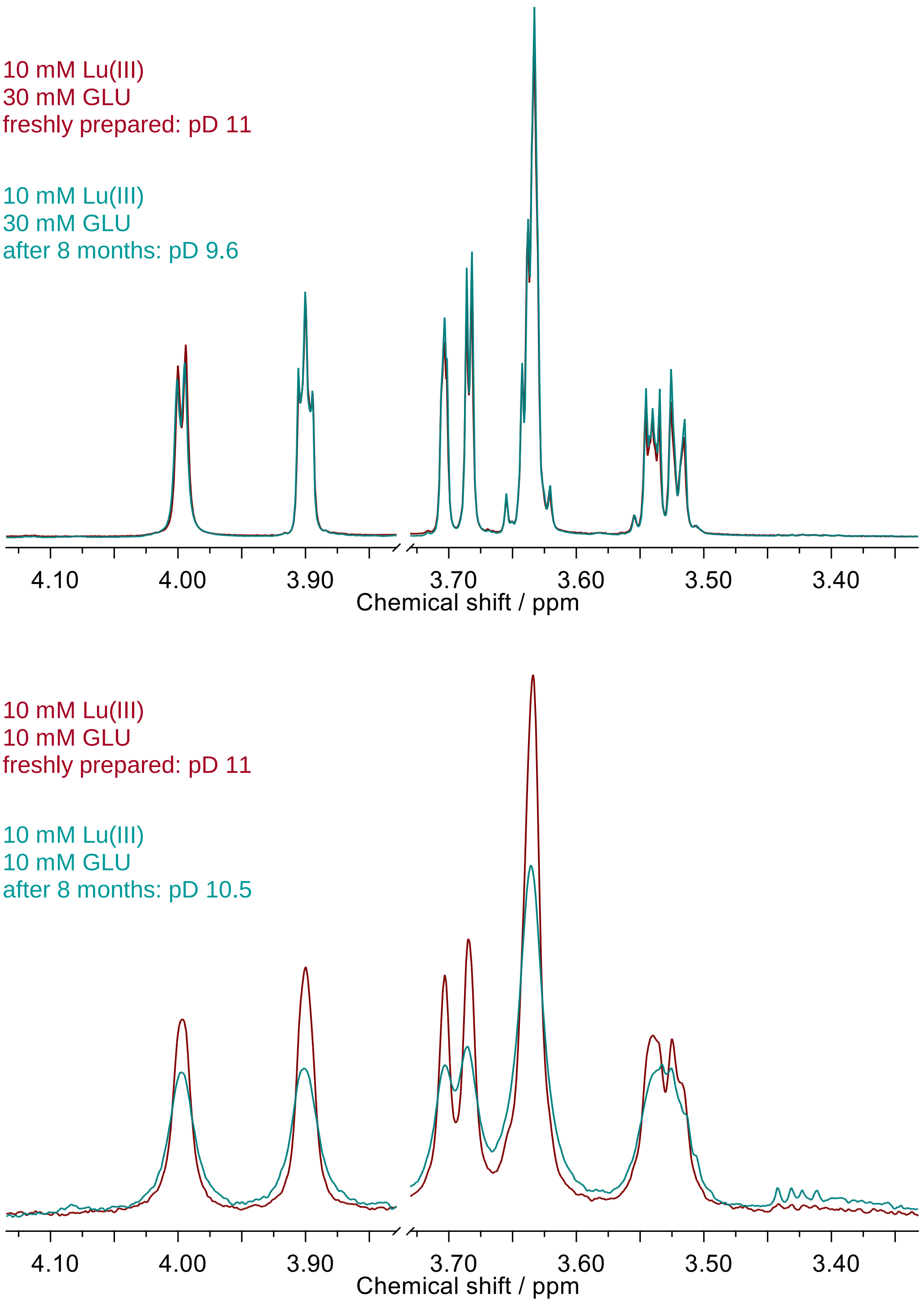

Figure S27. Top: ^{1}H NMR spectra obtained from solutions initially 10 mM in Lu(III) and 30 mM in GLU, freshly prepared (red, pD 11) and after eight months untouched (blue, pD 9.6). Bottom: ^{1}H NMR spectra obtained from solutions initially 10 mM in Lu(III) and 10 mM in GLU, freshly prepared (red, pD 11) and after eight months untouched (blue, pD 10.5).

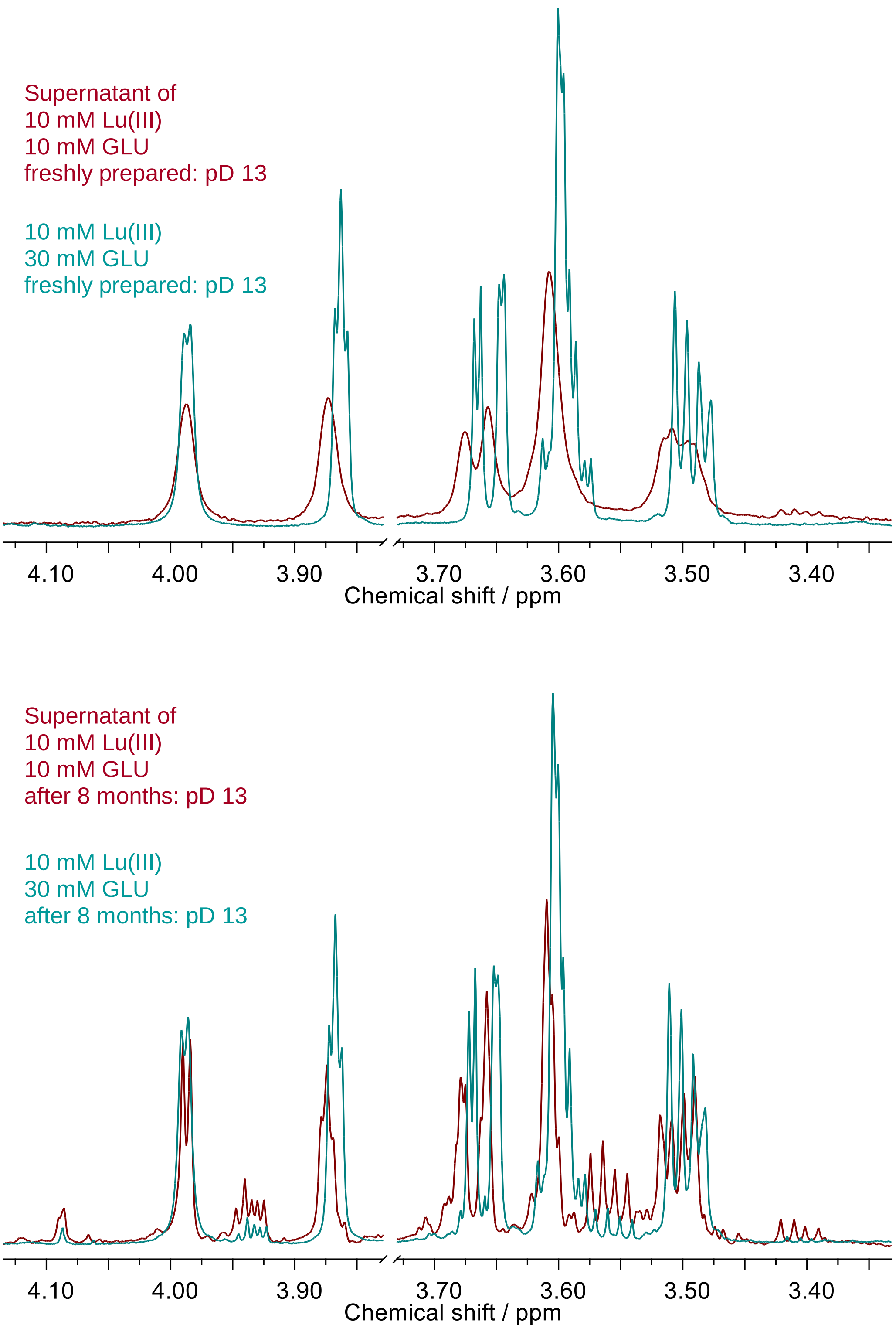

Figure S28. ^{1}H NMR spectra obtained from pD 13 solutions initially 10 mM in Lu(III) and either 10 mM (red spectra) or 30 mM in GLU (blue spectra), freshly prepared (top) and after eight months untouched (bottom). Note that since the solutions of 1:1 molar ratio showed precipitation, spectra refer to the corresponding supernatants.

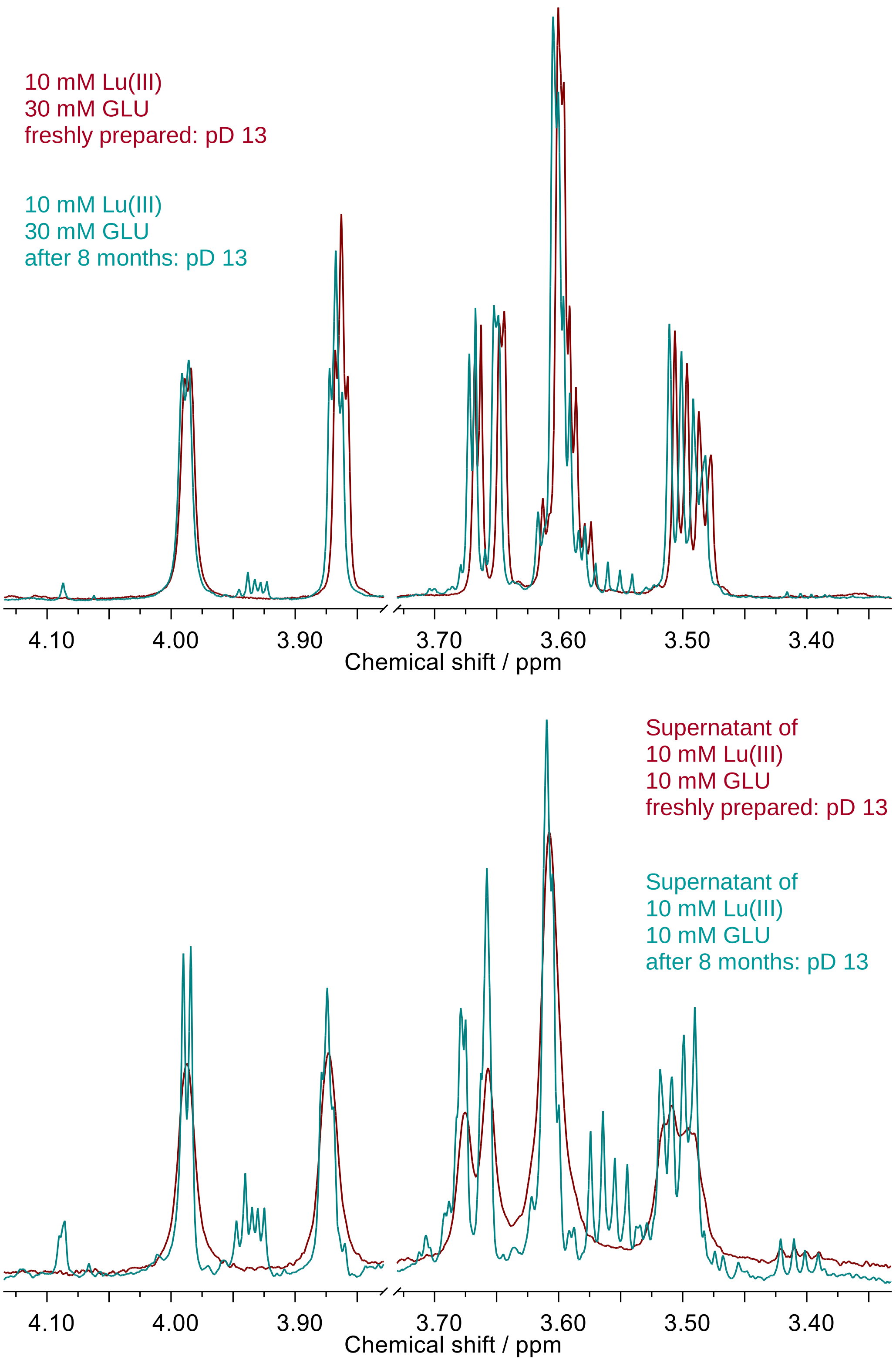

Figure S29. Top: ^{1}H NMR spectra obtained from pD 13 solutions initially 10 mM in Lu(III) and 30 mM in GLU, freshly prepared (red) and after eight months untouched (blue). Bottom: ^{1}H NMR spectra obtained from supernatants of solutions initially 10 mM in Lu(III) and 10 mM in GLU, freshly prepared (red) and after eight months untouched (blue).

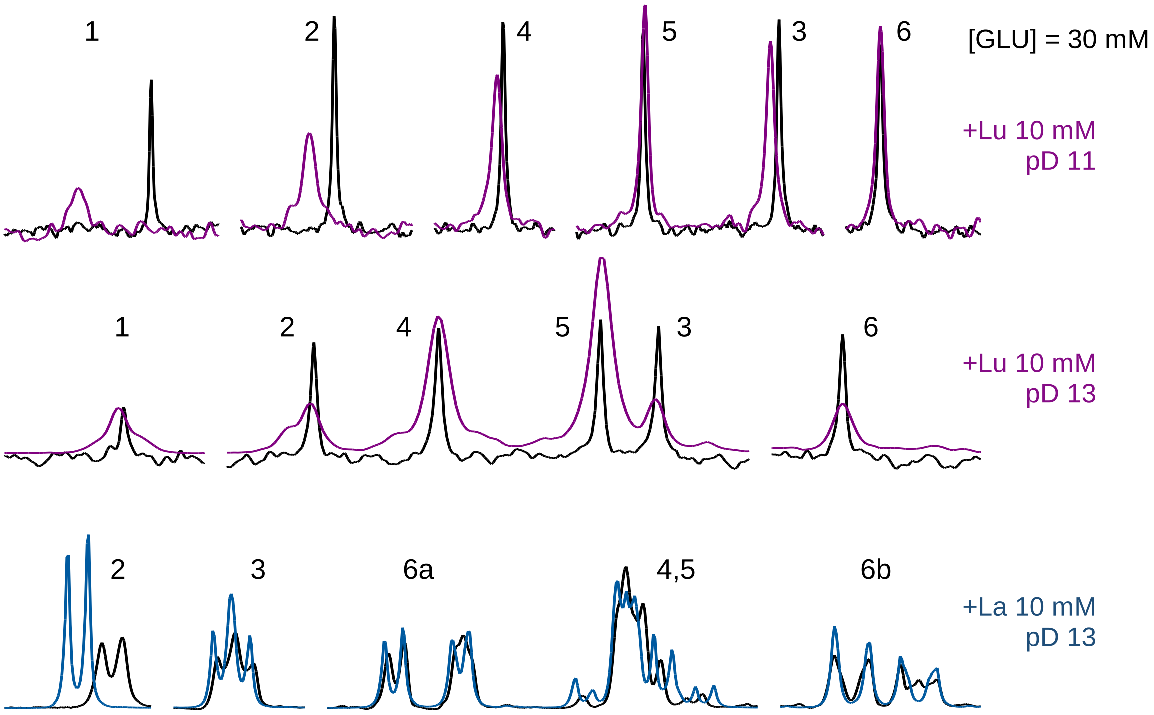

Figure S30. Schematic depicting spectral effects for 30 mM GLU solutions containing 10 mM La(III) (blue, ^{1}H NMR) and 10 mM Lu(III) (magenta, ^{13}C NMR) superimposed with spectra of GLU blanks at corresponding pD (black).

4. *Density functional (DF) calculations*

Table S5. Gibbs free energies of deprotonation reactions in gluconic acid and relative Gibbs free energies ΔG_{rel} of the second deprotonation for various hydroxyl groups (in kJ/mol).

Reaction		ΔG	$\Delta G_{rel\ 1*4*}$
GLU + H_2O = GLU^{-} + H_3O^{+}	1*	76.2	
GLU^{-} + H_2O = $GLUH_{-1}^{2-}$ + H_3O^{+}	1*4*	193.9	0.0
	1*5*		3.2
	1*3*		4.9
	1*6*		25.0
	1*2*		36.1

The asterisk marks the position of the deprotonated sites (1: COOH; 2–6: hydroxyl group in $GLUH_{-1}^{2-}$).

Table S6. Comparison of structural parameters[a] (in pm) and relative energies[b] (in kJ/mol) of La^{3+} aqua and monohydroxo complexes.

compound	CN[c]	$La\text{-}O_{aq}$	$La\text{-}O_{hyd}$	NHB[d]	ΔG_{rel}
$[La(H_2O)_9]^{3+}$	9	259			**0**
$[La(H_2O)_8]^{3+}$	8	255			9
$[La(H_2O)_7]^{3+}$	7	253			23
$[La(OH)(H_2O)_8]^{2+}$	9	263	242	2	2
$[La(OH)(H_2O)_7]^{2+}$	8	261	229	1	3
$[La(OH)(H_2O)_6]^{2+}$	7	259	221	0	**0**
$[La(OH)(H_2O)_5]^{2+}$	6	256	220	0	20

[a] Average bond lengths of La to aqua ligands, $La\text{-}O_{aq}$, and La to hydroxo group; $La\text{-}O_{hyd}$ [b] Relative Gibbs free energies. [c] Coordination number. [d] Number of intramolecular hydrogen bonds NHB.

Table S7. Comparison of structural parameters[a] (in pm) and relative energies[b] (in kJ/mol) of various coordination modes of gluconate in the complexes $[LaGLU(H_2O)_n]^{2+}$.

Coordination[c]	CN	n	$La\text{-}O_{aq}$	$La\text{-}O_{car}$	$La\text{-}O_{hyd}$	$La\text{-}O_{av}$	NHB	ΔG_{rel}
chel-123	9	6	260	250	263	259.6	3 (2)	**0**
	8	5	257	245	260	256.1	3 (2)	6
	10	7	265	251	270	264.7	1 (1)	27
chel-12	9	7	262	248	257	259.4	4 (3)	7
	8	6	258	243	257	255.9	4 (3)	8
mono	9	8	262	247		260.0	6 (3)	9
	8	7	258	241		256.0	5 (2)	16
	7	6	241	255		253.3	5 (2)	27
bi	8	6	257	256		256.9	3 (2)	29
	9	7	261	259		260.1	3 (2)	33
	7	5	254	253		253.8	3 (2)	37
chel-13	9	7	261	248	267	260.8	5 (3)	41
	8	6	258	242	265	257.2	5 (3)	45
chel-124	9	6	260	245	264	259.4	1 (1)	37
	8	5	257	240	263	256.3	1 (1)	50

[a] Average bond lengths of La to aqua ligands, $La\text{-}O_{aq}$; Bond lengths of La to carboxyl oxygens of gluconate, $La\text{-}O_{car}$; Bond lengths of La to coordinated hydroxo groups of gluconate, $La\text{-}O_{hyd}$; Average La-O bond lengths, $La\text{-}O_{av}$; Number of intramolecular hydrogen bonds NHB. In parentheses are noted the number of hydrogen bonds within the gluconate ligand, the others are between gluconate and coordinated aquo ligands. [b] Relative Gibbs free energies. [c] For details refer to Figure 8 in the main text.

Table S8. Relative Gibbs free energies (in kJ/mol) for various isomers of the complexes $[LaGLUH_{-1}(H_2O)_n]^+$ and $[LaGLU(OH)(H_2O)_n]^+$.

	Coordination[a]	CN	n	ΔG_{rel}
$[LaGLUH_{-1}(H_2O)_n]^+$	chel-123*	9	6	**0**
		8	5	3
	chel-12*3	8	5	1
		9	6	11
	chel-12*	8	6	12
		7	5	18
		9	7	57
	chel-13*	9	7	32
$[LaGLU(OH)(H_2O)_n]^+$	chel-123-OH			
	OH *trans*	8	4	14
		9	5	25
	OH *cis*	8	4	20
		9	5	27
		10	6	34
	chel-12-OH			
	OH-*trans*	8	5	26
		9	6	30

[a] For details refer to Figure 8 in the main text. The asterisk marks the position of the deprotonated hydroxyl group in coordinated $GLUH_{-1}^{2-}$

Table S9. Relative Gibbs free energies (in kJ/mol) for various isomers of the neutral complexes $[LaGLUH_{-2}(H_2O)_n]^0$, $[LaGLUH_{-1}(OH)(H_2O)_n]^0$ and $[LaGLU(OH)_2(H_2O)_n]^0$.

	Coordination[a]	CN	n	ΔG_{rel}
$[LaGLUH_{-2}(H_2O)_n]^0$	chel-12*3*	9	6	25
	chel-12*3*	8	5	30
	chel-123*-4*	9	6	19
	chel-123*-4*	8	5	3
	chel-123*-6*	9	6	160
	chel-123*-6*	8	5	100
$[LaGLUH_{-1}(OH)(H_2O)_n]^0$	chel-123*-OH *cis*	9	5	7
		8	4	1
	chel-12*-OH *cis*	8	5	14
	chel-123*-OH *trans*	9	5	16
		8	4	**0**
	chel-12*3-OH *cis*	9	5	20
		8	4	13
	chel-12*3-OH *trans*	9	5	27
		8	4	21
	chel-12*-OH *trans*	8	5	38
$[LaGLU(OH)_2(H_2O)_n]^0$	chel-123-OH *cis* OH *trans*	9	4	65
		8	3	13

[a] For details refer to the Figure 8 in the main text. The asterisk marks the position of the deprotonated hydroxyl groups in coordinated $GLUH_{-1}^{2-}$ and $GLUH_{-2}^{3-}$.

References

(1) Giffaut, E.; Grivé, M.; Blanc, P.; Vieillard, P.; Colàs, E.; Gailhanou, H.; Gaboreau, S.; Marty, N.; Madé, B.; Duro, L. Andra thermodynamic database for performance assessment: ThermoChimie. *Appl. Geochem.* **2014**, *49*, 225-236. DOI: 10.1016/j.apgeochem.2014.05.007.
